# Gut microbiota regulates blood‐cerebrospinal fluid barrier function and Aβ pathology

**DOI:** 10.15252/embj.2022111515

**Published:** 2023-07-10

**Authors:** Junhua Xie, Arnout Bruggeman, Clint De Nolf, Charysse Vandendriessche, Griet Van Imschoot, Elien Van Wonterghem, Lars Vereecke, Roosmarijn E Vandenbroucke

**Affiliations:** ^1^ VIB Center for Inflammation Research Ghent Belgium; ^2^ Department of Biomedical Molecular Biology Ghent University Ghent Belgium; ^3^ Department of Neurology Ghent University Hospital Ghent Belgium; ^4^ Department of Internal Medicine and Pediatrics Ghent University Ghent Belgium; ^5^ Ghent Gut Inflammation Group (GGIG) Ghent University Ghent Belgium

**Keywords:** Alzheimer's disease, blood‐cerebrospinal fluid barrier, gut microbiota, short‐chain fatty acids, vagus nerve, Immunology, Neuroscience

## Abstract

Accumulating evidence indicates that gut microbiota dysbiosis is associated with increased blood–brain barrier (BBB) permeability and contributes to Alzheimer's disease (AD) pathogenesis. In contrast, the influence of gut microbiota on the blood‐cerebrospinal fluid (CSF) barrier has not yet been studied. Here, we report that mice lacking gut microbiota display increased blood‐CSF barrier permeability associated with disorganized tight junctions (TJs), which can be rescued by recolonization with gut microbiota or supplementation with short‐chain fatty acids (SCFAs). Our data reveal that gut microbiota is important not only for the establishment but also for the maintenance of a tight barrier. Also, we report that the vagus nerve plays an important role in this process and that SCFAs can independently tighten the barrier. Administration of SCFAs in *App* ^NL‐G‐F^ mice improved the subcellular localization of TJs at the blood‐CSF barrier, reduced the β‐amyloid (Aβ) burden, and affected microglial phenotype. Altogether, our results suggest that modulating the microbiota and administering SCFAs might have therapeutic potential in AD via blood‐CSF barrier tightening and maintaining microglial activity and Aβ clearance.

## Graphical abstract

The gut microbiota promotes blood‐cerebrospinal fluid (CSF) barrier integrity by regulating the subcellular localization of tight junctions and reduces Alzheimer's disease pathology.

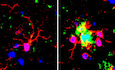

## Introduction

Gut microbiota are microorganisms, including bacteria, archaea, viruses, fungi, and protozoa, which live in the digestive tracts in a symbiotic relationship with the host. In the past decade, gut microbiota have been extensively studied and have shown their importance for many biological functions in the body, including intestinal development, barrier integrity and function (Hooper, 2004; Backhed *et al*, 2005), metabolism (Visconti *et al*, 2019; Agus *et al*, 2021), and regulation of peripheral and central immune responses (Hooper *et al*, 2012; Sharon *et al*, 2016). Although the mechanism of communication between the gut microbiota and the CNS is not fully understood, four principal signaling pathways have been proposed, including immune, endocrine, neural, and humoral routes (Dalile *et al*, 2019). Via these routes, the gut microbiota can (in)directly affect brain physiology such as neurogenesis, myelination, microglia activation, and regulation of neurotransmitters and neurotrophic factors such as brain‐derived neurotrophic factor and nerve growth factor (Diaz Heijtz *et al*, 2011; Sharon *et al*, 2016).

The development of the CNS also includes the formation of tightly closed blood–brain interfaces that ensure an optimal microenvironment for central nervous system (CNS) functions by restricting both paracellular and transcellular diffusion of hydrophilic and lipophilic substances (Strazielle & Ghersi‐Egea, 2013). One of the important blood–brain interfaces is the blood–brain barrier (BBB), located at the endothelium of the brain microvessels sealed by tight junctions (TJs), astrocytes, and pericytes (Strazielle & Ghersi‐Egea, 2013). There is accumulating evidence that the gut microbiota can affect the integrity of the BBB, with both broad‐spectrum antibiotic‐treated (AB) and germ‐free (GF) mice exhibiting significantly enhanced BBB permeability and dysregulation of inter‐endothelial cell TJs (Braniste *et al*, 2014; Frohlich *et al*, 2016; Hoyles *et al*, 2018). The mechanism(s) by which gut microbes exert their influence on BBB function are unclear, but changes to brain physiology induced by the alteration of the gut microbiota can occur independently via vagal or sympathetic neural pathways in the absence of any immune response, indicating at least partial contribution of soluble microbial‐derived metabolites (Bercik *et al*, 2011). Short‐chain fatty acids (SCFAs), the main metabolites produced by colonic fermentation of dietary fibers and resistant starch, have been shown to improve the BBB integrity by upregulation of TJ expression *in vitro* and *in vivo* (Parker *et al*, 2020). For example, butyrate supplementation has rescued the BBB disruption in GF mice by upregulating the expression of Occludin (OCLN) and Claudin‐5 (CLDN‐5; Braniste *et al*, 2014). In a cell culture model, propionate has been found to attenuate the lipopolysaccharide (LPS)‐induced BBB disruption by upregulating the expression of CLDN‐5 and Zona occludens‐1 (ZO‐1) (Hoyles *et al*, 2018). SCFAs may access the BBB via the bloodstream to directly have an impact on its integrity (Macfabe, 2012) or indirectly affect BBB function by activating members of the free fatty acid receptor (FFAR) family such as FFAR2 and FFAR3 in endothelial cells (Alexander *et al*, 2015).

Another important, but often neglected, blood–brain interface is the blood‐cerebrospinal fluid (CSF) barrier, formed by choroid plexus epithelial (CPE) cells which are tightly interconnected via the presence of TJs (Strazielle & Ghersi‐Egea, 2013). There is a growing body of evidence showing that the blood‐CSF barrier plays a crucial role in the spread of inflammatory reactions from the periphery to the CNS and contributes to the pathogenesis and progression of various neurological disorders (Baruch *et al*, 2015; Brkic *et al*, 2015; Gorle *et al*, 2018; Steeland *et al*, 2018; Rodriguez‐Lorenzo *et al*, 2020; Van Hoecke *et al*, 2021; Xie *et al*, 2021).

Here, we assessed the impact of gut microbiota depletion, via AB treatment and in GF mice, on blood‐CSF barrier integrity in wild‐type and *App* ^*NL‐G‐F*^ Alzheimer's disease (AD) mice and this revealed that the microbiota is necessary to not only initiate the formation of the blood‐CSF barrier but also to maintain its integrity. Moreover, SCFAs play a vital role in regulating blood‐CSF barrier integrity, independent of the vagus nerve, and have the potential to reduce Aβ accumulation, one of the major hallmarks of AD.

## Results

### Gut microbiota affect gene profile of the choroid plexus

**Figure 1:**
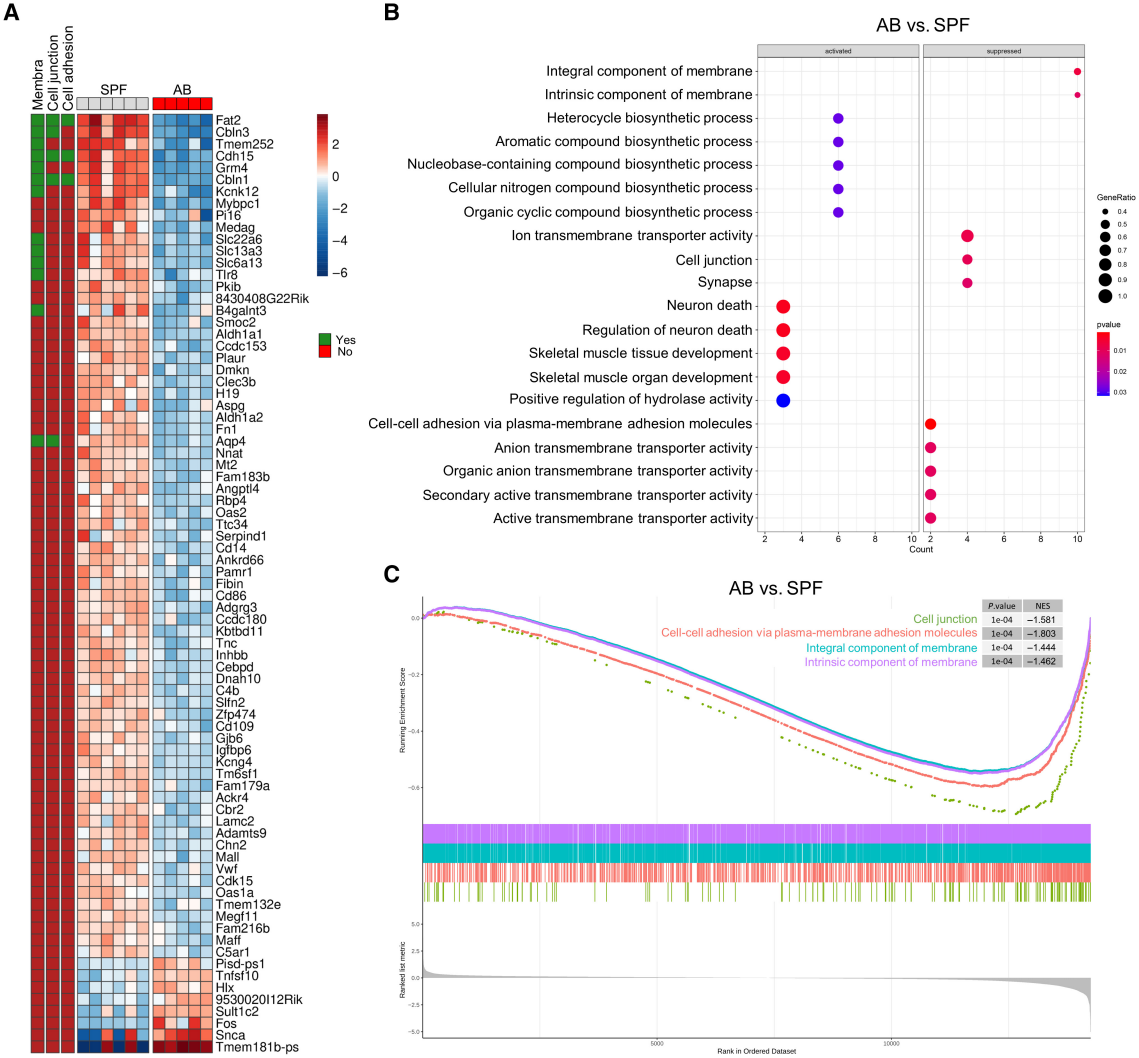
Altered gene profile of choroid plexus in mice with different gut microbiota composition AHeatmap of all the DE genes in choroid plexus between the AB and SPF mice. The color scale of the heatmap represents the scaled log2 normalized gene expression.BDot plot showing the top 10 significant up and downregulated GO categories (according to *P*‐value) ordered according to geneRatio. The *P*‐value and the number of genes of a gene set are represented by the color and the size of the dot, respectively.CGSEA plot for four GO terms performed on full gene distribution of comparison in choroid plexus between AB and SPF. The plot features the running enrichment scores and placement of the member genes for each respective GO term and also includes the ranked list metric plot for the full gene distribution. Data information: AB, antibiotics‐treated; GO, Gene Ontology; GSEA, Gene Set Enrichment Analysis; SPF, specific pathogen‐free;

**Figure EV1:**
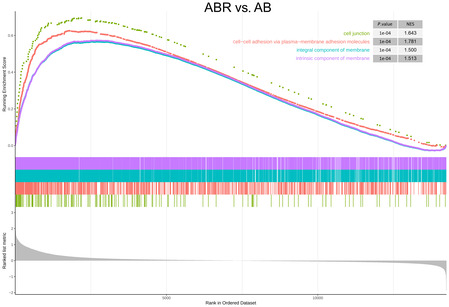
GSEA of the barrier function genes in recolonized antibiotics‐treated mice versus antibiotics‐treated mice Combined GSEA plot for four GO terms performed on full gene distribution of comparison in choroid plexus between AB and ABR mice. The plot features the running enrichment scores and placement of the member genes for each respective GO term and also includes the ranked list metric plot for the full gene distribution. AB, antibiotics‐treated; ABR, recolonized AB; GO, gene Ontology; GSEA, Gene Set Enrichment Analysis.

**Figure EV2:**
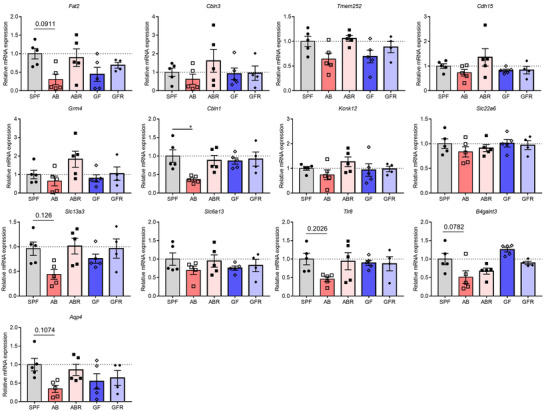
qPCR analysis for barrier function‐associated genes on choroid plexus of SPF, AB, ABR, GF, and GFR mice Data information: Bars represent mean ± SEM. *n* = 5, biological replicates. Statistics were performed with one‐way ANOVA Bonferroni's *post hoc* test for multiple comparisons. *p < 0.05. AB, antibiotics‐treated; ABR, recolonized AB; GF, germ‐free; GFR, recolonized GF; SPF, specific pathogen‐free.Source data are available online for this figure.

To determine the effects of gut microbiota on the blood‐CSF barrier, we first generated gut microbiota‐depleted mice by treating specific pathogen‐free (SPF) mice orally with broad‐spectrum antibiotics (AB mice) for 2 weeks. No bacterial growth was detected on BHI plate inoculated with the fecal solutions from AB mice (Appendix Fig S1A), and this was accompanied by markedly enlarged caeca (Appendix Fig S1B). RNA sequencing (RNA‐seq) analysis of choroid plexus tissues of normal colonized SPF *versus* decolonized AB mice revealed 79 differentially expressed genes (DEGs; adjusted *P*‐value < 0.05 and |logFC| > 1), of which eight genes were upregulated in AB and 71 genes were downregulated (Fig 1A; Dataset EV1). Among the downregulated genes in choroid plexus tissue from decolonized AB mice, we identified 13 genes that were linked to barrier function (Fig 1A). This results in a suppression of the Gene Ontology (GO) categories of cell junction, cell adhesion, and component of membrane (integral and intrinsic) according to Gene Set Enrichment Analysis (GSEA; Fig 1B; Dataset EV2), suggesting that the barrier function of choroid plexus is affected by the absence of gut microbiota in the AB mice. We further performed GSEA on the complete gene distribution of two comparisons (AB vs. SPF and recolonized AB mice [ABR] vs. AB) in choroid plexus, and this showed that the gene sets related to the abovementioned GO categories were highly negatively enriched in the AB vs. SPF comparison (Fig 1C; Dataset EV3). Strikingly, the same GO categories were positively enriched to the same extent in the ABR vs. AB comparison by comparing the normalized enrichment scores (NES; AB vs. SPF: NES = −1.444 ~ −1.803; ABR vs. AB: NES = 1.500 ~ 1.781; Fig EV1; Dataset EV4). Two additional comparisons were made within the RNA‐seq data: germ‐free mice (GF) vs. SPF and recolonized GF mice (GFR) vs. GF (Dataset EV1). The DEGs from each of the four comparisons were combined into one heatmap (Appendix Fig S2). To confirm the RNA‐seq results, we next analyzed the expression of RNA‐seq‐detected AB vs. SPF DEGs in the choroid plexus of SPF, AB, ABR, GF, and GFR via qPCR, focusing on the genes associated with barrier function. Consistent with the RNA‐seq results, *Cbln1* gene expression was significantly downregulated in AB mice compared with SPF mice, while all other tested DEGs showed only a trend toward (Fig EV2). In addition, this downregulation appeared to be suppressed to some extent upon gut microbiota reconstitution (ABR; Fig EV2). In GF mice, *Fat2*, *Tmem252*, *Slc13a3*, *Slc6a13*, and *Aqp4* showed trends of downregulation similar to AB mice, but none of these genes showed upregulation upon gut microbiota recolonization (GFR; Fig EV2). Altogether, this suggests that choroid plexus barrier function is reduced upon gut microbiota depletion and again rescued upon gut microbiota reconstitution.

### Lack of gut microbiota increases blood‐CSF barrier permeability

**Figure EV3:**
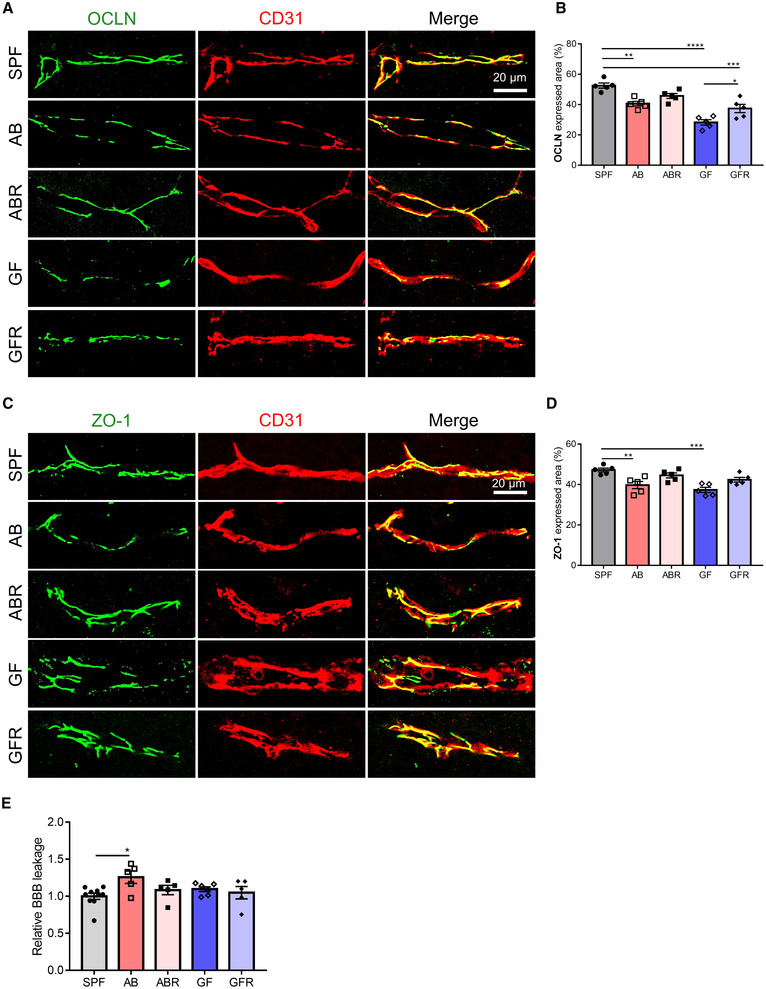
BBB integrity in mice with different gut microbiota composition ARepresentative images of immunostaining for OCLN and CD31 in cortex. Scale bar: 20 μm.BThe percentage of OCLN expressed area (*n* = 5, biological replicates).CRepresentative images of immunostaining for ZO‐1 and CD31 in cortex. Scale bar: 20 μm.DThe percentage of ZO‐1 expressed area (*n* = 5, biological replicates).EAssessment of the BBB permeability to 4 kDa FITC‐dextran (*n* = 5–10, biological replicates). Data information: Bars represent mean ± SEM. Statistics were performed with one‐way ANOVA Bonferroni's *post hoc* test for multiple comparisons. *p < 0.05, **p < 0.01, ***p < 0.001, ****p < 0.0001. BBB, blood–brain barrier. AB, antibiotics‐treated; ABR, recolonized AB; GF, germ‐free; GFR, recolonized GF; SPF, specific pathogen‐free. Source data are available online for this figure.

Next, we characterized BBB integrity by TJs immunostainings in the mice with different gut microbiota compositions, namely GF, GFR, AB, and ABR mice. In agreement with previous studies (Braniste *et al*, 2014; Sun *et al*, 2021), our immunofluorescence analysis confirmed lower expression and disrupted subcellular localization of occludin (OCLN) and zona occludens‐1 (ZO‐1) in the brain vessels of GF and AB mice compared with SPF mice (Fig EV3A–D). Reconstitution of gut microbiota in GF and AB mice revealed largely restored defects in expression and integrity of OCLN and ZO‐1 (Fig EV3A–D). In 4 kDa FITC‐dextran leakage analysis, BBB permeability was increased in AB mice compared with SPF mice, while this was not the case in ABR mice. However, the GF mice did not show an obvious BBB permeability response to 4 kDa FITC‐dextran (Fig EV3E). These results confirm that gut microbiota is important not only for the development of the BBB but also for the maintenance of BBB integrity.

**Figure 2:**
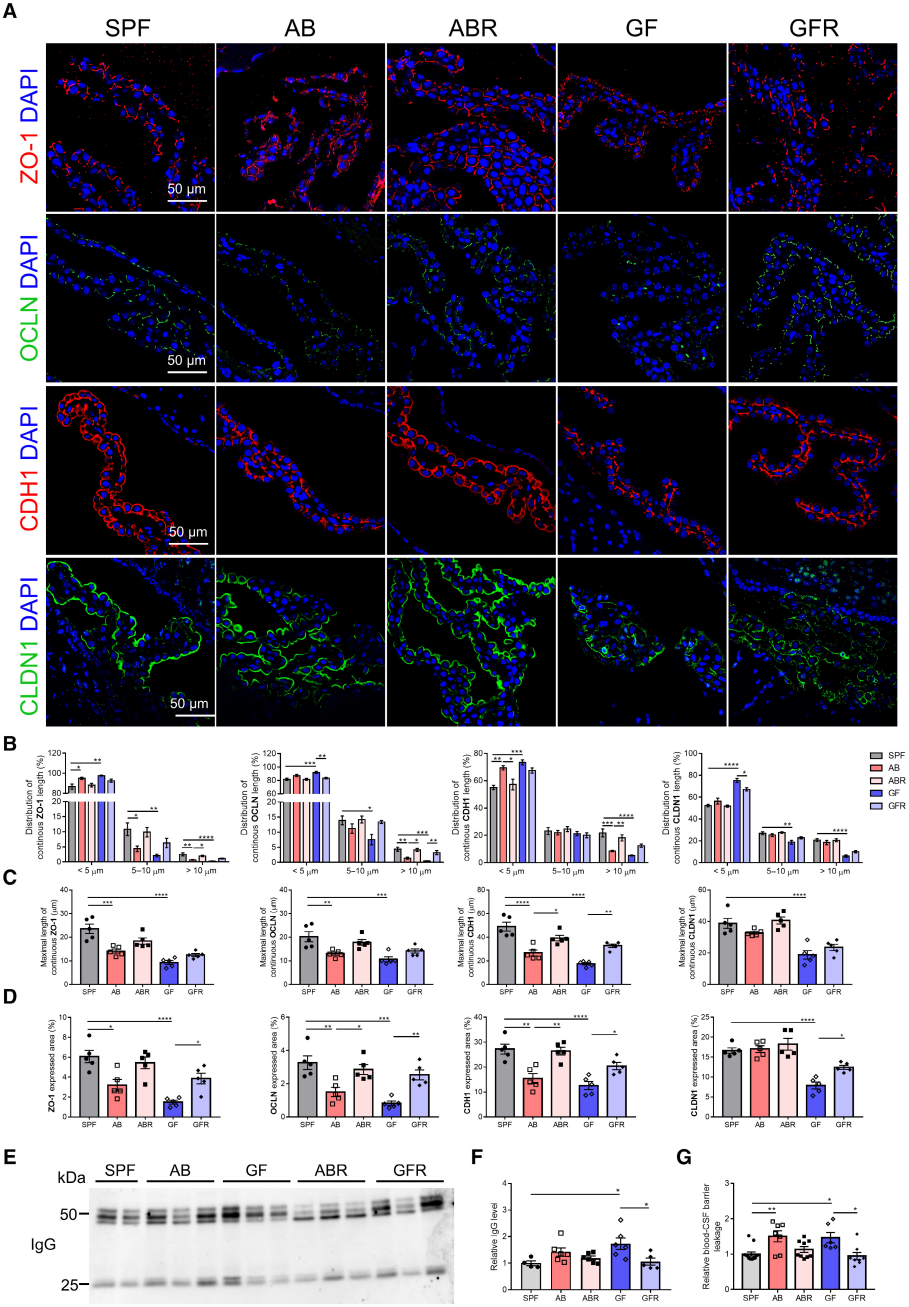
Blood‐CSF barrier integrity in mice with different gut microbiota composition ARepresentative images of immunostainings for ZO‐1, OCLN, CDH1, and CLDN1 in choroid plexus of SPF, AB, ABR, GF, and GFR mice. Scale bar: 50 μm.B–DQuantification of continuous length (B), maximal length (C), and expressed area (D) of the TJ immunostainings displayed in (A) (*n* = 5, biological replicates).ERepresentative western blot showing the IgG levels in CSF of SPF, AB, ABR, GF, and GFR mice.FQuantitative analysis of IgG in CSF via western blot in (E) (*n* = 4–6, biological replicates).GAssessment of the blood‐CSF barrier permeability to 4 kDa FITC‐dextran (*n* = 5–9, biological replicates). Data information: Bars represent mean ± SEM. Statistics were performed with one‐way ANOVA Bonferroni's *post hoc* test for multiple comparisons. *p < 0.05, **p < 0.01, ***p < 0.001, ****p < 0.0001. AB, antibiotics‐treated; ABR, recolonized AB; CSF, cerebrospinal fluid; GF, germ‐free; GFR, recolonized GF; SPF, specific pathogen‐free; TJ, tight junction. Source data are available online for this figure.

Similarly, TJ expression and their subcellular localization were studied at the blood‐CSF interface from SPF, GF, and GFR mice (Fig 2A–D). In SPF mice, ZO‐1, OCLN, E‐cadherin (CDH1), and claudin‐1 (CLDN1) immunoreactivity appeared as a near continuous staining at the apical cell border (Fig 2A). Notably, the GF mice displayed a fragmented border and diffuse distribution staining of ZO‐1, OCLN, CDH1, and CLDN‐1 proteins (Fig 2A). Moreover, lower continuous TJ length and downregulated TJ expression in GF mice compared with SPF mice was also verified by the quantification (includes distribution of continuous TJ length, maximal length of continuous TJ, and TJ expressed area; Fig 2B–D). Interestingly, GFR mice reversed this pattern to a limited extent compared with the SPF state (Fig 2A–D). Accordingly, the level of CSF immunoglobulin G (IgG), a marker of barrier permeability status (Shrestha *et al*, 2014), and the level of 4 kDa FITC‐dextran leakage was increased in GF mice and rescued after recolonization (Fig 2E–G). These findings indicate that gut microbiota is necessary for modulating BBB development.

Next, we investigated whether the removal of the gut microbiota after blood‐CSF barrier formation also influences its integrity. Notably, the AB mice exhibited delocalization of TJs of ZO‐1, OCLN, and CDH1 proteins compared with SPF mice, while CLDN‐1 was not affected (Fig 2A–D). Interestingly, ABR mice also reversed this pattern and similar TJs localization was observed compared with the SPF mice (Fig 2A–D). Consistent with this observation, the levels of CSF IgG and 4 kDa FITC‐dextran leakage were increased in AB mice, but to a lesser extent than the levels in GF mice. Similarly, compared with AB mice, ABR mice showed significantly lower levels of IgG in CSF (Fig 2F and G). Collectively, these data suggest that the gut microbiota is not only an initial trigger to form the blood‐CSF barrier, but a continuous contribution of gut microbiota is critical for maintaining its barrier integrity.

### SCFAs attenuate blood‐CSF barrier disruption caused by the absence of gut microbiota

**Figure 3:**
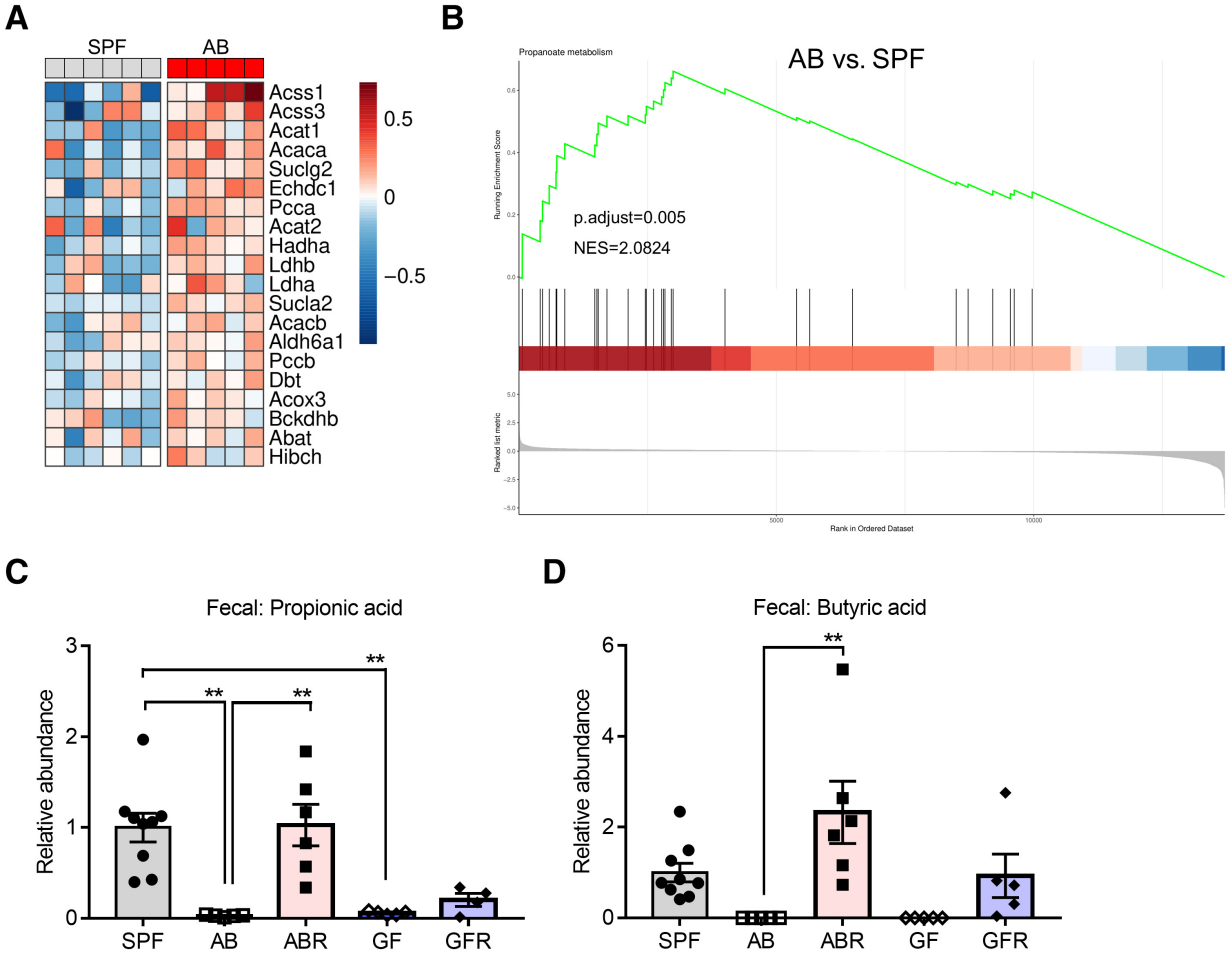
Effects of gut microbiota on SCFA metabolism in the gut and choroid plexus AHeatmap of the genes linked to the KEGG pathway mmu00640 (Propanoate metabolism) which are present in the DEGs list between the AB and SPF mice in choroid plexus. The color scale of the heatmap represents the scaled log2 normalized gene expression (*n* = 5–6, biological replicates).BCombined GSEA plot for KEGG pathway performed on full gene distribution of comparison in choroid plexus between AB and SPF. The plot features the running enrichment scores and placement of the member genes for the KEGG pathway and also includes the ranked list metric plot for the full gene distribution (*n* = 5–6, biological replicates).C, DRelative abundance of propionic acid (C) and butyric acid (D) in mice fecal samples (*n* = 4–9, biological replicates). Data information: Bars represent mean ± SEM. Statistics were performed with one‐way ANOVA Bonferroni's *post hoc* test for multiple comparisons. **p < 0.01. AB, antibiotics‐treated; DEGs, differentially expressed genes; SCFAs, short‐chain fatty acids; SPF, specific pathogen‐free. Source data are available online for this figure.

Short‐chain fatty acids, mainly including acetate, propionate, and butyrate, are metabolites produced during the bacterial fermentation of dietary fiber in the intestinal tract (Dalile *et al*, 2019). Propionate and butyrate are known to enhance the integrity of the BBB by facilitating TJ expression and assembly (Braniste *et al*, 2014; Hoyles *et al*, 2018). Hence, we studied whether propionate and butyrate might have the same beneficial effects in improving the blood‐CSF barrier integrity. Initially, we determined the enrichment of the propionate metabolism (KEGG pathway mmu00640) gene set on the full gene distribution comparing AB and SPF mice choroid plexus gene expression (Fig 3A and B; Dataset EV5). The RNA‐seq data were validated using qPCR and focusing on several top DEGs, including *Acss1*, *Acss3*, *Acat1*, *Acaca*, and *Suclg2*. Consistent with the RNA‐seq data, these genes showed upregulation in AB mice compared with SPF mice. However, this was only signification in case of *Acat1* (Appendix Fig S3). Interestingly, the propionate metabolism pathway seems to be activated in AB mice, which could be a compensatory mechanism caused by the depletion of SCFAs. Correspondingly, we detected that the contents of propionate and butyrate in the feces of AB and GF mice were significantly lower than those of SPF mice, while recolonization of AB and GF mice could restore the metabolism of propionate and butyrate to varying degrees (Fig 3C and D).

**Figure 4:**
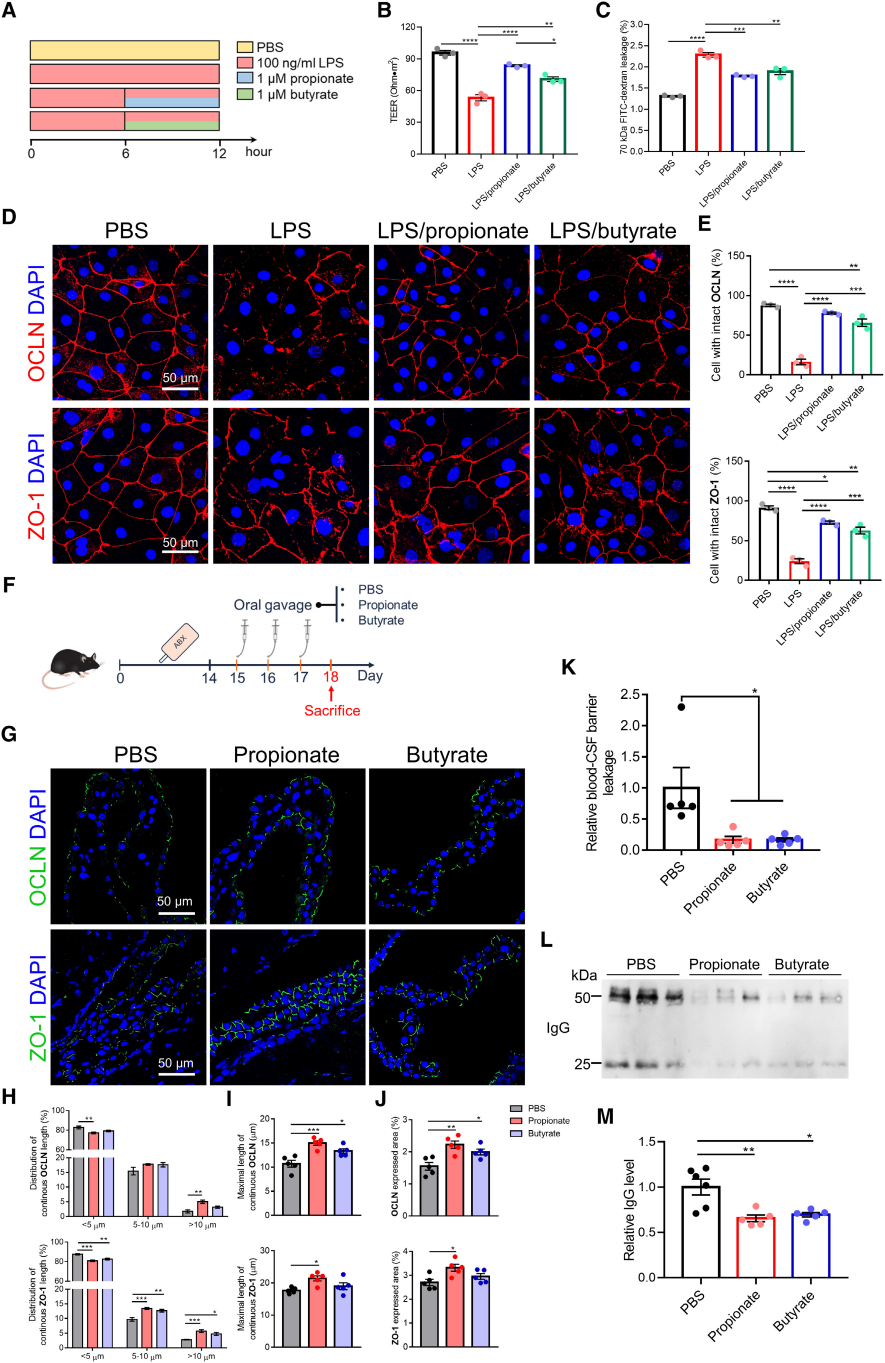
Effect of SCFAs on blood‐CSF barrier integrity *in vitro* and *in vivo* ASchematic representation of the experimental conditions.B, CTEER (B) and assessment of the 70 kDa FITC‐dextran paracellular permeability (C) of primary CPE cells treated as showed in (A) (*n* = 3, technical duplicates).DRepresentative images of immunostainings for ZO‐1 and OCLN in primary CPE cells following treatment for 6 h with 1 μM sodium propionate or 1 μM sodium butyrate, with 100 ng/ml LPS stimulation. Scale bar: 50 μm.EThe percentage of cells with intact TJ immunostainings is displayed (D) (*n* = 3, technical duplicates).FSchematic representation of the experimental groups.GRepresentative images of immunostainings for ZO‐1 and OCLN in choroid plexus of sodium propionate or sodium butyrate‐treated mice. Scale bar: 50 μm.H–JQuantification of continuous length (H), maximal length (I), and expressed area (J) of TJ immunostainings in (G) (*n* = 5, biological replicates).KAssessment of the blood‐CSF barrier permeability to 4 kDa FITC‐dextran (*n* = 5).LRepresentative western blot showing the IgG levels in CSF of sodium propionate or sodium butyrate‐treated mice with disrupted gut microbiota via antibiotics treatment.MQuantitative analysis of IgG in CSF via western blot in (L) (*n* = 5–6, biological replicates). Data information: Bars represent mean ± SEM. Statistics were performed with one‐way ANOVA Bonferroni's *post hoc* test for multiple comparisons. *p < 0.05, **p < 0.01, ***p < 0.001, ****p < 0.0001. CPE, choroid plexus epithelial; CSF, cerebrospinal fluid; LPS, lipopolysaccharides; SCFAs, short‐chain fatty acids; TEER, trans‐epithelial electrical resistance; TJ, tight junction. Source data are available online for this figure.

**Figure EV4:**
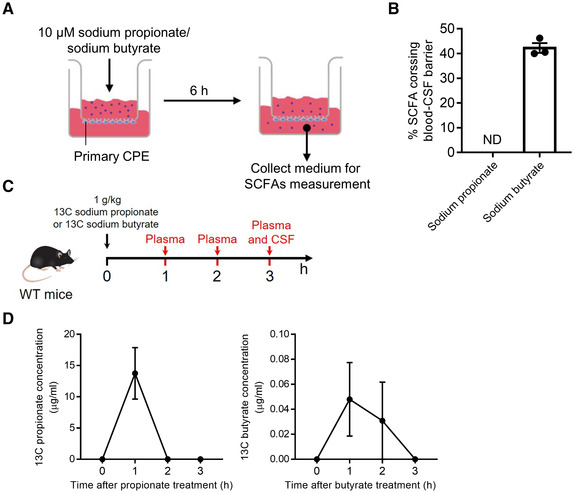
Distribution of SCFAs in *in vitro* and *in vivo* ASchematic representation of the *in vitro* experimental conditions.BThe percentage of SCFAs crossing the blood‐CSF barrier *in vitro* (*n* = 3, technical replicates).CSchematic representation of the *in vivo* experimental conditions.DThe concentrations of 13C sodium propionate (left) and 13C sodium butyrate (right) in plasma (*n* = 5, biological replicates). Data information: Bars represent mean ± SEM. ND, not detected; SCFAs, short‐chain fatty acids. Source data are available online for this figure.

To further investigate the direct effect of SCFAs on the blood‐CSF barrier integrity, we treated primary CPE cultures with sodium propionate or sodium butyrate at 0.1, 1 and 10 μM for 6 h. The CPE cells were exposed to LPS (100 ng/ml) for 6 h to induce a disrupted blood‐CSF barrier as we showed in our previous study (Xie *et al*, 2021), and this was followed by treatment with sodium propionate or sodium butyrate for another 6 h (Fig 4A; Appendix Fig S4A). To avoid potential CPE cell death induced by LPS, we treated primary CPE cells with LPS (50, 100, and 500 ng/ml, respectively) for 12 h and measured cell viability by performing MTT assay and TUNEL staining and this showed no significant increase in cell death even at the highest LPS dose (Appendix Fig S5A and B). The barrier tightness of the CPE cell layer was studied by measuring both trans‐epithelial electrical resistance (TEER; Fig 4B; Appendix Fig S4B) and paracellular permeability to 70 kDa FITC‐conjugated dextran tracer (Fig 4C; Appendix Fig S4B). Treatment of primary CPE cells for 6 h with propionate (0.1, 1 and 10 μM) or butyrate (1 μM) significantly attenuated the LPS‐induced loss of barrier integrity (Fig 4B and C; Appendix Fig S4B). A dose‐dependent effect was observed in propionate but not butyrate upon the response of CPE cells to LPS stimulation and 1 μM appeared to show the best barrier recovery effect in the case of butyrate (Appendix Fig S4B). It has been proposed that the physiological circulating concentrations of propionate and butyrate are approximately 1 μM at rest (Hoyles *et al*, 2018). Paracellular permeability and TEER are largely dependent upon the integrity of inter‐epithelial TJs (Redzic, 2011). To assess this, we examined the subcellular distribution of the key TJs OCLN and ZO‐1 following treatment with sodium propionate and/or LPS. Exposure of primary CPE cells to LPS caused a remarkable disruption in the subcellular localization of both OCLN and ZO‐1, characterized by a loss of peri‐membrane immunoreactivity (Fig 4D and E). Notably, these LPS effects were substantially reduced by subsequent treatment with either sodium propionate or sodium butyrate, although the butyrate treatment showed a lesser ability to improve the localization of TJs (Fig 4D and E). However, this was not reflected by a change in gene expression levels of different TJ genes, namely *Ocln*, *Zo1*, *Cldn1*, and *Cdh1* (Appendix Fig S6A and B). Additionally, we found that approximately 40% sodium butyrate crossed the *in vitro* blood‐CSF barrier from the basal to the apical side (Fig EV4A and B). Unfortunately, sodium propionate was undetectable, even in the basal compartment, due to the low limit of detection (LOD) which was 5–7 μM.

To ensure the validity of our findings in primary CPE cell culture, we next examined AB mice given propionate or butyrate for 3 days (Fig 4F). ZO‐1 and OCLN immunostaining in propionate and butyrate‐treated AB mice demonstrated improved blood‐CSF barrier integrity with higher TJ expression and more continuous TJ subcellular localization, compared with PBS‐treated AB mice with a fragmented border and diffuse distribution of TJs (Fig 4G–J). Consistent with the *in vitro* results, propionate resulted in a greater improvement in blood‐CSF barrier integrity than butyrate (Fig 4G–J). In addition, the propionate and butyrate‐treated AB mice exhibited significantly reduced blood‐CSF barrier permeability based on 4 kDa FITC‐dextran and CSF IgG measurements, confirming the improved barrier integrity after propionate and butyrate treatment (Fig 4K–M).

Altogether, these data suggest that especially propionate but also butyrate to a lesser extent, play a vital role in regulating TJ re‐localization, while there are no data supporting an impact of these SCFAs on RNA expression of tested TJ genes.

### SCFAs can bypass the vagus nerve and tighten blood‐CSF barrier *in vivo*

**Figure 5:**
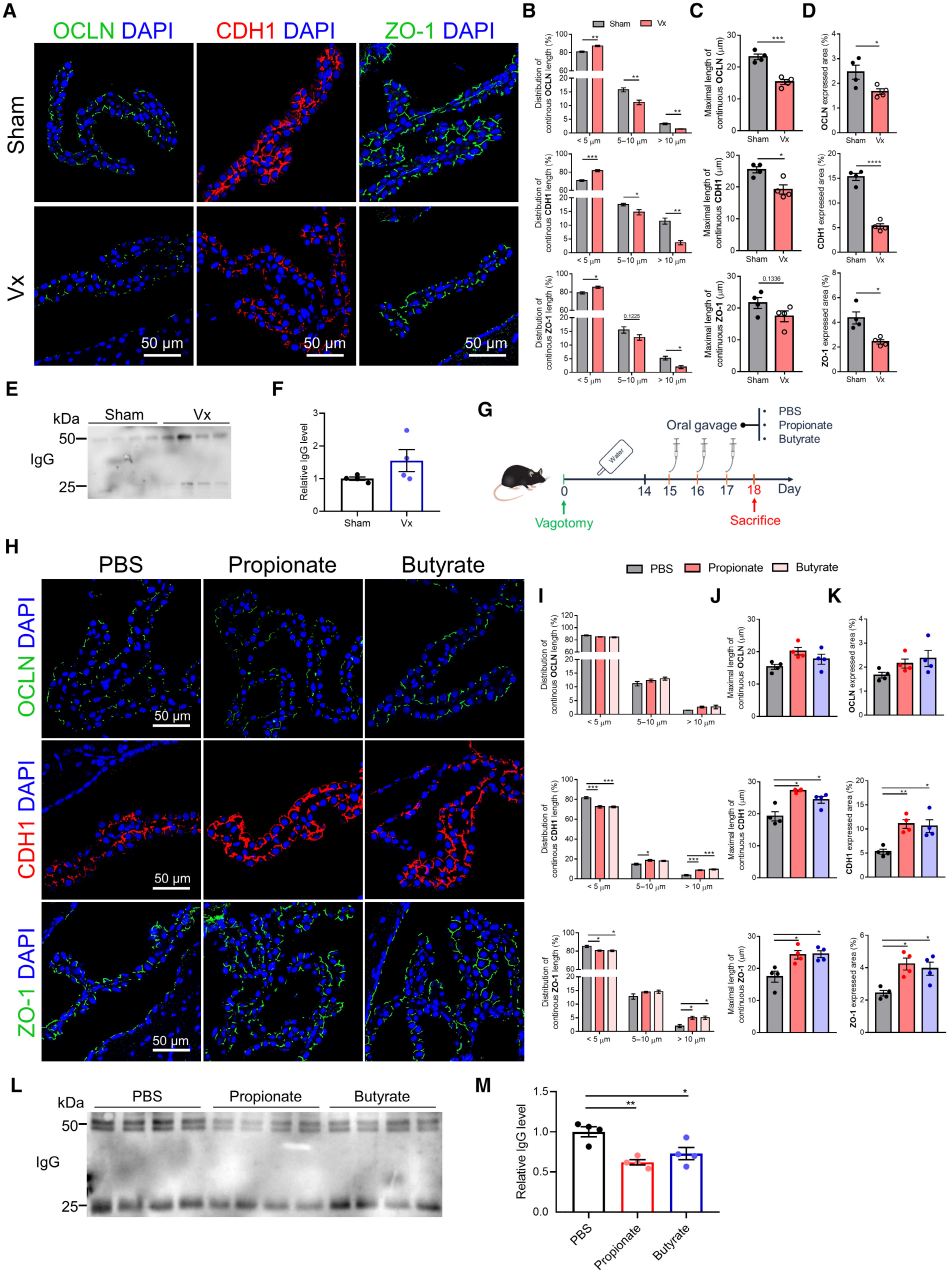
Role of the vagus nerve in regulating the blood‐CSF barrier integrity ARepresentative images of immunostainings for OCLN, CDH1 and ZO‐1 in choroid plexus of sham and vagotomized mice. Scale bar: 50 μm.B–DQuantification of continuous length (B), maximal length (C), and expressed area (D) of TJ immunostainings in (A) (*n* = 4, biological replicates).ERepresentative western blot showing the IgG levels in CSF of Sham and vagotomized mice.FQuantitative analysis of IgG in CSF via western blot in (E) (*n* = 4, biological replicates).GSchematic representation of the experimental groups.HRepresentative images of immunostainings for OCLN, CDH1 and ZO‐1 in choroid plexus of sodium propionate or sodium butyrate‐treated vagotomized mice. Scale bar: 50 μm.I–KQuantification of continuous length (I), maximal length (J), and expressed area (K) of the TJ immunostainings displayed in (H) (*n* = 4, biological replicates).LRepresentative western blot showing the IgG levels in CSF of sodium propionate or sodium butyrate‐treated vagotomized mice.MQuantitative analysis of IgG in CSF via western blot in (L) (*n* = 4, biological replicates). Data information: Bars represent mean ± SEM. Statistics were performed with Unpaired, two‐tailed Student's *t*‐tests (F) or one‐way ANOVA Bonferroni's *post hoc* test for multiple comparisons (other panels). *p < 0.05, **p < 0.01, ***p < 0.001. CSF, cerebrospinal fluid; TJ, tight junction. Source data are available online for this figure.

The vagus nerve, the principal component of the parasympathetic nervous system, has been shown to play an important role in the microbiota (metabolite)‐gut–brain interaction (Bonaz *et al*, 2018). To identify whether SCFAs can mediate the blood‐CSF barrier indirectly through vagus nerve *in vivo*, we first evaluated the blood‐CSF barrier integrity in vagotomized (Vx) mice and we observed that Vx mice displayed a decreased TJ expression and a more severe dyslocalization of TJs at the blood‐CSF barrier compared with AB mice (Fig 5A–D). Consistent with this observation, the CSF IgG level in Vx mice also increased and this level was higher than that in AB mice (Fig 5E and F).

Next, we evaluated Vx mice given sodium propionate or sodium butyrate by oral gavage for 3 days (Fig 5G). The propionate or butyrate treatment increased the TJ expression and improved the localization of TJ proteins in SPF mice with vagotomy (Fig 5H–K). Accordingly, the CSF IgG in SCFA‐treated Vx mice showed a lower level compared with Vx control mice and the butyrate treatment exhibited a significant effect (Fig 5L and M). We next determined whether the gut‐derived SCFAs can be transported to the brain and cross the blood‐CSF barrier. Thereto, WT mice were orally gavaged with 13C‐labeled sodium propionate or sodium butyrate (Fig EV4C). In plasma, we could detect 13C‐labeled sodium propionate and sodium butyrate after 1 and/or 2 h of treatment (Fig EV4D). In CSF, neither 13C‐labeled sodium propionate nor sodium butyrate was detectable, which might either be due to the absence of transport *in vivo* or due to limited CSF volume (~ 7 μl per mouse) and LOD (propionate: 5–7 μM; butyrate: 0.05 μM).

Altogether, these results suggest that the vagus nerve is one of the important pathways for the communication between the gut and the choroid plexus and hence is influential in maintaining the integrity of the blood‐CSF barrier and indicate the potential of SCFAs to directly mediate gut microbiota–brain barrier interactions.

### SCFAs reduce loss of blood‐CSF barrier integrity and AD pathology in *App* ^*NL‐G‐F*^ mice

**Figure 6:**
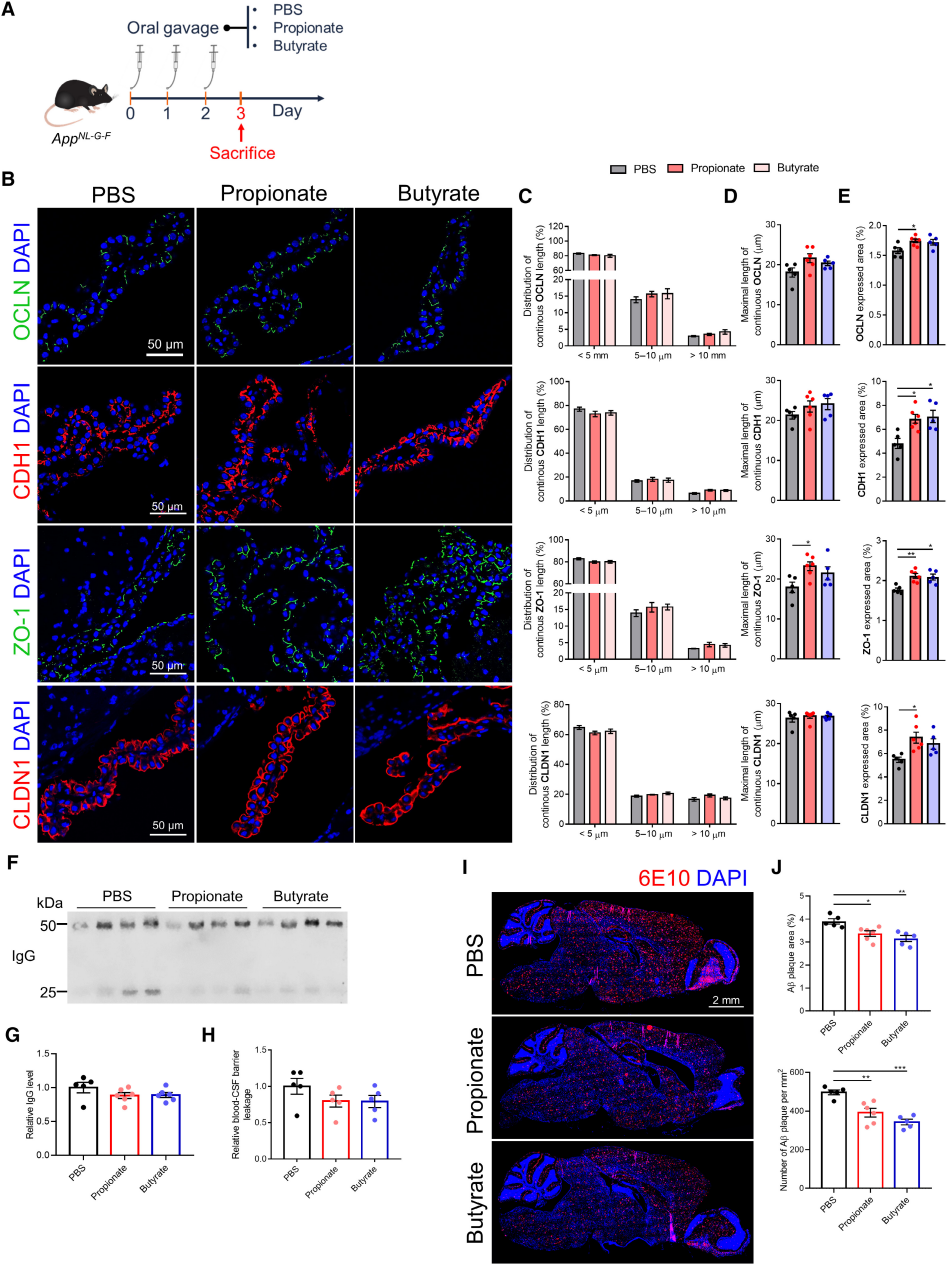
Effects of SCFAs on blood‐CSF barrier integrity and Aβ plaques in *App* ^*NL‐G‐F*^ mice ASchematic representation of the experimental groups.BRepresentative images of immunostainings for OCLN, CDH1, ZO‐1, and CLDN1 in choroid plexus of sodium propionate or sodium butyrate‐treated *App* ^*NL‐G‐F*^ mice. Scale bar: 50 μm.C–EQuantification of continuous length (C), maximal length (D), and expressed area (E) of the TJ immunostainings displayed in (B) (*n* = 5–6, biological replicates).FRepresentative western blot showing the IgG levels in CSF of sodium propionate or sodium butyrate‐treated *App* ^*NL‐G‐F*^ mice.GQuantitative analysis of IgG in CSF via western blot displayed in (F) (*n* = 5–6, biological replicates).HAssessment of the blood‐CSF barrier permeability to 4 kDa FITC‐dextran (*n* = 5, biological replicates).IRepresentative images of 6E10 staining in brain of sodium propionate or sodium butyrate‐treated *App* ^*NL‐G‐F*^ mice. Scale bar: 1 mm.JQuantification of Aβ plaque area and number in whole sagittal section of the brain (*n* = 3 mice; one section per mouse). Data information: Bars represent mean ± SEM. Statistics were performed with one‐way ANOVA Bonferroni's *post hoc* test for multiple comparisons. *p < 0.05, **p < 0.01, ***p < 0.001. CSF, cerebrospinal fluid; SCFAs, short‐chain fatty acids. Source data are available online for this figure.

Numerous studies suggest that disruption of brain barriers is likely involved in the pathogenesis of several neurological diseases, including AD (Ueno *et al*, 2016). In addition, SCFAs, as important mediators of gut–brain interactions, have been implicated in AD development and progression, but it is not well‐known which SCFAs mechanism(s) are important in AD (Qian *et al*, 2022). Based on our results that recognize SCFAs as the key mediators of the gut microbiota's effect on blood‐CSF barrier integrity, we hypothesized that SCFAs might revert AD pathology by tightening the brain barrier. Here, we used *App* ^*NL‐G‐F*^ mice, an AD mouse model in which we previously observed increased blood‐CSF barrier permeability (Xie *et al*, 2021) and a decreased ratio of SCFA‐generating bacteria in feces has been reported (Kaur *et al*, 2020). In agreement with the latter, we observed lower levels of propionate and butyrate in the feces of *App* ^*NL‐G‐F*^ mice compared with their wild‐type counterparts (Appendix Fig S7). Next, we evaluated the changes in BBB and blood‐CSF barrier in *App* ^*NL‐G‐F*^ mice given sodium propionate or sodium butyrate by oral gavage for 3 days (Fig 6A). Compared with *App* ^*NL‐G‐F*^ control mice, propionate and butyrate‐treated *App* ^*NL‐G‐F*^ mice showed significantly increased TJ expression and slightly improved TJ subcellular localization, determined for OCLN, CDH1, ZO‐1, and CLDN1 via quantification of “expressed area” and “distribution of continuous TJ protein length and the maximal length of continuous TJ protein” on immunostainings, respectively (Fig 6B–E). In the case of BBB, both expression and localization of the TJs were only slightly improved (Appendix Fig S8A–D). In addition, butyrate and propionate treatment showed a similar effect on IgG and 4 kDa FITC‐dextran leakage, and both only caused a reduced trend rather than significant changes at the blood‐CSF barrier (Fig 6F–H). However, the decreasing trend of 4 kDa FITC‐dextran leakage of BBB was only observed in the propionate treatment (Appendix Fig S8E).

**Figure 7:**
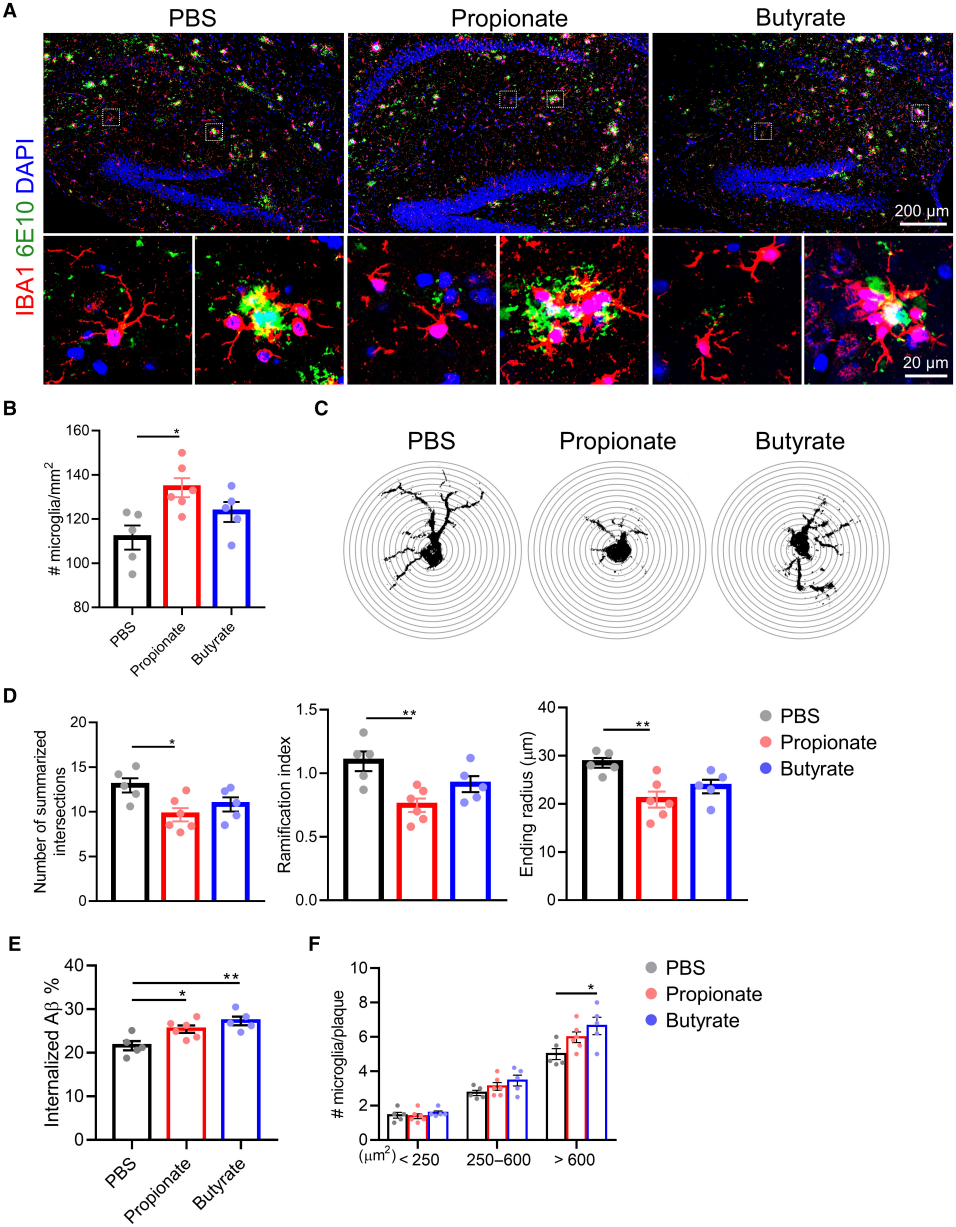
Effects of SCFAs on microglial proliferation and activation in *App* ^*NL‐G‐F*^ mice ARepresentative images of immunostainings for IBA1 and 6E10. Scale bar: 200 μm (up) and 20 μm (down).BThe number of IBA1^+^ microglia in hippocampus (*n* = 5–6, biological replicates).CRepresentative image for Sholl analysis of non‐Aβ associated microglia in the hippocampus from the IBA1‐stained image in (A). The interval of the concentric circles is 2 μm.DQuantification of summarized intersects, ramification index, and ending radius by Sholl analysis from immunostained IBA1 signals (*n* = 5–6, biological replicates).EQuantification of the percentage of overlay area of microglia and Aβ plaque (*n* = 5–6 mice; 5 microglia per mouse).FPlaques were divided into small (< 250 μm^2^), medium (250–600 μm^2^), and large (> 600 μm^2^), and the number of microglia per plaque was quantified (*n* = 5–6 mice; 3–5 plaques per mouse). Data information: Bars represent mean ± SEM. Statistics were performed with one‐way ANOVA Bonferroni's *post hoc* test for multiple comparisons.*p < 0.05, **p < 0.01. SCFAs, short‐chain fatty acids. Source data are available online for this figure.

**Figure EV5:**
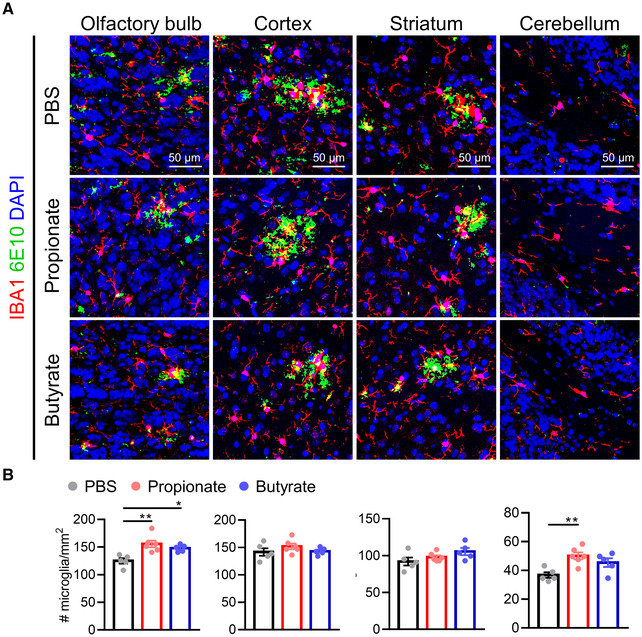
Effects of SCFAs on microglial proliferation in *App* ^*NL‐G‐F*^ mice ARepresentative images of immunostainings for IBA1 and 6E10 in olfactory bulb, cortex, striatum and cerebellum. Scale bar: 50 μm.BThe number of IBA1^+^ microglia in different regions of the brain (*n* = 5–6, biological replicates). Data information: Bars represent mean ± SEM. Statistics were performed with one‐way ANOVA Bonferroni's *post hoc* test for multiple comparisons. *p < 0.05, **p < 0.01. SCFAs, short‐chain fatty acids. Source data are available online for this figure.

Microglia are the most prominent immune cells of the CNS, and they are known to play an important role in the functionality of the BBB and in β‐amyloid (Aβ) clearance (Haruwaka *et al*, 2019; Tejera *et al*, 2019). First, we investigated whether the barrier‐restoring effect of SCFAs could inhibit Aβ aggregation. Strikingly, both propionate and butyrate treatments resulted in a significant decrease of Aβ accumulation in the brain of *App* ^*NL‐G‐F*^ mice, which was reflected in both total Aβ plaque area and the number of Aβ plaques (Fig 6I and J). Less tight barriers may increase infiltration of peripheral factors (e.g., cytokines and immune cells) into the CNS and consequently induce and/or aggravate neuroinflammation (Takata *et al*, 2021). Next, we evaluated whether the barrier‐restoring effect of SCFAs would be beneficial in enhancing the ability of microglia to phagocytose Aβ. A significantly increased number of microglia was observed in the olfactory bulb of propionate and butyrate‐treated *App* ^*NL‐G‐F*^ mice, while only propionate treatment induced a pronounced increase in the number of microglia in the hippocampus and cerebellum. In the cortex and striatum, the number of microglia did not show a difference after SCFAs treatment (Figs 7A and B, and EV5A and B). The detailed Sholl analysis of non‐Aβ plaque‐associated microglia showed that the sum of intersections, ramification index, and ending radius decreased significantly in propionate‐treated *App* ^*NL‐G‐F*^ mice (Fig 7C and D), indicating a more activated microglial phenotype. Similarly, the sodium butyrate treatment showed the same trends in microglial proliferation and activation but the changes were not significant (Fig 7A–D). Additionally, we also observed a pronounced increase in microglial internalized Aβ in both propionate and butyrate‐treated *App* ^*NL‐G‐F*^ mice, but only butyrate treatment showed a significant increase in the number of microglia adjacent to the large plaques (> 600 μm^2^) in *App* ^*NL‐G‐F*^ mice (Fig 7E and F). However, when administering the same doses of propionate and butyrate in WT mice, we did not observe a difference in microglial proliferation or activation (Appendix Fig S9A–D). These results indicate that under physiological conditions, supplementation of SCFAs has no pronounced effect on microglial activation and proliferation; however, under pathological conditions, SCFA treatment may promote a reduction of Aβ burden in the brain by increasing the number of microglia and inducing microglial activation. These may benefit from the barrier‐restoring effects of SCFAs, which increase the limit of infiltration of neuroinflammation‐inducing peripheral factors, thereby preventing microglial hyperactivation and shifting toward a functional phenotype.

Of note, microglial activation may lead to the conversion of resting astrocytes to reactive astrocytes and neuronal dysfunction (Leng & Edison, 2020). First, we examined the effects of propionate and butyrate on the proliferation and activation of astrocytes in WT and *App* ^*NL‐G‐F*^ mice (Appendices Figs S10A and B, and S11A and B). GFAP immunostaining showed no significant changes in astrocyte morphology nor number in WT and *App* ^*NL‐G‐F*^ mice after propionate and butyrate treatments (Appendices Fig S10A and B, and S11A and B). Next, we analyzed the effects of propionate and butyrate on synaptic properties in *App* ^*NL‐G‐F*^ mice (Appendix Fig S12A–D). High‐resolution images of co‐immunostaining of presynaptic protein synaptophysin (SYP) and postsynaptic density protein 95 (PSD‐95) showed a similar number of double‐positive synaptic puncta in the area of the cortex and hippocampus of SCFA‐treated *App* ^*NL‐G‐F*^ mice compared with *App* ^*NL‐G‐F*^ controls (Appendix Fig S12A and B). Moreover, co‐immunostaining of IBA1 and PSD‐95 did not show excessive synaptic pruning by microglia, and even a downregulation trend was observed (Appendix Fig S12C and D).

## Discussion

The brain is protected from invading substances by tight barriers, including the blood–brain, and the blood‐CSF barrier. These barriers control the passage of molecules between the blood and the brain parenchyma and assure a balanced and well‐controlled micro‐environment in the CNS (Kadry *et al*, 2020). In our study, we found that the lack of normal gut microbiota in mice, modeled via either germ‐free (GF) mice or treatment with antibiotics (AB), is not only associated with increased BBB permeability but also with increased blood‐CSF barrier permeability. Strikingly, the compromised BBB and blood‐CSF barrier can be rescued by transplantation of normal fecal microbiota or supplementation of SCFA, indicating great plasticity of the gut and blood‐CSF barrier connection.

Tight junctions play a major role in the maintenance of the blood‐CSF barrier (Solar *et al*, 2020). Although the blood‐CSF barrier is already morphologically and functionally mature early on in embryonic development (Johansson *et al*, 2006), the increased blood‐CSF barrier permeability in AB mice indicates that a continuous input from complex microbiota is necessary to maintain the blood‐CSF barrier. Here, we observed the delocalization of TJ proteins of ZO‐1, OCLN, and CDH1 proteins in mice with the absence of gut microbiota, namely AB and GF mice. However, the localization of CLDN‐1 was not disturbed in AB mice, indicating that CLDN‐1 may not be susceptible to changes in gut microbiota after complete maturation. The disorganized TJs were not reflected in the gene expression of the corresponding genes, indicating that gut microbiota mediate blood‐CSF barrier integrity by regulating localization of TJs directly rather than altering their corresponding gene expression. Similar effects on the blood‐CSF barrier integrity were observed in GF mice with constitutive depletion of gut microbiota. The latter is in agreement with a recent publication that compared the blood‐CSF barrier of GF with conventional mice which revealed a decrease in the ZO‐1 network organization in GF mice (Knox *et al*, 2023). Altogether this indicates the important role of complex microbiota in blood‐CSF barrier maturation.

An important question is how commensal microbes in the gut control this blood‐CSF barrier formation and maintenance. Previous research has shown that metabolites of gut microbiota, especially short‐chain fatty acids (SCFAs), have essential signaling functions which can modulate BBB integrity and brain function (Parker *et al*, 2020). Recent studies have identified that the gut microbiota‐lacking mice have significant changes in metabolomic signatures of feces and/or serum (Theriot *et al*, 2014; Lai *et al*, 2021). SCFAs are the main metabolites produced by bacterial fermentation of dietary fiber in the gastrointestinal tract and have been shown to regulate BBB maturation by increasing the expression of TJs and decreasing BBB permeability (Braniste *et al*, 2014; Hoyles *et al*, 2018). In our study, treatment with the SCFAs propionate and butyrate *in vitro* and *in vivo* prevented both the disorganization of TJs at the blood‐CSF barrier and the increase of the barrier's permeability induced by an inflammatory trigger. In general, butyrate treatment showed a weaker ability to improve the localization of TJs relative to propionate, which may, among others, be due to differences in their stability in the blood, the expression of corresponding receptors on the CPE cells, or the uptake efficiency by CPE cells. However, which SCFA receptors are expressed on CPE cells is unclear and requires further investigation. Our experiments in which primary CPE cell cultures were treated with SCFAs suggest that this is not due to major changes in TJ gene expression, but either due to TJ subcellular localization which was heavily affected upon treatment.

SCFAs have been shown to play a vital role in directly or indirectly mediating microbiota‐gut–brain interactions. Mechanistic studies have identified four principal signaling pathways involved in this interaction, including immune, endocrine, neural, and humoral routes (Dalile *et al*, 2019). Hoyles *et al* (2018) have demonstrated a direct action of SCFAs on BBB function through the circulation and identified that free fatty acid receptor 3 (FFAR3) in the endothelium may be the predominant receptor of SCFAs mediating the protective effects. In the choroid plexus, however, a low *FFAR3* gene expression was detected in adult humans (Rouillard *et al*, 2016) and no expression of *Ffar3* was found in our RNA‐seq data in mice, indicating that the direct action of SCFAs on the blood‐CSF barrier may not be the dominant mechanism, and other mechanisms are most probably involved. The vagus nerve, containing 80% afferent and 20% efferent fibers and innervating almost all of the digestive tract, is not in direct contact with the gut microbiota or luminal content, but can indirectly sense luminal signals through diffusion across the gastrointestinal barrier of bacterial compounds or metabolites (Bonaz *et al*, 2018). Interestingly, Yang *et al* (2018) have shown that vagus nerve stimulation can affect the integrity of the BBB. Therefore, we hypothesized that the vagus nerve might play a role in regulating the integrity of the blood‐CSF barrier by SCFAs. In line with this assumption, celiac vagotomy was found to disrupt TJs and increase permeabilization. Notably, the destructive effect of celiac vagotomy is stronger than that of gut microbiota depletion by antibiotics treatment, indicating the vagus nerve is one of the important pathways for the transmission of a gut microbiota‐derived signal to the choroid plexus and hence is influential in maintaining the integrity of the blood‐CSF barrier. It is important to note that the effect of vagotomy on the brain barriers may be partly due to its effect on the gut microbiota, as previous studies have shown that vagotomy can alter the composition of the gastrointestinal microbiota (Lee *et al*, 2020). However, SCFA supplementation to Vx mice still induced blood‐CSF barrier‐restoring effects by improving the localization of TJs, indicating SCFAs can bypass the vagus nerve and affect the blood‐CSF barrier functions via other routes. These routes may include the direct crossing of the brain barrier and indirect modulation of neuroinflammation through the activation of FFARs on the BBB endothelium (Hoyles *et al*, 2018; Silva *et al*, 2020). In our study, we found that SCFAs can enter the bloodstream upon oral administration. Although we were unable to determine based on our current methods whether SCFAs could also cross the blood‐CSF barrier and enter the CSF, our *in vitro* model showed that sodium butyrate can cross the tightly connected CPE cells from the basal to the apical side. Thus, we speculate that gut‐derived SCFAs can reach the brain and cross the blood‐CSF barrier. However, further validation by more sensitive methods is required to be sure whether or not SCFAs enter the CSF. Furthermore, future studies are warranted to determine the contribution of the vagus nerve and other potential pathways in facilitating the effect of SCFAs on blood‐CSF barrier integrity.

Increasing evidence suggests that dysfunction of the BBB may be involved in AD and other neurodegenerative diseases (Varatharaj & Galea, 2017; Sweeney *et al*, 2018). Our recent study has also indicated that the AD mice have an increased blood‐CSF barrier permeability and disrupted TJs compared with wild‐type mice (Xie *et al*, 2021). Additionally, AD mice also exhibit a decreased number of SCFA‐generating bacteria in the feces and a reduction of SCFAs in plasma (Zhang *et al*, 2017; Kaur *et al*, 2020), which is in accordance with our observation that AD mice have significantly reduced fecal SCFAs levels. Several mechanisms of action of SCFAs on AD pathology have been proposed, including metabolic distribution, specific receptors, and signaling pathways, but it remains largely unknown which SCFAs mechanism(s) are important in AD (Qian *et al*, 2022). In our study, we observed that treatment with SCFAs propionate and butyrate improved the integrity of the BBB and blood‐CSF barrier and decreased their permeability in *App* ^*NL‐G‐F*^ mice. However, the rescue effect of both tested SCFAs on the barrier integrity is weaker in the *App* ^*NL‐G‐F*^ mice compared with WT mice, which may be due to the presence of AD pathology (e.g., Aβ plaques) as this will be a continuous trigger inducing barrier integrity disruption. This slight improvement of the barrier integrity in the *App* ^*NL‐G‐F*^ mice upon SCFA treatment was however associated with a significant reduction in Aβ accumulation in the brain, which is in concordance with previous reports, showing that administration of sodium butyrate can decrease brain Aβ levels and improve cognitive memory performance in a transgenic mouse model of AD (Fernando *et al*, 2020). Nevertheless, we cannot exclude the possibility that SCFAs may affect microglial phenotype and Aβ pathology through other mechanisms than brain barrier tightening. In this regard, both propionate and butyrate were shown to induce microglial activation resulting in reduced Aβ accumulation by increasing Aβ phagocytosis, clearance, and degradation (Solito & Sastre, 2012). Importantly, we could not show the presence of the administered SCFAs in the brain, which is needed to exert direct effects on the microglia, while we did show that SCFA *in vitro* strengthens the blood‐CSF barrier TJs directly. However, SCFAs may also indirectly affect microglial functions by mediating inflammation, as butyrate is a strong anti‐inflammatory mediator, while other SCFAs, such as propionate, enhance the inflammatory responses (Huuskonen *et al*, 2004). The inflammatory effects of SCFAs may further impact microglial activation and then microglial Aβ phagocytosis. In this study, we found that SCFA treatment increased microglial Aβ internalization and the number of microglia adjacent to large Aβ plaques in *App* ^*NL‐G‐F*^ mice. Notably, propionate induces higher levels of microglial activation than butyrate, and this state of hyperactivation may reduce their Aβ phagocytic capacity, as we demonstrated in our previous study (Xie *et al*, 2021). In addition to the inflammatory effects, SCFAs can also regulate microglia maturation and function, possibly through upregulation of the ApoE‐TREM2 pathway and increasing histone acetylation (Erny *et al*, 2015, 2021; Colombo *et al*, 2021; Bauer *et al*, 2022); thereby affecting the ability of microglia in the internalization and degradation of Aβ (Lee & Landreth, 2010). The differences in propionate and butyrate in affecting these pathways may also contribute to their differential microglial Aβ phagocytosis. In contrast to our results, a recent study reported that treatment with SCFA mixtures (sodium acetate, sodium propionate, and sodium butyrate) increased the Aβ plaque load in GF AD mice to levels of normal SPF mice and even further exacerbate Aβ burden in AD mice (Colombo *et al*, 2021). The varied outcomes could be due to the different experimental setups, such as mouse model, age at SCFA treatment, SCFA dosage, and treatment route. For example, excessive propionate intake in healthy humans has been reported to increase the risk for AD (Killingsworth *et al*, 2020). In our study, we observed that SCFA treatment increased the number of microglia and induced microglial activation in the hippocampus of *App* ^*NL‐G‐F*^ mice but not WT mice, indicating that under physiological conditions, supplementation of SCFAs has no pronounced effect on microglial proliferation and activation; however, under pathological condition, SCFA treatment may promote a reduction of Aβ burden in the brain by increasing the number of microglia and inducing microglial activation. The microglia may benefit from the barrier‐restoring effects of SCFAs, which limit the leakage of neuroinflammation‐inducing peripheral factors into the brain, thereby preventing microglial hyperactivation and the maintenance of their functionality. Thus, further studies are needed to evaluate the beneficial potential of individual SCFAs on AD and develop the rationale for personalized treatment strategies. In addition, previous studies have shown that microglial activation may lead to the conversion of resting astrocytes to reactive astrocytes and neuronal dysfunction such as excessive synaptic pruning (Azevedo *et al*, 2013; Litvinchuk *et al*, 2018; Leng & Edison, 2020), but we did not observe changes in astrocyte proliferation and activation, nor synaptic loss in SCFA‐treated *App* ^*NL‐G‐F*^ mice. These results indicate that SCFA‐induced increased phagocytic activity and proliferation of the microglia have no effect on astrocyte activation and synaptic pruning.

In summary, our study reveals a significant new aspect of the modulatory effects of gut microbiota and their metabolites, more specifically SCFAs, on the epithelial cells that are essential for the blood‐CSF barrier integrity. In addition, the compromised BBB and blood‐CSF barrier in AD mice can be rescued by supplementation of SCFAs and this treatment can suppress the Aβ pathology. Our findings add to the knowledge of the contribution of gut microbiota and SCFAs to the integrity of the CNS barriers and to the development of neurological disorders such as AD. Importantly, this knowledge sets the stage for future investigation of the influence of gut microbiota or its metabolites on brain barrier structure and as a target for therapeutic interventions in neurological diseases.

## Materials and Methods

### Animals

Germ‐free (GF) and specific pathogen‐free (SPF) C57BL/6J female mice (7–9 weeks old) and *App* ^*NL‐G‐F*^ mice (56–58 weeks old) were used. GF mice were housed in positive‐pressure flexible film isolators (North Kent Plastics) in GF facility at Ghent University. Mice were housed in individually ventilated cages in an SPF animal facility at the VIB‐UGent Center for Inflammation Research. The animals were randomly assigned to different groups when applicable. All animal studies were conducted in compliance with governmental and EU guidelines for the care and use of laboratory animals and were approved by the ethical committee of the Faculty of Sciences, Ghent University, Belgium.

### Depletion and recolonization of the microbiota

For substantial depletion of the microbiota, SPF mice were provided drinking water containing 0.2 mg/ml ciprofloxacin (Sigma‐Aldrich), 1 mg/ml ampicillin (Sigma‐Aldrich), 1 mg/ml metronidazole (Sigma‐Aldrich), and 0.5 mg/ml vancomycin (Duchefa Biochemie) for 2 weeks *ad libitum* as described previously (O'Connor *et al*, 2021). Antibiotics were renewed every other day. 0.05 g of feces (2–3 pellets) was solubilized in 1 ml PBS, and the resulting inoculum was plated on BHI plates to track the success of the treatment. For recolonization, GF and antibiotics‐treated mice received fecal matter from the SPF mice through two gavages at Day 0 and Day 2 (once a day) and then were left for 2 weeks before being sacrificed. A group of SPF mice was gavaged with sterile PBS as control.

### Celiac vagotomy

To perform celiac vagotomy, the mice were incised at the central abdomen to expose the front wall of the esophagus and then the left and right vagal trunks were subdiaphragmatically transected. The nonvagotomized (sham) group underwent an incision of the central abdomen without vagotomy. We confirmed the success of vagotomy by anti‐ChAT immunostaining on the dorsal motor nucleus of the vagus (DMV). As shown in Appendix Fig S13, vagotomy induced a significant reduction in the number of ChAT‐positive neurons at both sides of DMV when compared to the sham DMV. The samples from mice with unsuccessful vagotomy were excluded.

### SCFA treatment

Mice in blood‐CSF barrier crossing experiments were gavaged for 3 days with 13C‐labeled sodium propionate (Cambridge Isotope Laboratories) and sodium butyrate (Cambridge Isotope Laboratories; 1 g/kg body weight [BW] per day) before sacrifice. Mice in other experiments were administered the same doses of sodium propionate (Sigma‐Aldrich) or sodium butyrate (Sigma‐Aldrich). The control groups were gavaged with sterile PBS.

*In vitro* cell cultures were treated with sodium propionate or sodium butyrate (0.1, 1, or 10 μM) for 6 h prior to analysis of barrier function.

### Bulk RNA‐seq and downstream analysis

Total RNA from choroid plexus tissue was extracted using Aurum total RNA kit (Bio‐Rad). The RNA‐seq analysis was performed on an Illumina NovaSeq 6000 instrument. The sequencing of the SPFR and GFR samples was performed separately. Two technical replicates of each of the four groups (SPF, GF, AB, and ABR) of the first sequencing run were taken along again for resequencing to determine that there was no technical batch effect between sequencing runs. Preprocessing of the RNA‐seq data was performed by Trimmomatic v0.39 (Bolger *et al*, 2014) and quality control by FastQC v0.11.8 (https://www.bioinformatics.babraham.ac.uk/projects/fastqc/). Mapping to the reference mouse genome was accomplished by STAR v2.7.3a, BAM files were created with Samtools v1.9, and HTSeqCount v0.11.2 was used for counting (Dobin *et al*, 2013; Anders *et al*, 2015). Limma v3.42.2 was used to normalize the data (Ritchie *et al*, 2015). Genes that did not meet the requirement of a count per million (cpm) value larger than one in at least four samples were filtered out. One GFR sample was determined to be an outlier based on PCA ellipse analysis (outside 2 z‐scores) and was removed from the downstream analysis. This resulted in an expression table containing 14,251 genes and 30 samples for the choroid plexus dataset. EdgeR v3.28.1 was utilized to perform differential expression (DE) analysis (Robinson *et al*, 2010). Benjamini–Hochberg correction was used to adjust the *P*‐values for multiple testing. To be labeled as a DE gene, a gene needed to have an adjusted *P*‐value < 0.05 and a log_2_ratio > 1 or < −1. The R package pheatmap v1.0.12 (https://CRAN.R‐project.org/package=pheatmap) was used to create a heatmap of all the DE genes in the choroid plexus between AB and SPF mice. The displayed gene expression was log_2_ normalized. The mean expression value per gene over all samples was calculated and then subtracted from each sample's particular gene expression value to scale the expression values. Three extra annotation columns were added to the heatmap to indicate which genes were core enrichment genes for certain GO categories. Membrane is a combination of GO categories integral component of membrane (GO:0016021) and intrinsic component of membrane (GO:0031224). Cell junction is its own category (GO:0030054), and cell adhesion refers to cell–cell adhesion via plasma‐membrane adhesion molecules (GO:0098742).

Gene set enrichment analysis (GSEA) was performed using the pre‐ranked DE gene list and full gene distribution in the ClusterProfiler R package v3.14.3 (Yu *et al*, 2012). All three ontologies (“Biological Pathway,” “Molecular Function,” and “Cellular Compartment”) were included, and a *P*‐value cut‐off of 0.05 was applied. The top 10 significant up and downregulated Gene Ontology (GO) categories (according to *P*‐value) are featured in the dot plot. Subsequently, the relevant top GO categories are highlighted in GSEA plots. The Running Enrichment Score (RES) is plotted on the Y‐axis in the top half of the plot for one gene set (green line) or multiple gene sets (different colors). The RES is calculated by running down the preranked gene list and updating a running‐sum statistic depending on if a gene is in the gene set (increase RES) or not (decrease RES). This score shows whether the gene set is overrepresented at the top or bottom of the preranked list of genes. The barcode lines (black with one gene set or multiple colors with multiple gene sets) in the middle of the plot show where the genes in the gene set are situated in the full preranked gene list. The ranked list metric at the bottom of the plot displays how the ranking metric (logFC) evolves as you move down the preranked gene list. It indicates with which phenotype/condition the GO terms are correlated. The Normalized Enrichment Score (NES) and P‐value for the respective gene sets are shown in an embedded table on the GSEA plot.

Propanoate metabolism genes were determined by running the gseKEGG function of the clusterProfiler package. This indicated an enrichment of the KEGG pathway mmu00640 (propanoate metabolism) gene set based on the full gene distribution between Antibiotics and Control conditions in CP. The R package pheatmap v1.0.12 was used to create a heatmap of the genes that were linked to the KEGG pathway. The displayed gene expression was log_2_ normalized. The mean expression value per gene over all samples was calculated and then subtracted from each sample's particular gene expression value to scale the expression values.

The R package pheatmap v1.0.12 was also used to create a heatmap of all the DEGs in choroid plexus across the four relevant comparisons: AB vs. SPF mice (79), ABR vs. AB mice (0), GF vs. SPF mice (12), and GFR vs. GF mice (191). There are 282 rows in the heatmap representing 259 unique genes. The comparisons are separated from each other with an empty row. Within each comparison, the genes are ordered in descending order according to logFC. The displayed gene expression was log2 normalized. The mean expression value per gene over all samples was calculated and then subtracted from each sample's particular gene expression value to scale the expression values.

### SCFA measurement

Organic extraction of fecal metabolites was conducted using the Ribolyzer homogenizer. To the feces, we added 500 μl of extraction buffer (80% MeOH). The insolubilities were removed by centrifugation (20,000 *g*, 15 min at 4°C). QC samples for feces were created by mixing an aliquot from each sample. Two microliters of each sample were injected into an iHILIC‐equipped UPLC in‐line connected to an OrbiTRAP Fusion Lumos MS. The AcquireX option was used to set up the sequence, with an MS range of 70*–*750 *m*/*z*. For identification, an exclusion list obtained from a blank sample (80% MeOH) run before the quality control (QC), and an inclusion list obtained from QC were used. All samples in each set were run scrambled. The fragmentation was done by collision‐induced dissociation (CID) and high collision dissociation (HCD).

Medium, plasma, and CSF were extracted by adding extraction buffer (80/20 MeOH/water). Medium and plasma were diluted 100‐fold in extraction buffer, and CSF was diluted 10‐fold in extraction buffer. These extracts were left overnight at −80°C, and then insolubilities were removed by centrifugation (20,000 *g*, 10 min at 4°C). Ten microliter of each sample was injected into a Poroshell 120 HILIC‐Z PEEK Column (Agilent InfinityLab) connected to a Vanquish LC System (Thermo Scientific) coupled via heated electrospray ionization to a Q Exactive Orbitrap Focus mass spectrometer (Thermo Scientific). A linear gradient was carried out starting with 90% solvent A (acetonitrile with 5 μM medronic acid) and 10% solvent B (10 mM NH_4_‐formate in milli‐Q water, pH 3.8). From 2 to 12 min, the gradient changed to 60% B. The gradient was kept on 60% B for 3 min and followed by a decrease to 10% B. The chromatography was stopped at 25 min. The flow was kept constant at 0.25 ml/min. The column temperature was kept constant at 25°C. The mass spectrometer operated in full scan (range 70.0000–1,050.0000) and negative mode using a spray voltage of 2.8 kV, capillary temperature of 320°C, sheath gas at 45, auxiliary gas at 10, the latter heated to 260°C. AGC target was set at 3.0 × 10^6^ using a resolution of 70,000. Data collection was performed using the Xcalibur software (Thermo Scientific). The data analyses were performed by integrating the peak areas (El‐Maven—Polly‐Elucidata).

### Quantification of the BBB and blood‐CSF barrier permeability

Blood–brain barrier and blood‐CSF barrier leakage was analyzed as described previously (Vandenbroucke *et al*, 2012). In brief, 4 kDa FITC‐dextran was i.v. injected 15 min before CSF isolation by cisterna magna puncture. For blood‐CSF barrier analysis, 1 μl of CSF was diluted in 99 μl PBS and fluorescence was measured using the FLUOstar Omega reader (λ_ex_/λ_em_ = 485/520 nm). For the analysis of BBB integrity, mice were transcardially perfused with PBS/heparin and brain was isolated. Next, perfused brain samples were incubated in formamide overnight, samples were centrifuged for 15 min at 20,000 *g* in 4°C, and 100 μl supernatant was used for fluorescence measurement using the FLUOstar Omega reader (λ_ex_/λ_em_ = 485/520 nm).

### Primary mouse CPE cells isolation

Primary mouse CPE cells were isolated from P2‐P7 pups as previously described (Balusu *et al*, 2016). Choroid plexus tissue was isolated from the lateral and fourth ventricles, pooled, and digested with pronase (Sigma‐Aldrich) for 7 min. For monolayer cultures, cells were plated on 24‐well plate or Transwell polyester inserts (pore size, 0.4 μm; surface area, 33.6 mm^2^; Corning) coated with laminin (Sigma‐Aldrich). Cells were grown in DMEM/F12 medium supplemented with 10% FBS, 2 mM L‐Glutamine (Gibco), 1% penicillin/streptomycin at 37°C and 5% CO_2_ for 1–2 weeks until their trans‐epithelial electrical resistance (TEER) values reached a plateau.

### *In vitro* barrier function assessments

Paracellular permeability and TEER were measured on 100% confluent cultures. The permeability of epithelial cell monolayers to 70 kDa FITC‐dextran (0.2 mg/ml) was measured as described previously (Hoyles *et al*, 2018); data are presented as the percentage of FITC‐dextran passing through the Transwell. TEER measurements were performed using a Millicell ERS‐2 Voltohmmeter (Millipore, Watford, UK) and are expressed as Ohm•cm^2^.

### RNA extraction and RT–qPCR analysis

Cells were collected and washed once with ice‐cold PBS. After incubating with TRIzol (Invitrogen) for 5 min, the TRIzol/cell lysate was separated into 3 phases by centrifugation at 20,000 *g* in a microcentrifuge at 4°C for 15 min. Total RNA was extracted from the upper aqueous phase using Aurum total RNA kit (Bio‐Rad) according to the manufacturer's instructions. The concentration of total RNA was determined by the Nanodrop‐1000 (Thermo Scientific), and total RNA was reverse‐transcribed into cDNA with SensiFAST™ cDNA Synthesis Kit (Bioline). qPCR was performed on the Roche LightCycler 480 System (Applied Biosystems) using SensiFAST™ SYBR® No‐ROX Kit (Bioline). Results are given as relative expression values normalized to the geometric mean of reference genes, determined using GeNorm. The sequences of the primers are depicted in Appendix Table S1.

### IgG western blotting

Five microlitre CSF was diluted in 15 μl PBS containing a complete protease inhibitor (Thermo Scientific). The samples were denatured in 5× Laemmli buffer, separated by SDS–PAGE gel electrophoresis, and transferred to the nitrocellulose membrane. Following blocking with Odyssey Blocking buffer (LI‐COR Biosciences), the membrane was first incubated with biotinylated goat anti‐mouse IgG (1:2,000; Thermo Scientific) and then with HRP‐conjugated Streptavidin (1:5,000; Thermo Scientific). Protein bands were detected and quantified by WesternBright Quantum HRP substrate (Advansta) in Amersham Imager 600 (GE Healthcare).

### Immunohistochemistry

For immunostainings on brain sections, mice were transcardially perfused with ice‐cold 4% PFA in PBS. Subsequently, brains were carefully separated from the skull and split into two halves (mid‐sagittal). The right hemispheres were embedded in Frozen Section Medium (Thermo Scientific) immediately in cryomolds (Sakura) that were frozen on dry ice and stored at −80°C until further use. The left hemispheres were postfixed overnight with 4% PFA in PBS at 4°C. After dehydration, samples were embedded in paraffin in cryomolds and stored at RT until further use. The brains were cut into 5 μm slices paraffin sections (HM 340 E, Thermo Scientific) or 20 μm cryosections (CryoStar NX70, Thermo Scientific). These brain samples were cut from the sagittal superior sinus toward the border of the cerebral hemisphere. Sections were collected serially for TJ staining when the choroid plexus was initially present and for other staining when the entire hippocampus was initially present (Appendix Fig S14). One section per mouse was analyzed. For immunofluorescence staining, sections were permeabilized in PBS containing 0.3–0.5% Triton X‐100. Following being blocked with goat immunomix (GIM; 5% goat serum, 0.1% BSA, 0.3–0.5% Triton X‐100 in PBS) at RT for 1 h, sections were incubated with primary Abs in GIM at 4°C overnight. After washing with PBS, sections were stained with fluorophore‐conjugated secondary Abs in PBS or PBS containing 0.1% Triton X‐100 at RT for 1–1.5 h. Counterstaining was done with Hoechst reagent (Sigma‐Aldrich, 1:1,000 in PBS). Primary antibody Occludin (cat. no. 33‐1500, Thermo Scientific); E‐cadherin (cat. no. 610181, BD Biosciences); IBA1 (cat. no. 019‐19741, Wako); GFAP (cat. no. ab53554, Abcam); Synaptophysin (cat. no. ab32127); PSD‐95 (cat. no. MA1‐045, Thermo Scientific); and 6E10 (cat. no. 803001, Biolegend) were used on paraffin sections; ZO‐1 (cat. no. 61‐7300, Thermo Scientific) and Claudin‐1 (cat. no. 51‐9000, Thermo Scientific) were used on cryosections; CD31 (cat. no. DIA‐310, Dianova) was used on both paraffin sections and cryosections. For immunostainings on primary cells, cells were fixed with 2% PFA for 20 min on ice. Next, cells were washed three times with PBS and permeabilized with 0.1% Triton X‐100 for 10 min on ice. Samples were washed with blocking buffer (1% BSA in PBS) and incubated with primary Abs (diluted in blocking buffer) for 2 h at RT/overnight at 4°C. After washing with PBS, cells were stained with fluorophore‐conjugated secondary Abs in PBS for 1 h at RT. Next, samples were counterstained with Hoechst and the sections were mounted. A Zeiss LSM780 confocal microscope or Zeiss Axioscan Z.1 was used for imaging.

### Imaging and quantification

For TJ stainings, choroid plexus located in the lateral ventricles was evaluated in all experiments. The imaging was done with the 40× oil objective using confocal microscopy. Images were processed using ImageJ, and the continuous TJ length was quantified with the Ridge Detection plugin for ImageJ. The percentage of TJ expression area was quantified by dividing the area of TJ signal by the area of epithelial nuclei.

For the Aβ plaques staining, imaging of whole brain sections was conducted with 20× objective using Axioscan Z.1. The Aβ plaques were quantified by ImageJ in Analyze Particles with a minimal plaque area of 100 μm^2^ cutoff.

For synaptic puncta colocalization analysis, brain paraffin sections were co‐immunostained with the anti‐SYP and anti‐PSD‐95 antibodies and imaged with the 63× oil objective with the 3× zoom using confocal microscopy. Images were processed using ImageJ, and the number of colocalized puncta was quantified with the Synapse Counter plugin for ImageJ (Dzyubenko *et al*, 2016).

### Statistical analysis

All data were assumed to be normally distributed for statistical analysis. Statistical significance was determined using two‐tailed Student's *t*‐tests to compare two groups or, for multiple comparison analysis, one‐way ANOVA followed by Bonferroni's *post hoc* test (GraphPad Prism 8). *p* < 0.05 was considered statistically significant. Values were expressed as means ± SEM.

EdgeR v3.28.1 was used to carry out differential expression (DE) analysis on the Bulk RNA sequencing data. Benjamini–Hochberg correction was applied to correct the *P*‐values for multiple testing. DE genes are genes with an adjusted *P*‐value < 0.05 and a log_2_ratio > 1 or < −1.

## Author contributions

**Junhua Xie:** Conceptualization; data curation; formal analysis; investigation; visualization; methodology; writing – original draft. **Arnout Bruggeman:** Conceptualization; investigation; methodology; writing – review and editing. **Clint De Nolf:** Software; formal analysis; methodology; writing – review and editing. **Charysse Vandendriessche:** Investigation. **Griet Van Imschoot:** Investigation. **Elien Van Wonterghem:** Investigation; project administration. **Lars Vereecke:** Resources. **Roosmarijn E Vandenbroucke:** Conceptualization; resources; data curation; supervision; funding acquisition; methodology; project administration; writing – review and editing.

## Disclosure and competing interests statement

The authors declare that they have no conflict of interest.

## Supporting information



Appendix S1

Expanded View Figures PDF

Dataset EV1

Dataset EV2

Dataset EV3

Dataset EV4

Dataset EV5

Source Data for Expanded View and Appendix

PDF+

Source Data for Figure 2

Source Data for Figure 3

Source Data for Figure 4

Source Data for Figure 5

Source Data for Figure 6

Source Data for Figure 7

## Data Availability

The datasets used in this study are available in the following databases: RNA‐Seq data have been deposited in the Gene Expression Omnibus (GEO) with accession number GSE228655 (https://www.ncbi.nlm.nih.gov/geo/query/acc.cgi?acc=GSE228655); immunohistochemistry data have been deposited in the BioImage Archive (BIA) with accession number S‐BIAD609 (https://www.ebi.ac.uk/biostudies/bioimages/studies/S‐BIAD609). Quantitative data that support the findings in this study are available within the paper and in the source data.
