# Supplementary material for: Gut microbiota regulates blood‐cerebrospinal fluid barrier function and Aβ pathology
Source: EMBO J. 2023 Jul 10;42(17):e111515. doi: 10.15252/embj.2022111515 (PMC10476279; doi:10.15252/embj.2022111515)
Supplement: Supplementary file 1 — Appendix S1 [file EMBJ-42-e111515-s015.pdf]

Appendix

Table of content	
**Content**	**Page**
Appendix Method	
MTT assay	2
TUNEL staining	2
Appendix Figure	
Appendix Figure S1	3
Appendix Figure S2	4
Appendix Figure S3	6
Appendix Figure S4	7
Appendix Figure S5	8
Appendix Figure S6	9
Appendix Figure S7	10
Appendix Figure S8	11
Appendix Figure S9	12
Appendix Figure S10	13
Appendix Figure S11	14
Appendix Figure S12	15
Appendix Figure S13	16
Appendix Figure S14	17
Appendix Table	
Appendix Table S1	18

Appendix Methods

MTT assay

The untreated and LPS treated primary CP epithelial cells were replaced with 100 µl fresh medium and incubated for 4 h with 10 µl of the 12 mM MTT. Added 100 µl of the SDS-HCl solution (1 g SDS in 10 ml of 0.01 M HCl) to each well and mixed thoroughly using the pipette and incubated the microplate at 37°C for 4 h in a humidified chamber. Mixed each sample again using a pipette and read absorbance at 570 nm (iMark Microplate Absorbance Reader, Bio-Rad). Results are presented as percentage of the control values.

TUNEL staining

TUNEL staining was conducted using a commercial kit (In Situ Cell Death Detection Kit; Roche), following the manufacturer's instructions. In brief, primary choroid plexus epithelial (CPE) cells were cultured in 8 well-chamber (ibidi) and post-fixed with 2%PFA for 15 min. Subsequently, the CPE cells were incubated with the reaction mixture containing terminal deoxynucleotidyl transferase (TdT) and fluorescein-conjugated deoxyuridine triphosphate (dUTP) for 1 h at 37 °C. After washing with PBS, the sections were blocked and permeabilized in GIM at RT for 1h.

A

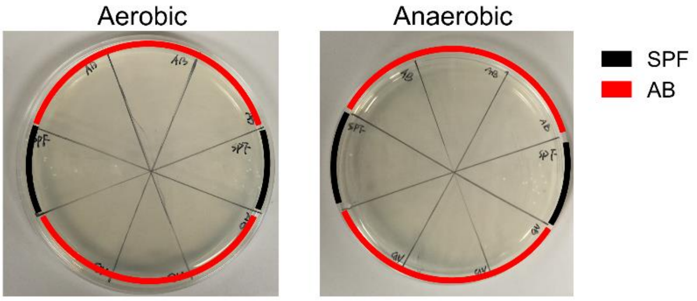

B

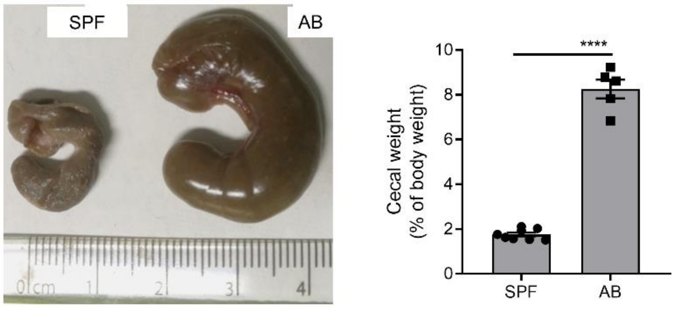

Appendix Figure S1. Validation of antibiotics treatment.

A Bacterial growth of faeces from SPF and AB mice in BHI plate for 24 h under aerobic (left) and anaerobic (right) conditions.

B Representative pictures of caeca from SPF and AB mice (left) and relative cecal weight (to body weight) of SPF and AB mice (right) (n=5-8).

Data information: Bars represent mean ± SEM. Statistics were performed with unpaired t test, ****p < 0.00001. AB, antibiotics-treated; BHI, brain heart infusion; SPF, specific pathogen-free

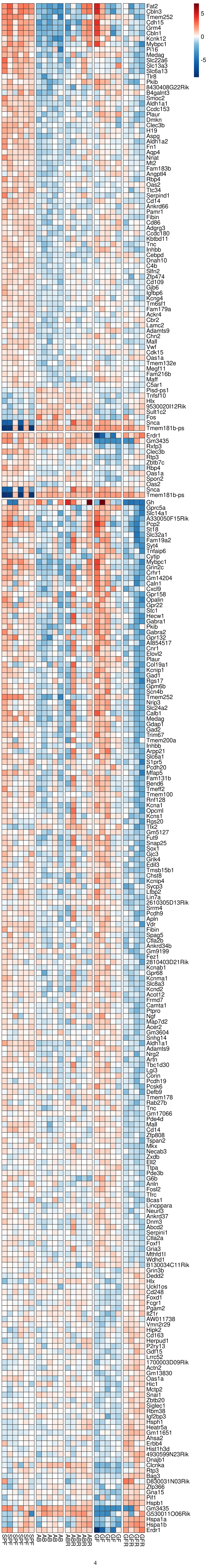
5
0
-5
Fat2
Cbln3
Tmem252
Cdh15
Grm4
Cbln1
Kcnk12
Mybpc1
Pi16
Medag
Slc22a6
Slc13a3
Slc6a13
Tlr8
Pkib
8430408G22Rik
B4galnt3
Smoc2
Aldh1a1
Ccdc153
Plaur
Dmkn
Clec3b
H19
Aspg
Aldh1a2
Fn1
Aqp4
Nnat
Mt2
Fam183b
Angptl4
Rbp4
Oas2
Ttc34
Serpind1
Cd14
Ankrd66
Pamr1
Fibin
Cd86
Adgrg3
Ccdc180
Kbtbd11
Tnc
Inhbb
Cebpd
Dnah10
C4b
Slfn2
Zfp474
Cd109
Gjb6
Igfbp6
Kcng4
Tm6sf1
Fam179a
Ackr4
Cbr2
Lamc2
Adamts9
Chn2
Mall
Vwf
Cdk15
Oas1a
Tmem132e
Megf11
Fam216b
Maff
C5ar1
Pisd-ps1
Tnfsf10
Hlx
9530020I12Rik
Sult1c2
Fos
Snca
Tmem181b-ps
Erdr1
Gm3435
Rxfp3
Clec3b
Rtp3
Zbtb7c
Rbp4
Oas1a
Spon2
Oas2
Snca
Tmem181b-ps
Gh
Gprc5a
Slc14a1
A330050F15Rik
Pcp2
St18
Slc32a1
Fam19a2
Syt4
Tnfaip6
Cytip
Mybpc1
Grin2c
Crhr1
Gm14204
Caln1
Cxcl9
Gpr158
Opalin
Gpr22
Stc1
Hecw1
Gabra1
Pkib
Gabra2
Gpr132
AI854517
Cnr1
Elovl2
Plaur
Col19a1
Kcnip1
Gad1
Rgs17
Gpm6b
Scn4b
Tmem252
Nrip3
Slc24a2
Calb1
Medag
Gdap1
Gad2
Trim67
Tmem200a
Inhbb
Arpp21
Slc6a1
S1pr5
Pcdh20
Mfap5
Fam131b
Bend6
Tmeff2
Tmem100
Rnf128
Kcna1
Opcml
Kcns1
Rgs20
Tlx2
Gm5127
Fut9
Snap25
Sox1
Gjc3
Grik4
Edil3
Tmsb15b1
Chst8
Kcnip4
Sycp3
Ltbp2
Lin7a
2610305D13Rik
Srrm4
Pcdh9
Apln
Vdr
Fibin
Spag5
Ctla2b
Ankrd34b
Gm9199
Fez1
2810403D21Rik
Kcnab1
Gpr68
Kcnma1
Slc8a3
Kcnd2
Acot12
Frmd7
Camta1
Ptpro
Ngf
Map7d2
Acer2
Gm3604
Snhg14
Aldh1a1
Adamts9
Nrg2
Artn
Tbc1d30
Lgi3
Corin
Pcdh19
Pcsk6
Defb9
Tmem178
Rab27b
Tnc
Gm17066
Pde4d
Mall
Cd14
Zfp808
Tspan2
Mkx
Necab3
Zxdb
Ell2
Ttpa
Pde3b
G6b
Anln
Fosl2
Tfrc
Bcas1
Lincppara
Neurl3
Ankrd37
Dnm3
Abcd2
Serpini1
Ctla2a
Foxf1
Gria3
Mthfd1l
Wdhd1
B130034C11Rik
Grin3b
Dedd2
Hlx
Uckl1os
Cd248
Foxd1
Fcgr1
Pgam2
Il21r
AW011738
Vmn2r29
Hipk2
Cd163
Herpud1
P2ry13
Gdf15
Lrrc52
1700003D09Rik
Actn2
Gm13830
Oas1a
Hic1
Mctp2
Snai1
Zbtb20
Siglec1
Rbm38
Igf2bp3
Hsph1
Heatr5a
Gm11651
Ahsa2
Erbb4
Hist1h3d
4930599N23Rik
Dnajb1
Clcnka
Rtp3
Bag3
D830031N03Rik
Zfp366
Gna15
Pif1
Hspb1
Gm3435
G530011O06Rik
Hspa1a
Hspa1b
Erdr1
SPF
SPF
SPF
SPF
SPF
SPF
AB
AB
AB
AB
AB
ABR
ABR
ABR
ABR
ABR
GF
GF
GF
GF
GF
GFR
GFR
GFR
GFR

Appendix Figure S2. Overview of Differential Expression Analysis (DEA) results of relevant comparisons between the conditions

Heatmap of all the DE genes in choroid plexus across 4 comparisons: antibiotics-treated (AB) *vs* specific pathogen free (SPF) mice (79), recolonized AB (ABR) *vs* AB mice (0), Germ-free (GF) *vs* SPF mice (12), recolonized GF (GFR) *vs* GF mice (191). There are 282 rows in the heatmap representing 259 unique genes. The comparisons are separated from each other with an empty row. Within each comparison the genes are ordered in descending order according to logFC. The color scale of the heatmap represents the scaled log2 normalized gene expression.

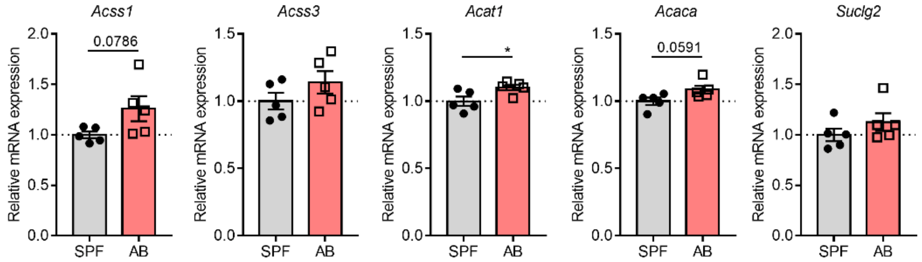

Appendix Figure S3. qPCR analysis for genes linked to the KEGG pathway mmu00640 (Propanoate metabolism) on choroid plexus of SPF and AB mice.

Data information: Bars represent mean ± SEM (n=4-5). Statistics were performed with two-tailed Student's t-test, **p* < 0.05. AB, antibiotics-treated; SPF, specific pathogen-free.

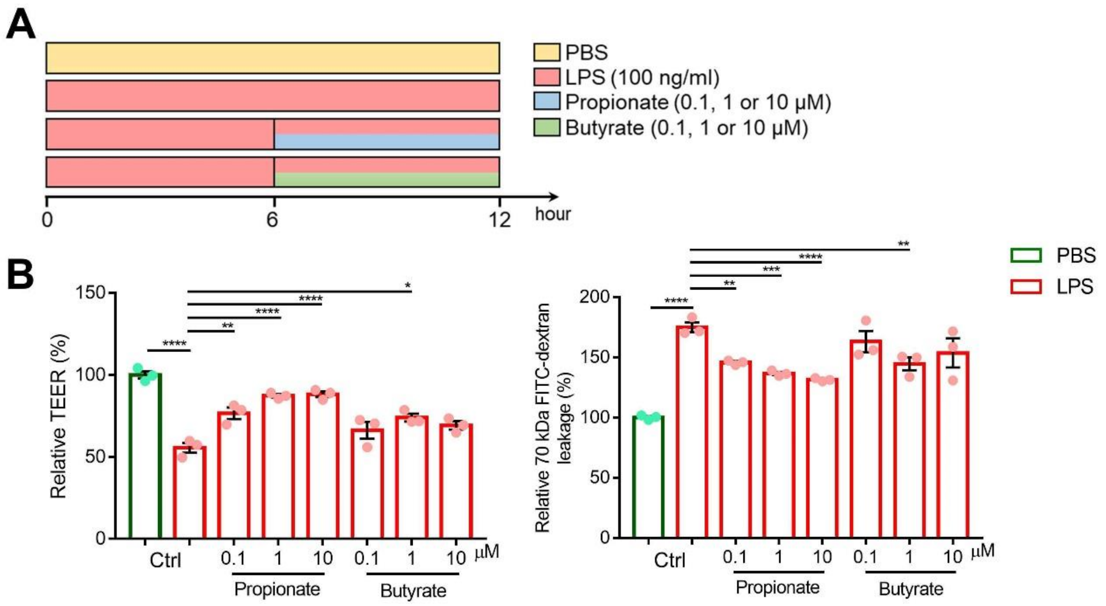

Appendix Figure S4. Restoring effects of SCFAs against LPS-induced blood-CSF barrier disruption in primary CPE cells.

A Schematic representation of the experimental conditions.

B TEER and assessment of the 70 kDa FITC-dextran paracellular permeability of primary CPE cells treated as showed in (**A**) (n=3 duplicates).

Data information: Bars represent mean ± SEM. Statistics were performed with one-way ANOVA Bonferroni's post hoc test for multiple comparisons. **p* < 0.05, ***p* < 0.01, ****p* < 0.001, *****p* < 0.0001. CPE, choroid plexus epithelial; CSF, cerebrospinal fluid; LPS, lipopolysaccharides; SCFAs, short-chain fatty acids; TEER, trans-epithelial electrical resistance.

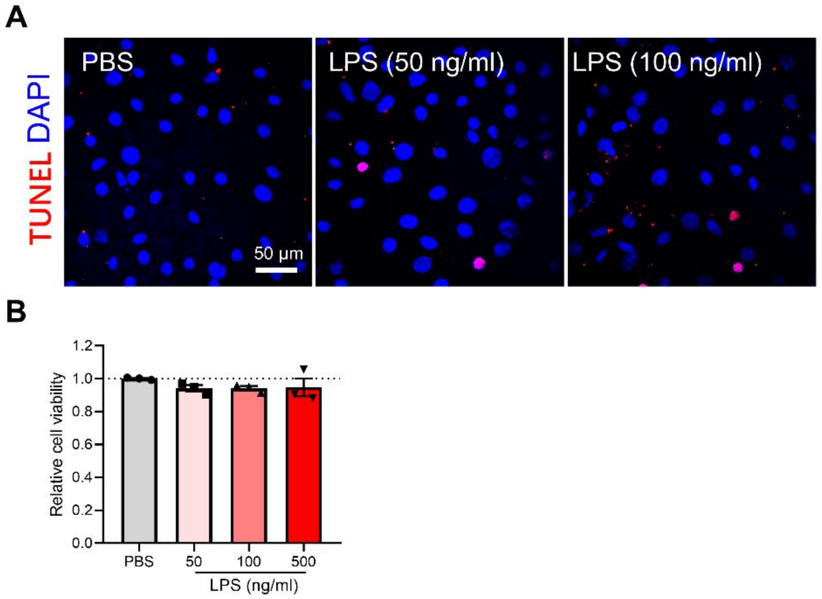

Appendix Figure S5. Effects of LPS stimulation on cell viability.

A Representative images of TUNEL staining of LPS-treated primary choroid plexus epithelial cells. Scale bar: 20 μm.

B MTT-based cell viability assay (n=3 duplicates).

Data information: Bars represent mean ± SEM. Statistics were performed with one-way ANOVA Bonferroni's post hoc test for multiple comparisons. LPS, lipopolysaccharides.

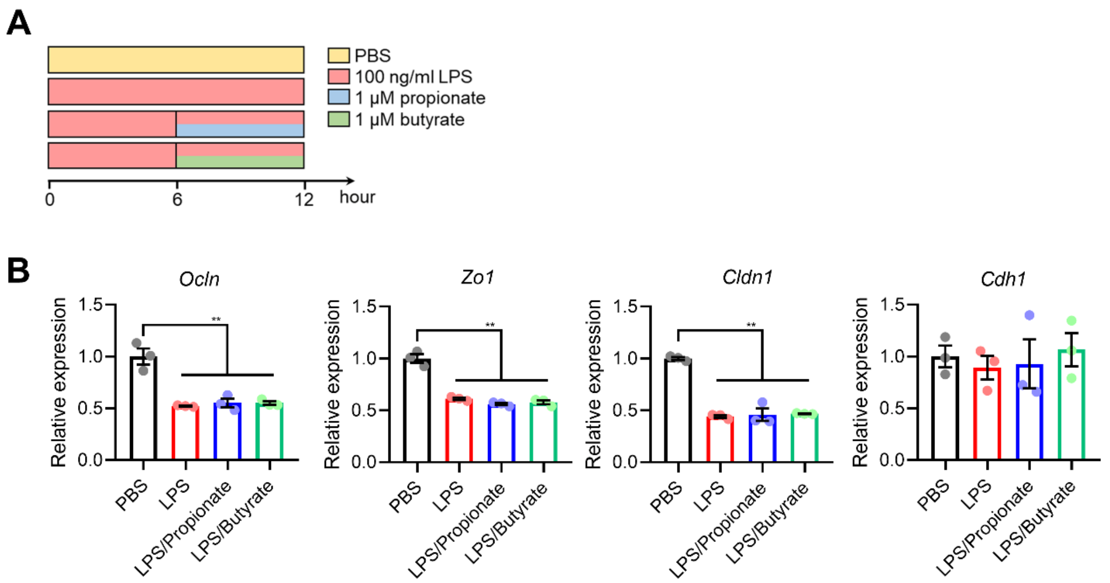

Appendix Figure S6. Effects of SCFAs on the gene expression of TJs in the choroid plexus.

A Schematic representation of the experimental conditions.

B Gene expression of tight junction in primary choroid plexus epithelial cells following treatment for 6 h with 1 μM sodium propionate or 1 μM sodium butyrate, with 100 ng/ml LPS stimulation (n=3 duplicates).

Data information: Bars represent mean ± SEM. Statistics were performed with one-way ANOVA Bonferroni's post hoc test for multiple comparisons. $^{**}p < 0.01$. LPS, lipopolysaccharides; SCFAs, short-chain fatty acids; TJs, tight junctions.

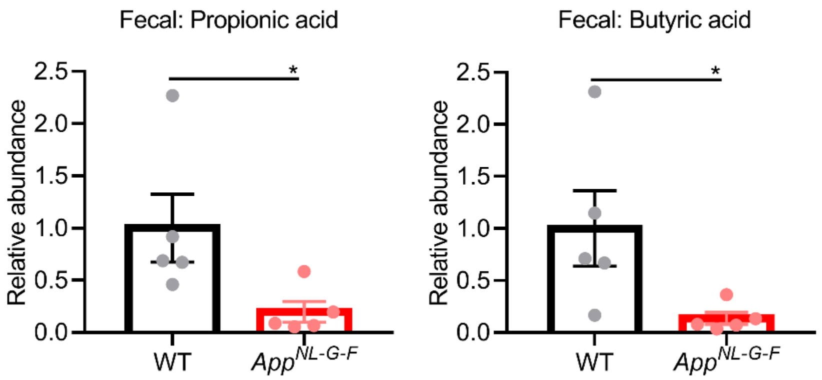

Appendix Figure S7. The SCFAs levels in fecal pellets of *App*$^{NL\text{-}G\text{-}F}$ mice.

Relative abundance of propionic acid (left) and butyric acid (right) in mice fecal samples (n=5).

Data information: Bars represent mean ± SEM. Statistics were performed with unpaired t test. *$p < 0.05$. SCFAs, short-chain fatty acids; WT, wild type.

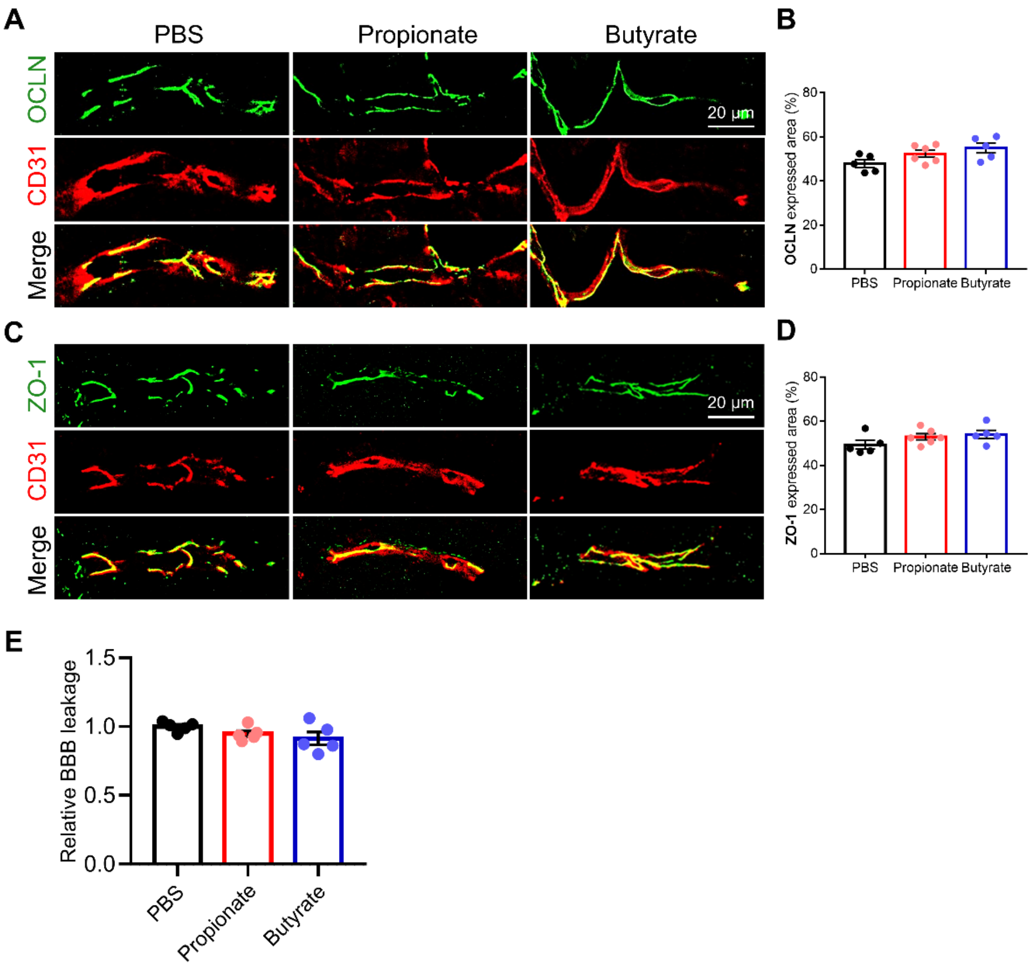

Appendix Figure S8. Effects of SCFAs on BBB integrity in *App*$^{NL\text{-}G\text{-}F}$ mice.

A Representative images of immunostaining for OCLN and CD31 in cortex. Scale bar: 20 μm.

B The percentage of expressed area of OCLN displayed in (**A**) (n=5).

C Representative images of immunostaining for ZO-1 and CD31 in cortex. Scale bar: 20 μm.

D The percentage of ZO-1 expressed area (n=5).

E Assessment of the BBB permeability to 4 kDa FITC-dextran (5-10).

Data information: Bars represent mean ± SEM. Statistics were performed with one-way ANOVA Bonferroni's post hoc test for multiple comparisons. BBB, blood-brain barrier; SCFAs, short-chain fatty acids.

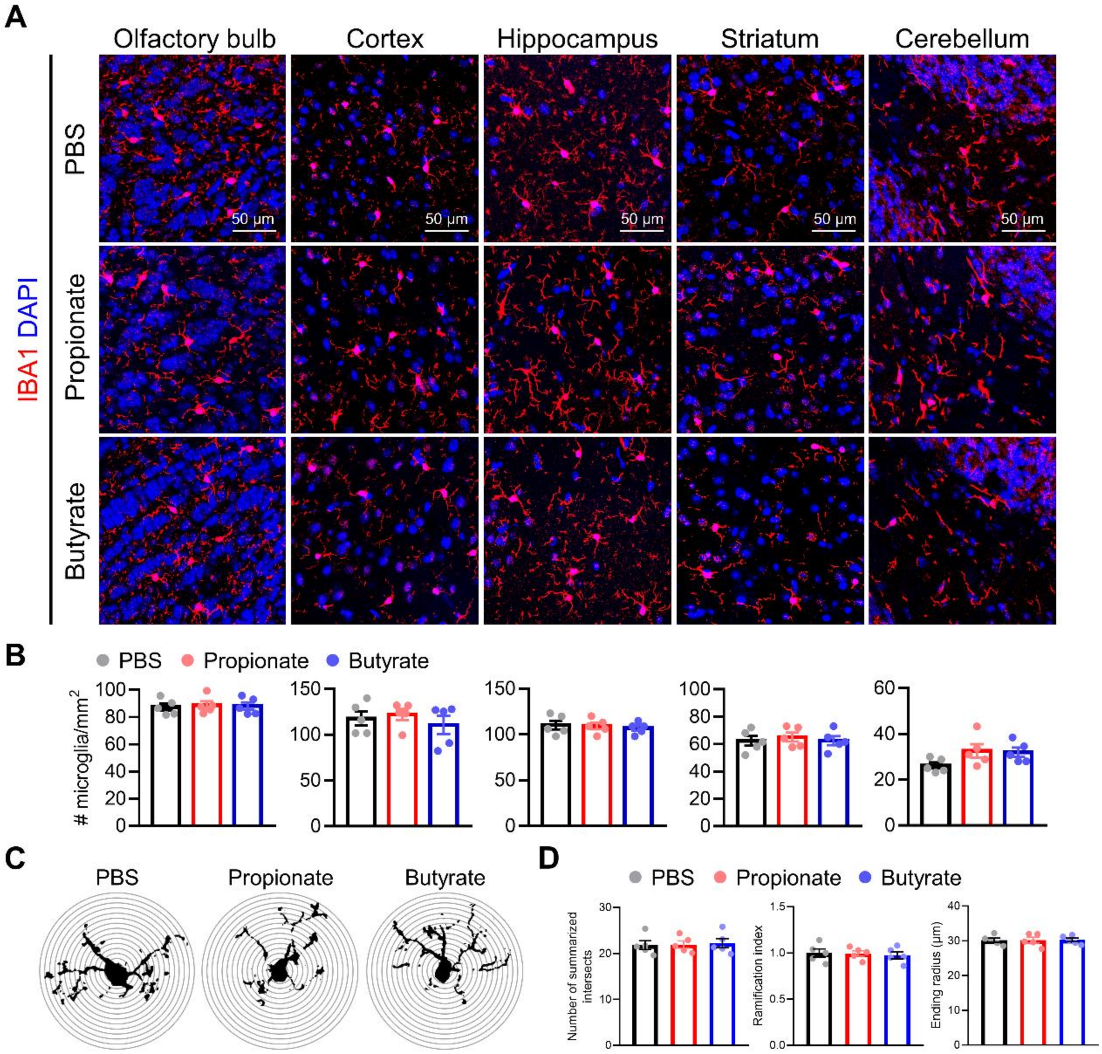

Appendix Figure S9. The effects of SCFAs on microglial proliferation and activation in SPF mice.

A Representative images of immunostainings for IBA1 in different regions of brain of sodium propionate or sodium butyrate-treated WT mice. Scale bar: 50 μm.

B The number of IBA1$^+$ astrocytes in different regions (n=5).

C Representative image for Sholl analysis of microglia in the hippocampus from the IBA1-stained image in **A**. The interval of the concentric circles is 2 μm.

D Quantification of summarized intersects, ramification index and ending radius by Sholl analysis from immunostained IBA1 signals (n=5).

Data information: Bars represent mean ± SEM. Statistics were performed with one-way ANOVA Bonferroni's post hoc test for multiple comparisons. SCFAs, short-chain fatty acids; SPF, specific-pathogen-free; WT, wild type.

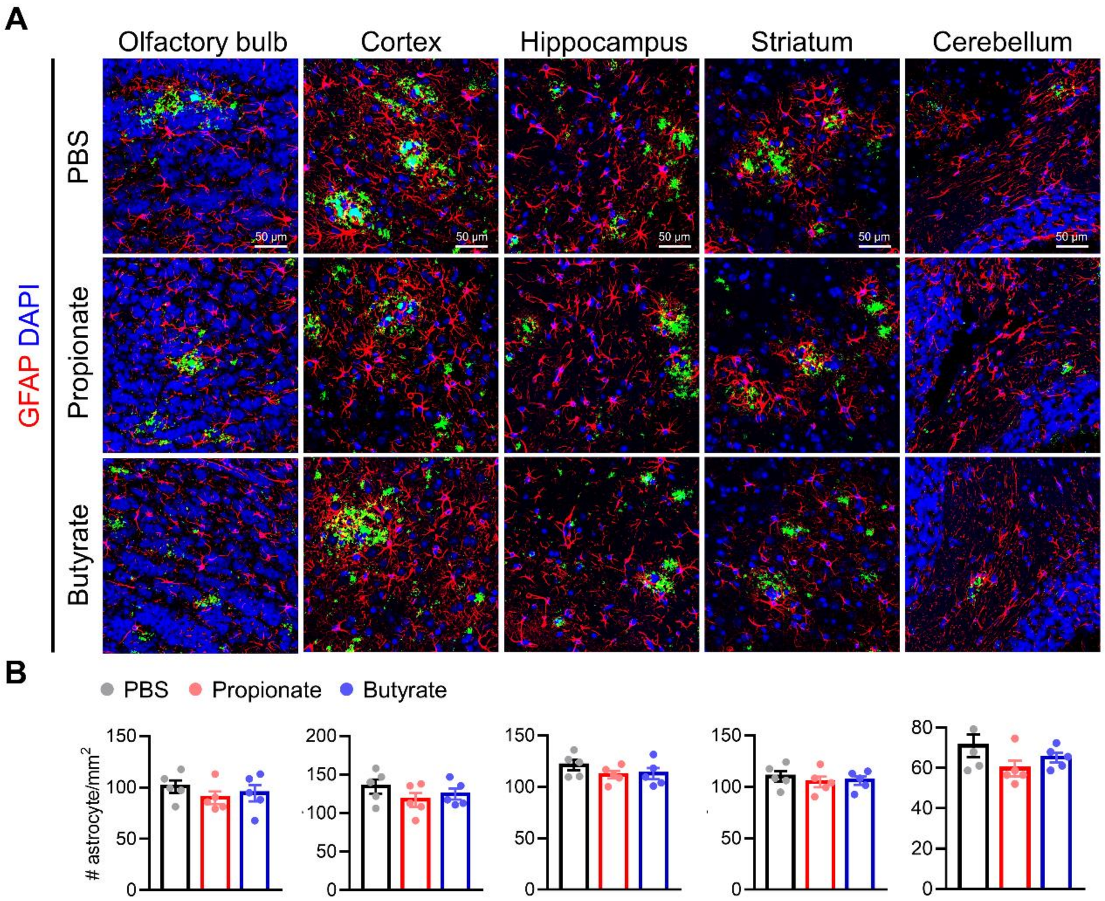

Appendix Figure S10. The effects of SCFAs on astrocyte proliferation and activation in *App*$^{NL\text{-}G\text{-}F}$ mice.

A Representative images of immunostainings for GFAP and 6E10 in hippocampus of sodium propionate or sodium butyrate-treated *App*$^{NL\text{-}G\text{-}F}$ mice. Scale bars, 5 μm.

B The number of GFAP^{+} astrocytes in different regions (n=5).

Data information: Bars represent mean ± SEM. Statistics were performed with one-way ANOVA Bonferroni's post hoc test for multiple comparisons. SCFAs, short-chain fatty acids.

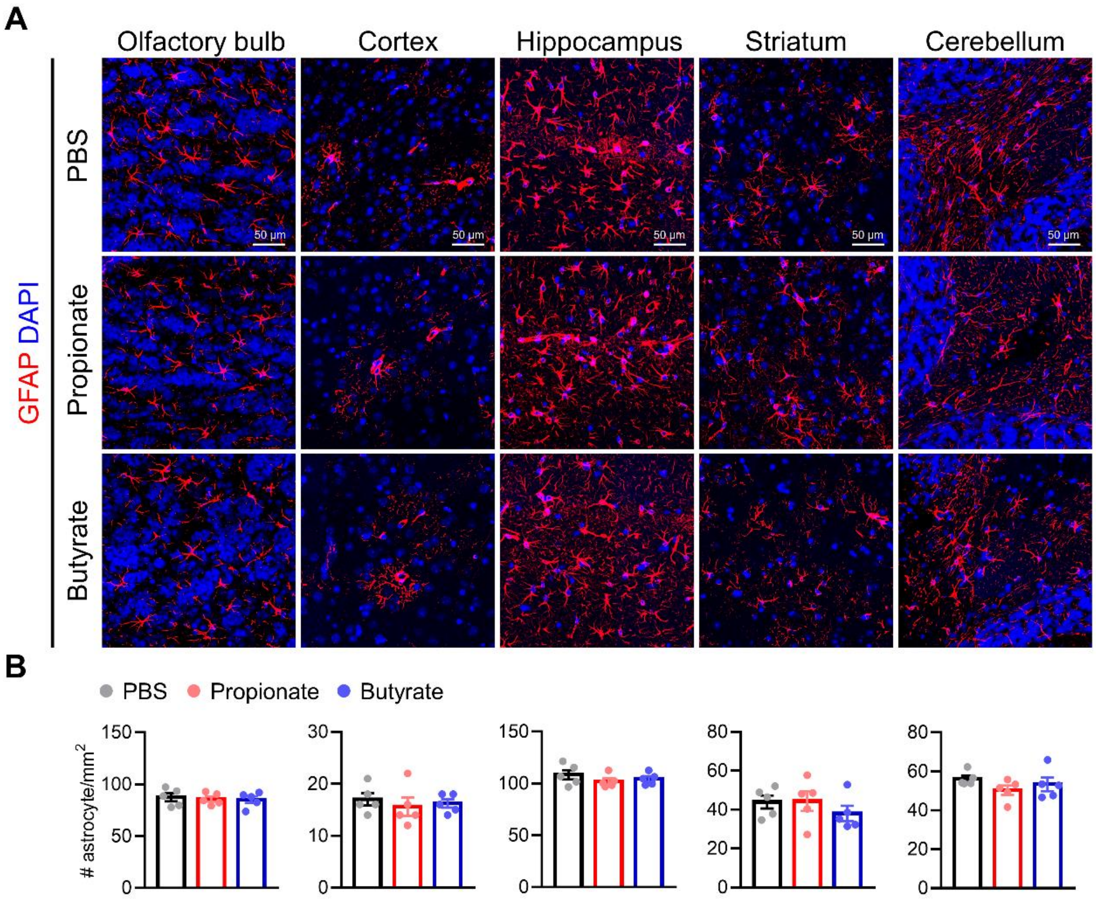

Appendix Figure S11. The effects of SCFAs on astrocyte proliferation and activation in SPF mice.

A Representative images of immunostainings for GFAP in hippocampus of sodium propionate or sodium butyrate-treated WT mice. Scale bars, 5 μm.

B The number of GFAP⁺ astrocytes in different regions (n=5).

Data information: Bars represent mean ± SEM. Statistics were performed with one-way ANOVA Bonferroni's post hoc test for multiple comparisons. SCFAs, short-chain fatty acids; SPF, specific-pathogen-free.

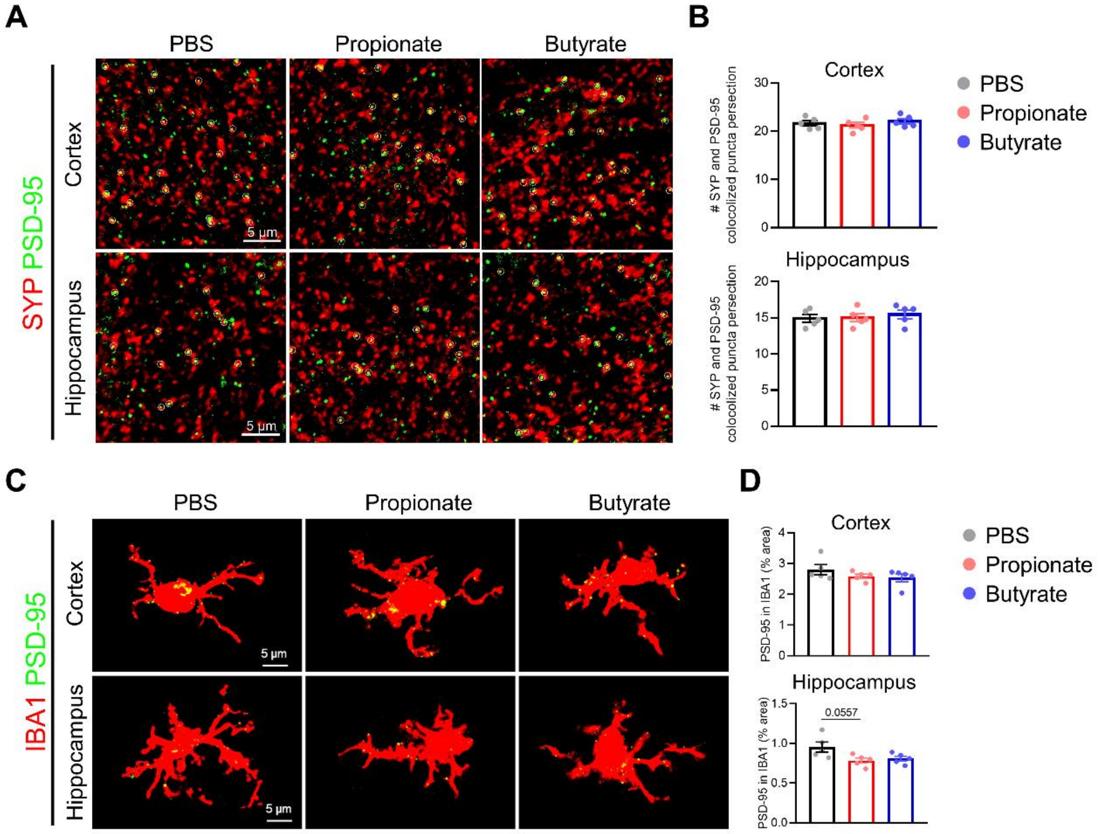

Appendix Figure S12. The effects of SCFAs on synapse elimination by microglia in *App*$^{NL\text{-}G\text{-}F}$ mice.

A Representative high magnification confocal images of SYP and PSD-95 coimmunostaining in cortex and hippocampus of *App*$^{NL\text{-}G\text{-}F}$ mice. Scale bars, 5 µm.

B Quantification of the number of colocalized puncta of SYP and PSD-95 (n=5).

C Representative images of IBA1 and PSD-95 coimmunostaining in cortex and hippocampus of *App*$^{NL\text{-}G\text{-}F}$ mice.

D Quantification of the internalized PSD-95 in microglia (n=5).

Data information: Bars represent mean ± SEM. Statistics were performed with one-way ANOVA Bonferroni's post hoc test for multiple comparisons. PSD-95, postsynaptic density protein 95; SCFAs, short-chain fatty acids; SPF, specific-pathogen-free; SYP, synaptophysin..

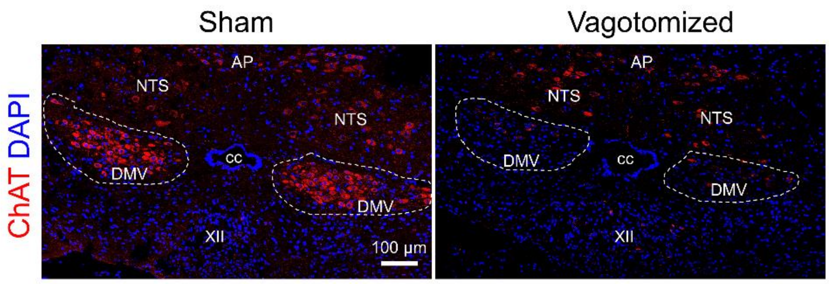

Appendix Figure S13. Validation of vagotomy by ChAT staining on DMV. Representative images of ChAT immunostaining on DMV of sham and vagotomized mice. Scale bar: 100 μm. AP, area postrema; CC, central canal; DMV, dorsal motor nucleus of the vagus; NTS, nucleus of the solitary tract XII, Hypoglossal nucleus.

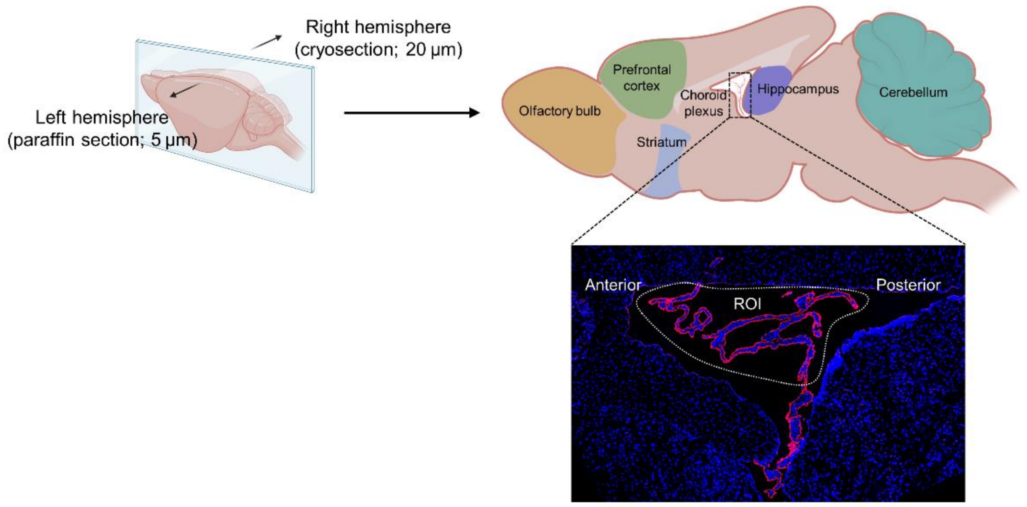

Appendix Figure S14. Sagittal section of adult mice brain. ROI: region of interest.

Appendix Table S1. List of primer sequences used for RT-qPCR analysis.

Gene	Forward primer sequence (5'-3')	Reverse primer sequence (5'-3')
Hprt	AGTGTTGGATACAGGCCAGAC	CGTGATTCAAATCCCTGAAGT
Rpl	CCTGCTGCTCTCAAGGTT	TGGTTGTCACTGCCTGGTACTT
Ubc	AGGTCAAACAGGAAGACAGACGTA	TCACACCCAAGAACAAGCACA
Ocln	CCAGGCAGCGTGTTCCT	TTCTAAATAACAGTCACCTGAGGGC
Cldn1	TCTACGAGGGACTGTGGATG	TCAGATTCAGC AAGGAGTCG
Cdh1	TCGGAAGACTCCCGATTCAAA	TCACACCCAAGAACAAGCACA
Zo1	AGGACACCAAAGCATGTGAG	CGGACGAGGAAACTGGTCTC
Fat2	CTTGGCGCTTCACTCACTC	GCTCTGCCAGATACATGCCC
Cbln1	GCTTTCTCTGCCATCAGGAGCA	GGCGATGAAAGTGCTGCGTTCT
Cbln3	AGTGGTGCCATCTACTTCGACC	CCTGGACAGTTTGGCGGTTGTA
Cdh15	AGGACGAGCATAGCTGAAGGAG	GTCCACTTGCAGCCAGTCTTCT
Grm4	CATCACCAAGCCTGAACGAGTG	GCGGCTGTTATCACTCAAGTCG
Kcnk12	CTGGGACTTCCCTGGAGCCTT	ACAGTCCGTAGGCGATGAGGAA
Slc22a6	GAAAGGCTGTCTGGCTTCCTCT	ATCAGTGGGCTCACTATGCTGC
Slc13a3	GGAAGGCCGATGCCTCTATG	GGAAGTTGGTGTCGAGGAAGT
Slc6a13	CAGTACACCAACCAGGGAGG	GCCAGGACAACGATGTAGTAGA
B4galnt3	GTCGCGTGGAGACGGAATG	GAGGAGCCTACATGGCTGG
Aqp4	ATGGTTCACGGGTTTGGATG	TCCAGGGTTGTAGCCAGGT
Tlr8	AAGTGCTGGACCTGAGCCACAA	CCTCTGTGAGGGTGTAAATGCC
Tmem252	GTTCAACCACGTGCTCAGACAG	GGCTCTCTTCATAAGCTGGAGG
Tmem181b-ps	TCCAGTCTCTGTTCCTGTGTGC	TATTCCCAGCGTGACAGAAGCC
Acss1	GCAGGCTATCTACTGTATGCCG	AGGACTGTGGTAGCTCCATTGC
Acss3	CAGAGTTCTCATTTACAGCCGTC	AGAGAACAGGCACGCAGTCATG
Acat1	GCAGGGAAGTTTGCCAGTGAGA	GAACACGGTCTTGAGCTTTGGC
Acaca	GTTCTGTTGGACAACGCCTTCAC	GGAGTCACAGAAGCAGCCCATT
Suclg2	AGCTCAAGGTGCCACTGGTAGT	GCTTTCTTGGCTGCATCCTCCA
