# Supplementary material for: Gut microbiota regulates blood‐cerebrospinal fluid barrier function and Aβ pathology
Source: EMBO J. 2023 Jul 10;42(17):e111515. doi: 10.15252/embj.2022111515 (PMC10476279; doi:10.15252/embj.2022111515)
Supplement: Supplementary file 2 — Expanded View Figures PDF [file EMBJ-42-e111515-s014.pdf]

Expanded View Figures

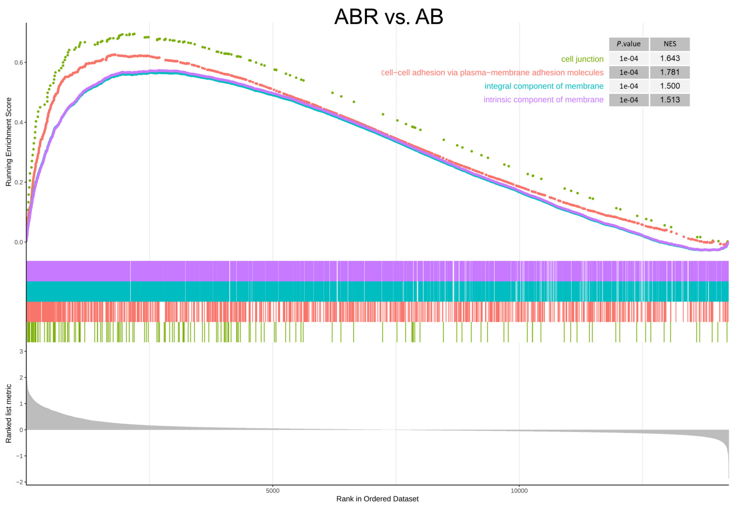

Figure EV1. GSEA of the barrier function genes in recolonized antibiotics-treated mice versus antibiotics-treated mice.

Combined GSEA plot for four GO terms performed on full gene distribution of comparison in choroid plexus between AB and ABR mice. The plot features the running enrichment scores and placement of the member genes for each respective GO term and also includes the ranked list metric plot for the full gene distribution. AB, antibiotics-treated; ABR, recolonized AB; GO, gene Ontology; GSEA, Gene Set Enrichment Analysis.

© 2023 The Authors

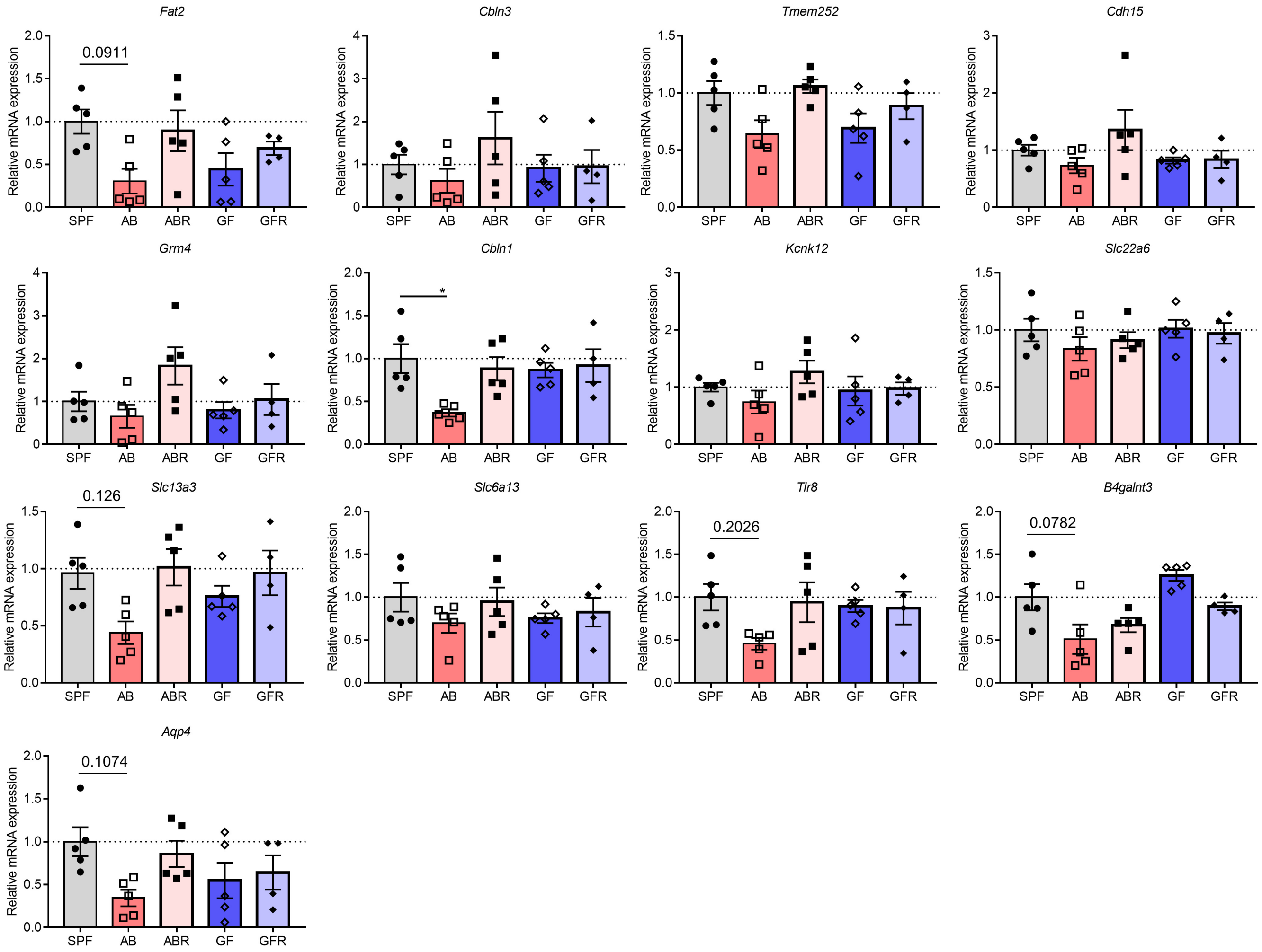

Figure EV2. qPCR analysis for barrier function-associated genes on choroid plexus of SPF, AB, ABR, GF, and GFR mice.

Data information: Bars represent mean ± SEM. n = 5, biological replicates. Statistics were performed with one-way ANOVA Bonferroni's post hoc test for multiple comparisons. *$p < 0.05$. AB, antibiotics-treated; ABR, recolonized AB; GF, germ-free; GFR, recolonized GF; SPF, specific pathogen-free.

Source data are available online for this figure.

Figure EV3. BBB integrity in mice with different gut microbiota composition.

A Representative images of immunostaining for OCLN and CD31 in cortex. Scale bar: 20 μm.

B The percentage of OCLN expressed area (n = 5, biological replicates).

C Representative images of immunostaining for ZO-1 and CD31 in cortex. Scale bar: 20 μm.

D The percentage of ZO-1 expressed area (n = 5, biological replicates).

E Assessment of the BBB permeability to 4 kDa FITC-dextran (n = 5–10, biological replicates).

Data information: Bars represent mean ± SEM. Statistics were performed with one-way ANOVA Bonferroni's *post hoc* test for multiple comparisons. *$p < 0.05$, **$p < 0.01$, ***$p < 0.001$, ****$p < 0.0001$. BBB, blood–brain barrier. AB, antibiotics-treated; ABR, recolonized AB; GF, germ-free; GFR, recolonized GF; SPF, specific pathogen-free.

Source data are available online for this figure.

© 2023 The Authors

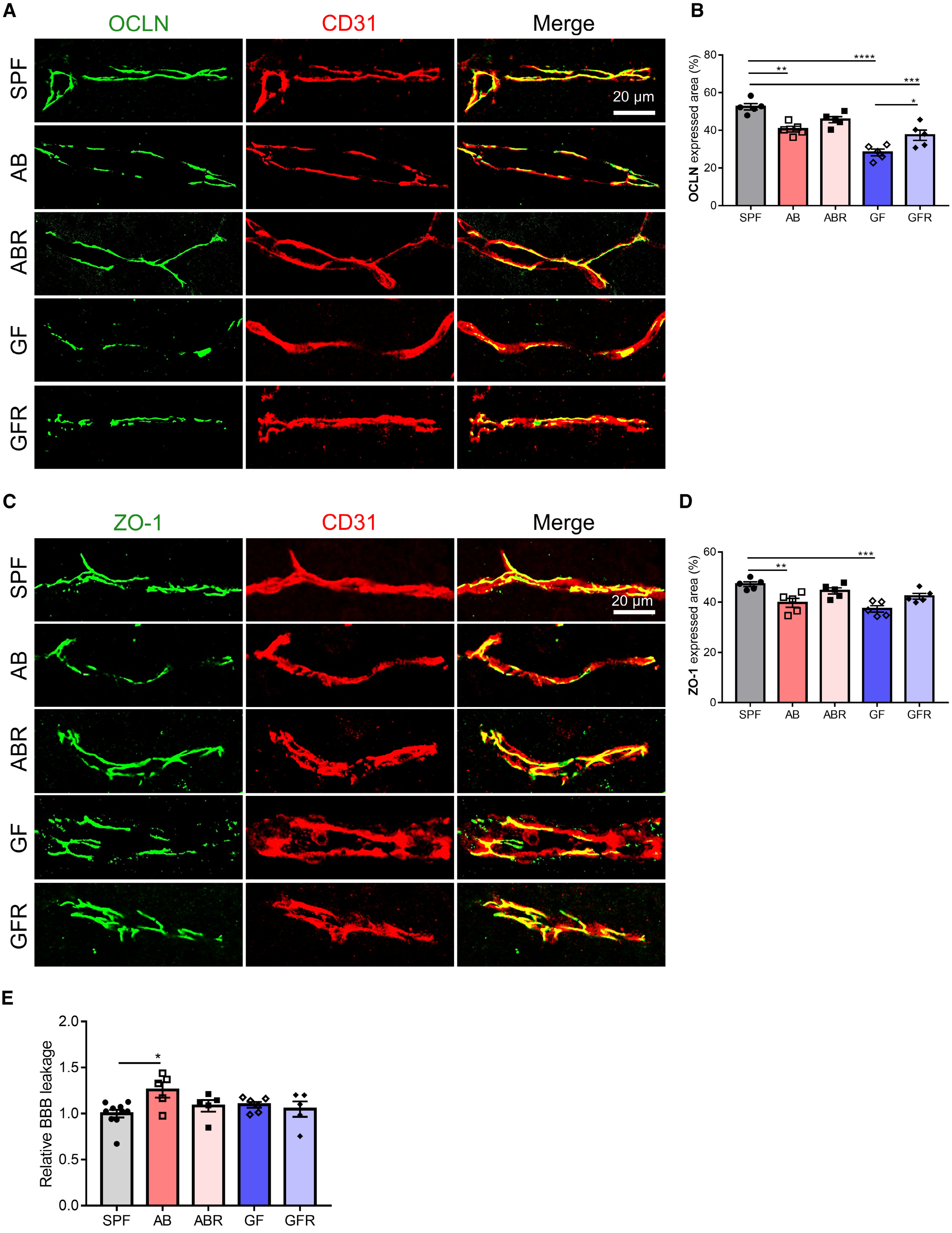

Figure EV3.

© 2023 The Authors

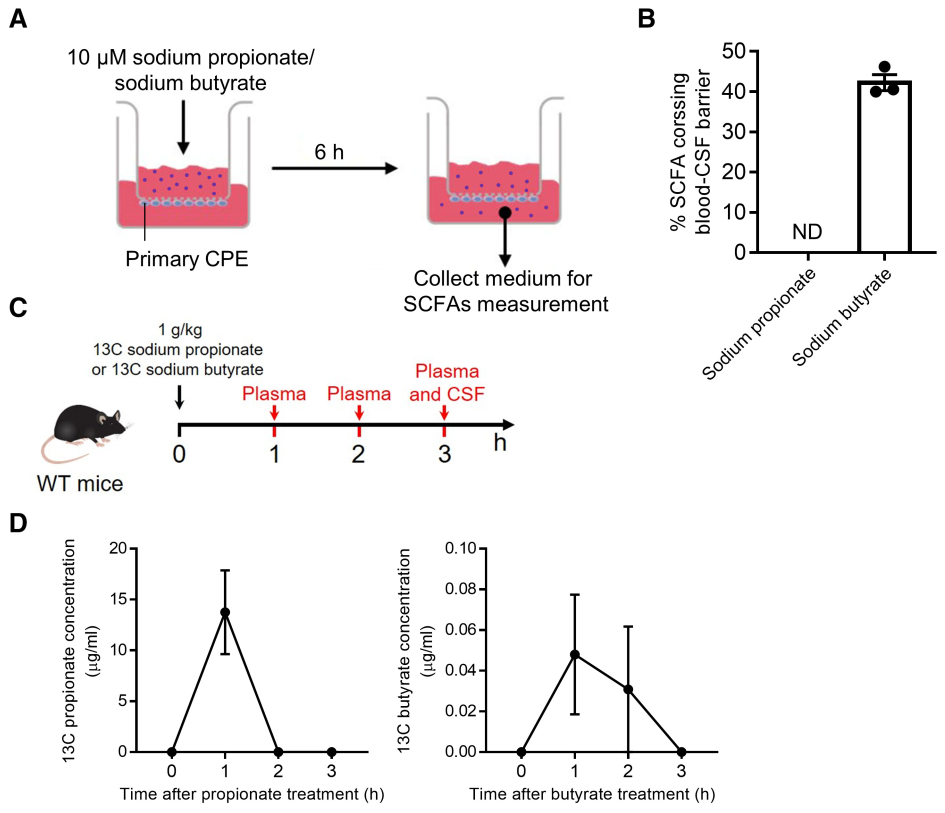

Figure EV4. Distribution of SCFAs in *in vitro* and *in vivo*.

A Schematic representation of the *in vitro* experimental conditions.
B The percentage of SCFAs crossing the blood-CSF barrier *in vitro* (n = 3, technical replicates).
C Schematic representation of the *in vivo* experimental conditions.
D The concentrations of 13C sodium propionate (left) and 13C sodium butyrate (right) in plasma (n = 5, biological replicates).

Data information: Bars represent mean ± SEM. ND, not detected; SCFAs, short-chain fatty acids.
Source data are available online for this figure.

© 2023 The Authors

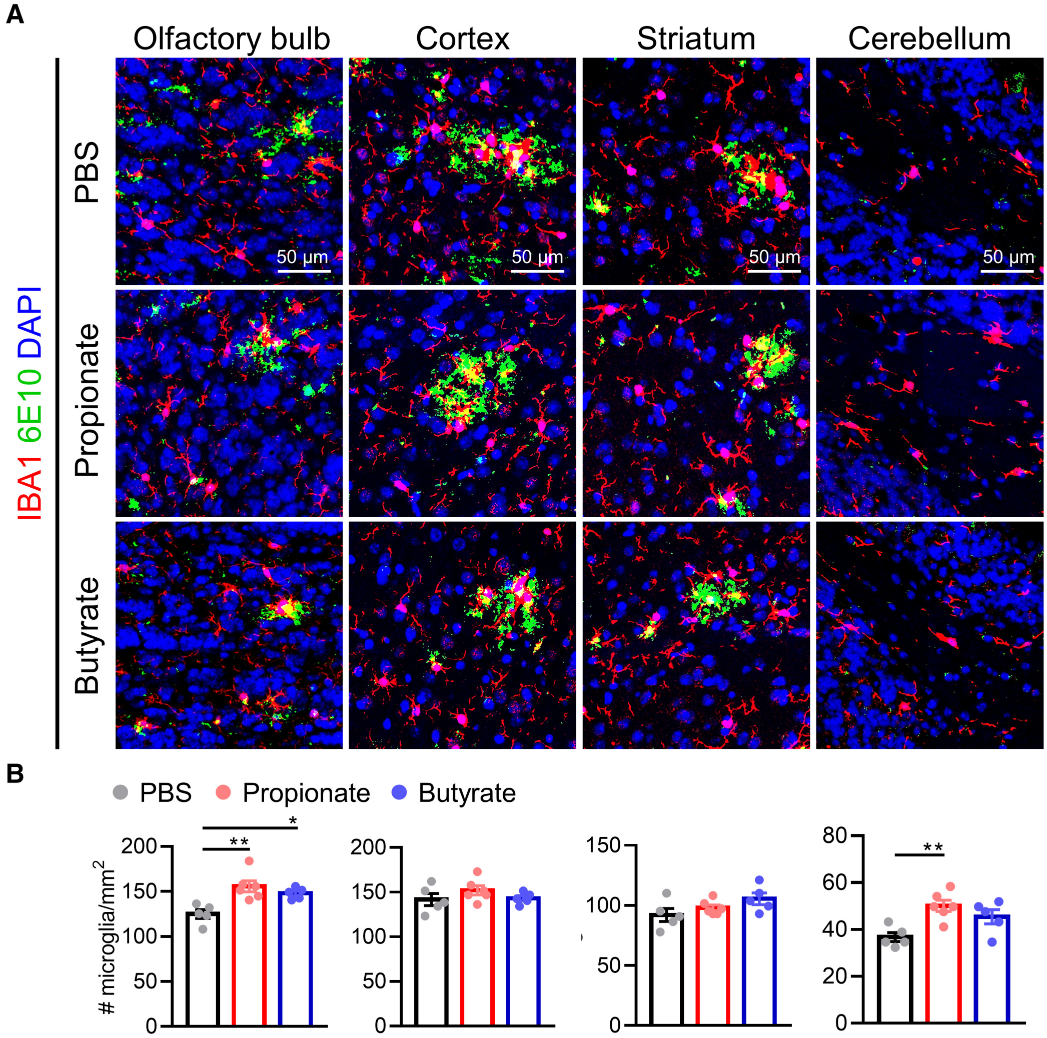

Figure EV5. Effects of SCFAs on microglial proliferation in *App$^{NL\text{-}G\text{-}F}$* mice.

A Representative images of immunostainings for IBA1 and 6E10 in olfactory bulb, cortex, striatum and cerebellum. Scale bar: 50 μm.

B The number of IBA1^{+} microglia in different regions of the brain (n = 5–6, biological replicates).

Data information: Bars represent mean ± SEM. Statistics were performed with one-way ANOVA Bonferroni's *post hoc* test for multiple comparisons. *$p < 0.05$, **$p < 0.01$. SCFAs, short-chain fatty acids.

Source data are available online for this figure.

 © 2023 The Authors
