# Supplementary figures and images for: Gut microbiota regulates blood‐cerebrospinal fluid barrier function and Aβ pathology
Source: EMBO J. 2023 Jul 10;42(17):e111515. doi: 10.15252/embj.2022111515 (PMC10476279; doi:10.15252/embj.2022111515)

**Figure 2E.** Uncropped gel

**Blot 1:** showed in Figure 2E

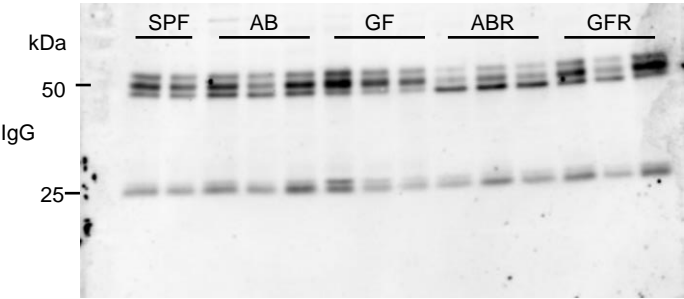

**Blot 2**

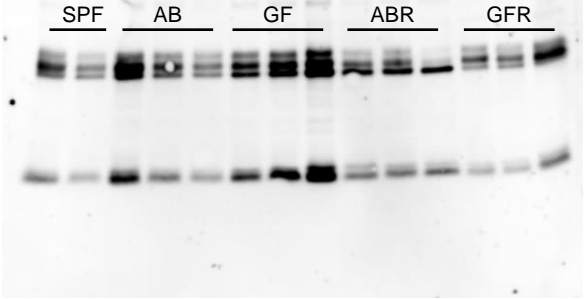

Supplement: Supplementary file 10 — Source Data for Figure 2 [file EMBJ-42-e111515-s006.zip › Source Data for Figure 2E.pdf]

**Figure 4L. Uncropped gel**

**Blot 1: showed in Figure 4L**

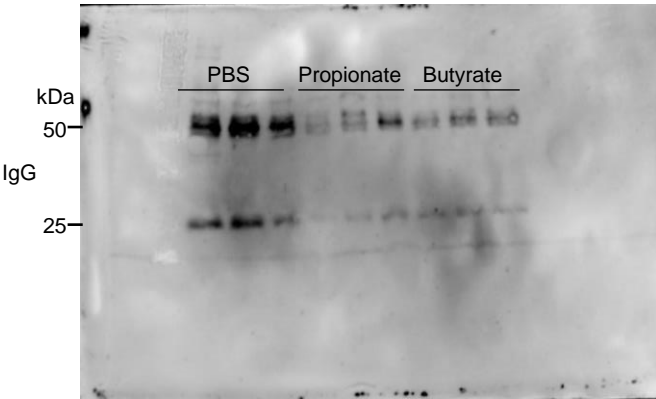

**Blot 2**

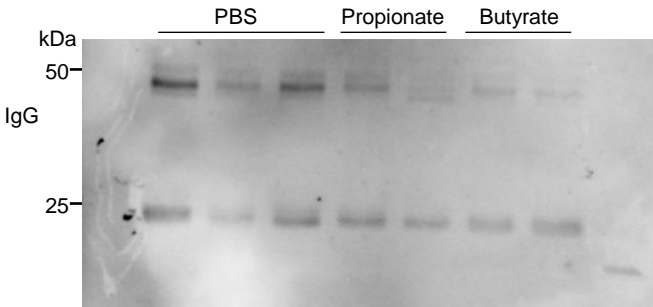

Supplement: Supplementary file 12 — Source Data for Figure 4 [file EMBJ-42-e111515-s003.zip › Source Data for Figure 4L.pdf]

**Figure 5E.** Uncropped gel

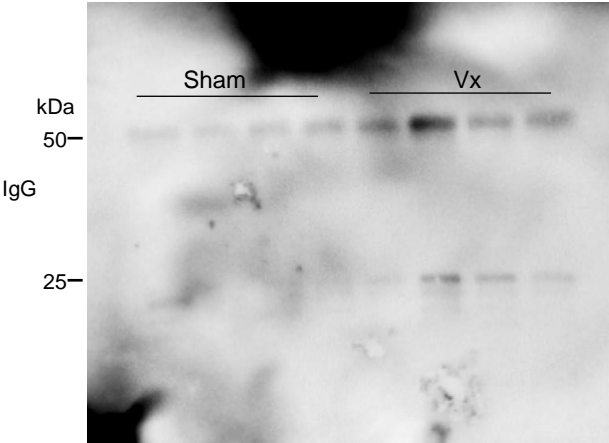

Supplement: Supplementary file 13 — Source Data for Figure 5 [file EMBJ-42-e111515-s002.zip › Source Data for Figure 5E.pdf]

**Figure 5L.** Uncropped gel

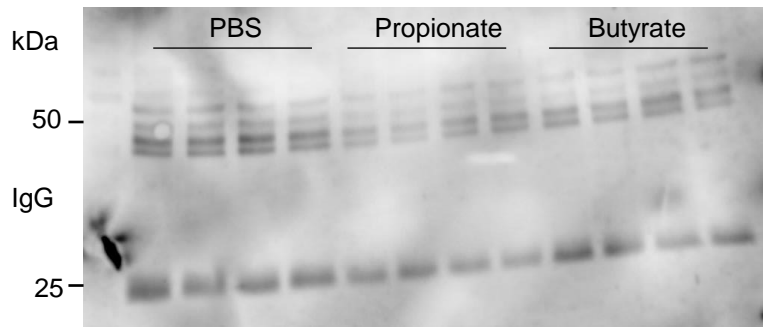

Supplement: Supplementary file 13 — Source Data for Figure 5 [file EMBJ-42-e111515-s002.zip › Source Data for Figure 5L.pdf]

**Figure 6F.** Uncropped gel

**Blot 1:** showed in Figure 6F

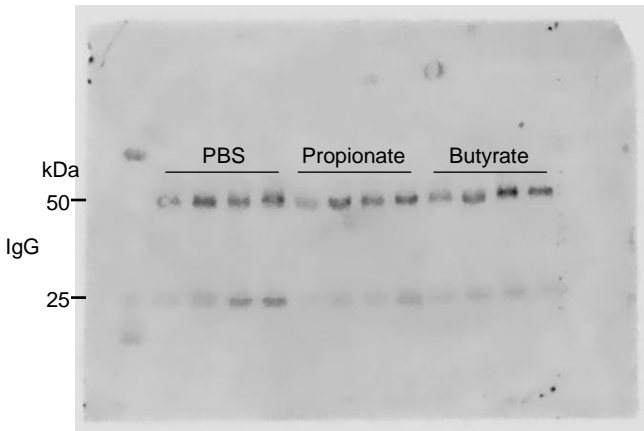

**Blot 2**

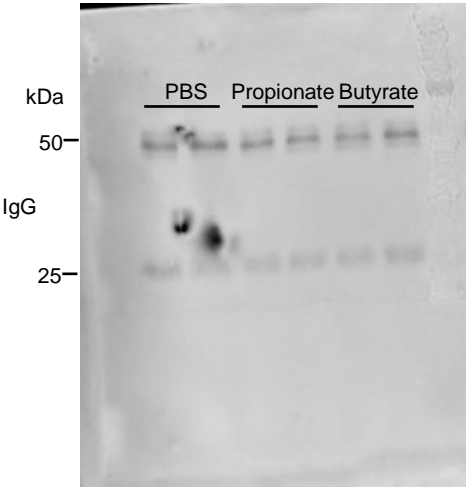

Supplement: Supplementary file 14 — Source Data for Figure 6 [file EMBJ-42-e111515-s007.zip › Source Data for Figure 6F.pdf]
