# Supplementary material for: Gut microbiota regulates blood‐cerebrospinal fluid barrier function and Aβ pathology
Source: EMBO J. 2023 Jul 10;42(17):e111515. doi: 10.15252/embj.2022111515 (PMC10476279; doi:10.15252/embj.2022111515)
Supplement: Supplementary file 16 [file EMBJ-42-e111515-s010.pdf]

THE EMBO JOURNAL

Gut microbiota regulates blood-cerebrospinal fluid barrier function and Aβ pathology

Junhua Xie, Arnout Bruggeman, Clint De Nolf, Charysse Vandendriessche, Griet Van Imschoot, Elien Van Wonterghem, Lars Vereecke, and Roosmarijn Vandenbroucke
DOI: 10.15252/embj.2022111515

Corresponding author(s): Roosmarijn Vandenbroucke (roosmarijn.vandenbroucke@irc.VIB-UGent.be)

Review Timeline:

Submission Date:	24th Apr 22
Editorial Decision:	25th May 22
Appeal Received:	7th Jun 22
Editorial Decision:	7th Jun 22
Revision Received:	16th Jan 23
Editorial Decision:	13th Mar 23
Revision Received:	20th May 23
Accepted:	2nd Jun 23

Editor: Karin Dumstrei

Transaction Report:

(Note: With the exception of the correction of typographical or spelling errors that could be a source of ambiguity, letters and reports are not edited. Depending on transfer agreements, referee reports obtained elsewhere may or may not be included in this compilation. Referee reports are anonymous unless the Referee chooses to sign their reports.)

1st Editorial Decision **25th May 2022**

Dear Roos,

Thank you for submitting your manuscript to The EMBO Journal. Your study has now been seen by three referees and I am afraid that the overall recommendation is not a positive one.

The referees appreciate the overall topic, but also raise concerns with it that I am afraid preclude publication here. They find that some of the reported data is not conclusive enough and also find that part of the analysis is missing depth and mechanistic insight.

Given this input from good experts in the field, I am afraid that I can't offer to consider publication here.

I thank you for the opportunity to consider this manuscript. I am sorry that we cannot be more positive on this occasion, but I hope nevertheless that you will find our referees' comments helpful.

with best wishes

Karin

Karin Dumstrei, PhD
Senior Editor
The EMBO Journal

****

Referee #1:

In this paper, entitled, "Gut microbiota has a major impact.....", by J., Xie., et al, the authors have studied gut microbiota/gut metabolite effects on blood-CSF barrier (BCB) permeability. They report that mice lacking gut microbiota, display increased BCB associated with changes in the organization of tight junctions (TJs). The authors also report that gut microbiota or supplementation with short-chain fatty acids (SCFAs) restore the organization of TJs. These effects are still observed in "vagotomized" mice suggesting an n.vagus independent mechanism. Finally, the authors report that SCFA administration to AppNL-G-F AD transgenic mice improves the BCB barrier as monitored by a reduction in Aβ burden and changes in microglial phenotype.
General comments.
The topic is clearly of interest and highly relevant to a broader scientific community. However, while positive about the topic and the well-written manuscript, the lack of controls reduces my enthusiasm somewhat
More specifically
1. In figure 1 they display the gene expression profile of SPF mice and AB treated mice but not GF mice? Why? There should be consistency across the figures with all groups presented in the same way across the figures. Please include the expression data from GF and align accordingly.
2. The lack of quantification of TJs WB blot data is surprising. The authors must include WB data in all the data sets monitoring TJs is displayed.
3. The use of IgG quantification provides information about the degree of leakiness. Please increase the n numbers in Fig 4 and Fig 6d to n=5. N=3 is not sufficient
4. It would also be advisable if the authors could consider including another tracer to further document changes in BCB without SCFA and SPF vs GF and AB treated mice
5. The microscopic pictures in Figure 7 are overall of less good quality. Please provide higher resolution photos

Referee #2:

In this study, Xie et al. compared mice with vs. without gut microbiota to demonstrate that removal of gut microbiota negatively impacts the choroid plexus epithelial (CPE) tight junction distribution and barrier functions. They further showed that SCFA produced by gut microbes, specifically propionate and butyrate, can rescue tight junction damage in an in vitro LPS model. This effect appears to be independent of vagus nerve. Finally, they studied a genetic model of Alzheimer's disease (APPNL-F) and where they suggest that propionate and butyrate treatment alleviated Ab pathology and altered microglia phenotypes.

The very interesting and strongest part of this study is presented in the first half of the paper - demonstration that the blood-CSF barrier protein expression and localization depends on the gut microbiome is striking. However, overall, the study jumps around

from topic to topic, without delving sufficiently deep into any single subject / mechanisms involved, and draws strong conclusions based on a limited number of experiments and small sample sizes. The vagus nerve is an appealing topic, but it doesn't add very much to the present study since it is unlikely to be involved. The last figure with the AD data seems preliminary and could be taken out and developed into a separate study. Below are more details about concerns regarding experimental design and data interpretation:

1. The study started with a bulk RNAseq analysis of the choroid plexus from mice with healthy microbiota vs. mice treated with antibiotics. The analysis yielded intriguing results pointing towards reduced expression of barrier related genes. However, none of the results are validated by an alternative approach such as qPCR. Similarly, analysis on SCFA pathways (Fig 3) was based on RNAseq data without any further validation.

2. Most conclusions are drawn from imaging data collected from immunohistochemistry, but there are two major concerns: (1) The images could benefit from more quantification. With single panels shown for most experimental conditions in the main figures, it is difficult to reliably evaluate the phenotypes. For example, the minimal and maximal length of continuous ZO-1 or Occludin signal, and the overall distribution of these values, can be used to demonstrate disruption of TJ. The thickness or total area can be other helpful parameters. (2) It is not specified in the method section the number of tissue sections analyzed per animal, and their anatomical locations. Maybe adding some data from the supplemental figures into the main figures would also help. Choroid plexus anatomy can also vary between anterior to posterior locations even in the same ventricle. In AD genetic models it is known that Ab deposition and pathology vary drastically even between brain hemispheres. These details are essential to incorporate into evaluating and interpreting the data.

3. It is worrying how the impact of propionate and butyrate can vary from model to model. In this study, it appeared the propionate had stronger rescue effect in the in vitro LPS model and in vivo microbiota removal model. However, butyrate had stronger impact on Ab pathology while propionate showed stronger induction of microglia phenotype. There is no explanation provided regarding the potential mechanisms. What are the mechanisms of action of these fatty acids and how are they anticipated to mediate their effects on target tissues? Do they interact with specific receptors on target cells that could be tracked in the present study? Some dose-dependent studies may help shed light on the effects. In addition, how are they transported to the blood-CSF barrier from gut? Are they present in the CSF? Are the CPE cells cells exposed to SCFA through their basal or apical surface?

4. The investigation in the AD model seems incomplete. In addition to absence of information on number of sections and anatomical locations as mentioned before, several additional questions emerge: (1) Ab pathology was evaluated across the entire brain but microglial phenotypes were analyzed in the hippocampus only. Is hippocampal pathology particularly decreased compared to the rest of the brain? Is the same microglial phenotype observed in other brain regions? (2) It is surprising to see such a robust reduction of Ab deposit with only 3 days of treatment. What is the hypothesis regarding such fast reduction of plaques? How are the plaques quantified? What is the area cutoff to define a plaque? Are there more microglia associated with plaques? Are there increased Ab signal within microglia, suggesting increased phagocytosis? What is the level of soluble Ab in CSF and ISF, or overall brain lysate? (3) Reactive microglia may cause additional damage. What is the synapse density, typically measured by synaptophysin-PSD95 co-localization, for example after 3 days? Are astrocytes activated at the same time? (4) Microglia are already at a very different state in AD mice vs. their wild-type controls. Are these microglial changes by SCFA only present in AD mice or also present in wild-type mice treated with these SCFAs? The current analysis is seems too preliminary to yield meaningful understanding of the changes in AD. As presented, it is unfortunately difficult to draw conclusions on propionate and butyrate functions in AD.

5. Overall, the statistics appear weak with a relatively small replicate numbers. Providing exact P values would also allow for better evaluation.

Referee #3:

Though increased BBB permeability in AD pathology has been well documented, blood-CSF barrier is not well studies. The current study is attempting to dissect the influence of gut microbiota on its barrier function. Here, Jie et al., show that gut microbiota deficiency enhances blood-CSF barrier permeability, associated with disorganised tight junction proteins (TJs). Recolonization with normal gut microbiota or SCFA rescues the defective blood-CSF barrier by improving TJ sub cellular distribution. Notably, vagotomy also disrupts blood-CSF barrier which was improved by SCFA.Employing a APP NL-G-F AD mice, an AD mouse model that display increased blood-CSF barrier permeability and decreased SCFA in feces, the authors found that SCFA administration improve TJs localization in the blood -CSF barrier. This treatment leads to Abeta burden reduction, accompanied with activated microglia. Overall, these findings are interesting, and the data fit with the main conclusion. Nevertheless, the observation does not represent a striking conceptual advancement that EMBO J. is seeking for.

Major concerns:
Especially, most of the data basically recapitulated previously published reports, lacking of innovative observation. For instance,

SCFAs activate microglia and regulate Abeta clearance, and they mediates BBB or Blood/CSF barriers. These were well known in the literature.

Minor concerns:
It is unclear, how vagus nerve resectioned by vagotomy and SCFAs crosstalk in blood/CSF barrier regulation. In the experiment, it remains unclear whether single side or two sides' vagus nerves were resected. If both sides of vagus nerves are transected, the animal still survived? If only on side is resected, how could the authors conclude "SCFA can bypass the vagus nerve and affect Blood-CSF barrier permeability"

** As a service to authors, EMBO Press provides authors with the possibility to transfer a manuscript that one journal cannot offer to publish to another EMBO publication or the open access journal Life Science Alliance launched in partnership between EMBO Press, Rockefeller University Press and Cold Spring Harbor Laboratory Press. The full manuscript and if applicable, reviewers' reports, are automatically sent to the receiving journal to allow for fast handling and a prompt decision on your manuscript. For more details of this service, and to transfer your manuscript please click on Link Not Available. **

Please do not share this URL as it will give anyone who clicks it access to your account.

Dear Editor, Dear Karin,

First of all, we would like to thank you and the reviewers for their helpful feedback and constructive criticism which will undoubtably benefit our manuscript positively. Importantly, we feel confident that we can meet most of the concerns that were raised by the reviewers. Based on this, we want to appeal the editorial decision to reject our manuscript EMBOJ-2022-111515-T.

To start off, we refute the major concern of referee #3 stating that our study lacks novelty. The current literature, as discussed in the introduction of the manuscript, solely focuses on the blood-brain barrier (BBB). In contrast, there is _*no data at all on the blood-cerebrospinal fluid *__*(CSF)*__*barrier, despite increasing evidence that it has an important and central role in periphery-to-brain communication*_as it is the main source of CSF production and controlled transport of molecules into the brain. Unfortunately, it *_remains a barrier that does not receive the attention and focus in the research community that it deserves and urgently needs_*. An important reason causing the science to lag behind on this topic is the challenging technical aspects that it entails. Being able to investigate the tiny choroid plexus tissue requires very specialized expertise and is very time consuming. One unavoidable but impactful issue is the small size of the tissue implicating that the number of analyses per choroid plexus (and thus per mouse) are strictly limited. Consequently, there are inevitable *_technical and also ethical limitations_* that should be kept in mind and have an impact on which experiments are realistic and/or reasonable to perform.

It is suggested that the reported data is not conclusive enough. We would address this by performing the following new experiments:

- We will validate the RNA-seq data by performing qPCR on the TOP differentially expressed (DE) genes for SPF /versus/ antibiotics-treated (AB) mice.

- While we will not perform RNA-seq data on germ-free (GF) mice, we aim to analyze the DE genes (mainly barrier function-related genes) on GF mice to be consistent with *Figure 2*.

- Forthe n number concerns, the experiments in *Figure 6* will be repeated to increase the n number. We believe that this might also address the remark of referee #2 on 'SCFA effect vary from model to model'. In *Figure 4*, we question the added value of increasing the n value as we detected significant changes. We want to stress that e.g.

the /in vitro/ experiment gave the same result in an independent experiment.

- As discussed in our manuscript, there are potential mechanisms by which SCFAs might act on the brain barrier, while it is not known whether the SCFAs enter the CSF/brain. We will perform metabolomics to address this question.

Other remarks we can already answer now are:

- We acknowledge that the manuscript could use some rewriting to make it more fluid and avoid the feeling we jump around from topic to topic. Other comments on text, figure legends and adding more statistical data will be addressed.

- Western blotting on choroid plexus tissue to measure tight junction (TJ) protein levels is not useful and will not grant added value to mechanistic insights. We claim that the TJs are less functional in localization, this probably not reflect in lower protein levels. As mentioned above, the amount of western blots on choroid plexus tissue is strictly limited due to its small size.

- The images are needed for visualizing the location of the TJs in the choroid plexus tissue. Quantifying the signal intensity is not a scientifically correct way to address this question. However, we can assess the continuity of TJs, as suggested by referee #2 (e.g. length of continuous TJ signal). In addition, we can also analyze the distribution density of TJ staining on choroid plexus epithelial cells. In this way, we will provide the quantification data for all the TJ staining figures.

- We used the /App^NL-G-F /^Alzheimer's disease mouse model to confirm data that is already known in literature, and then linking this to the innovative findings of our manuscript, i.e. the microbiota - blood-CSF barrier connection. Thus, we do not feel it necessary to phenotype this model in detail because this has already been extensively done in other studies. However, as suggested by the reviewer, we will extend our analyses to microglial changes, i.e. Aβ phagocytosis and synapse pruning which could give an explanation on the reduction of Aβ burden. Moreover, we will also analyze the effects of SCFAs on microglial functions in wild type mice.

- The celiac vagotomy is performed bilaterally as depicted in the supplementary material. We will mention this more specifically in the text to avoid confusion. Thus, our claim that SCFAs play a vital role in regulating blood-CSF barrier integrity independent of the vagus nerveis valid.

We hope to have persuaded you to grant us the opportunity for a full rebuttal. We would be able to submit a revised version in a time-period of around 2 months.

Thanks in advance for your time!

Sincerely,

Prof. dr. Roosmarijn Vandenbroucke

Associate Professor Ghent University | Group leader VIB

Ghent University - VIB | VIB-UGent Center for Inflammation Research

Technologiepark-Zwijnaarde 71, 9052 Ghent, BELGIUM | +32 9 331 37 30

This comment of the referee includes several separate remarks that we aim to address as much as possible:

- Related to the variation of the impact of propionate and butyrate in vitro versus in vivo: increasing the n value might solve this partially as several observations in the AD model are almost significant with the limited n value. Taking the outcome of these additional experiments and analyses into account, we will for sure address this in the manuscript and discussion. While we didn't include this in my previous e-mail, we are also considering to perform a new in vitro experiment including different propionate/butyrate doses which might contribute to the explanation of the observed differences.

- Related to the exact mechanism: by performing metabolomics on plasma and CSF/brain, we will be able to identify whether the SCFAs are able to reach the blood and brain. Also in our in vitro studies we will analyze whether the SCFAs are able to cross the blood-CSF barrier. This will reveal whether the CPE cells are exposed to the SCFA at both sides or not.

- Despite these experiments, we need to be realistic and we will not be able to identify which specific receptor(s) determine the SCFA responsiveness.

Kind regards,

Roos

Dear Roos,

Thank you for sending me your point-by-point response to the concerns raised by the referees. I have now had a chance to take a look at it and if you can address the concerns raised then I would like to consider a revised version.

Please note that I need strong support from the referees for consideration here.

When preparing your letter of response to the referees' comments, please bear in mind that this will form part of the Review Process File, and will therefore be available online to the community. For more details on our Transparent Editorial Process, please visit our website: https://www.embopress.org/page/journal/14602075/authorguide#transparentprocess

I thank you for the opportunity to consider your work for publication. I look forward to your revision.

with best wishes

Karin

Karin Dumstrei, PhD
Senior Editor
The EMBO Journal

Instructions for preparing your revised manuscript:

I have attached a PDF with helpful tips on how to prepare the revised version.

Guide For Authors: https://www.embopress.org/page/journal/14602075/authorguide

We realize that it is difficult to revise to a specific deadline. In the interest of protecting the conceptual advance provided by the work, we recommend a revision within 3 months (5th Sep 2022).

As a matter of policy, competing manuscripts published during this period will not negatively impact on our assessment of the conceptual advance presented by your study. However, we request that you contact the editor as soon as possible upon publication of any related work, to discuss how to proceed.

If you require more time to complete the revisions we can grant an extension.

Referee #1

In this paper, entitled, "Gut microbiota has a major impact.....", by J., Xie., et al, the authors have studied gut microbiota/gut metabolite effects on blood-CSF barrier (BCB) permeability. They report that mice lacking gut microbiota, display increased BCB associated with changes in the organization of tight junctions (TJs). The authors also report that gut microbiota or supplementation with short-chain fatty acids (SCFAs) restore the organization of TJs. These effects are still observed in "vagotomized" mice suggesting an n.vagus independent mechanism. Finally, the authors report that SCFA administration to *App*$^{NL\text{-}G\text{-}F}$ AD transgenic mice improves the BCB barrier as monitored by a reduction in Aβ burden and changes in microglial phenotype.

General comments.

The topic is clearly of interest and highly relevant to a broader scientific community. However, while positive about the topic and the well-written manuscript, the lack of controls reduces my enthusiasm somewhat

More specifically

1. In figure 1 they display the gene expression profile of SPF mice and AB treated mice but not GF mice? Why? There should be consistency across the figures with all groups presented in the same way across the figures. Please include the expression data from GF and align accordingly.

Although we agree that the inclusion of RNA-seq data of GF mice might be better aligned with Figure 2, we only performed RNA-seq on the choroid plexus of SPF and AB mice as displayed in Figure 1. We believe this is sufficient as a first step to investigate the impact of gut microbiota on the choroid plexus. To address the question of the reviewer, we performed qPCR to check several differential expression genes (DEGs) on all groups, namely SPF, AB, ABR, GF and GFR mice, as shown in **Figure EV2**. The text has been adapted accordingly in the revised manuscript as follows:

Page 6, line 143-152

> *"Lastly, we investigated the expression of RNA-seq-detected DEGs in choroid plexus of SPF, AB, ABR, germ-free (GF) and recolonized GF (GFR) via qPCR, focusing on the genes associated with barrier function. Consistent with the RNA-seq results, Cbln1 gene expression was significantly downregulated in AB mice compared to SPF mice, while all other tested DEGs showed showed only a trend towards (**Fig EV2**). In addition, this downregulation appeared to be suppressed to some extent upon gut microbiota reconstitution (ABR; **Fig EV2**). In GF mice, Fat2, Tmem252, Slc13a3, Slc6a13 and Aqp4 showed trends of downregulation similar to AB mice, but none of these genes showed upregulation upon gut microbiota recolonization (GFR; **Fig EV2**)."*

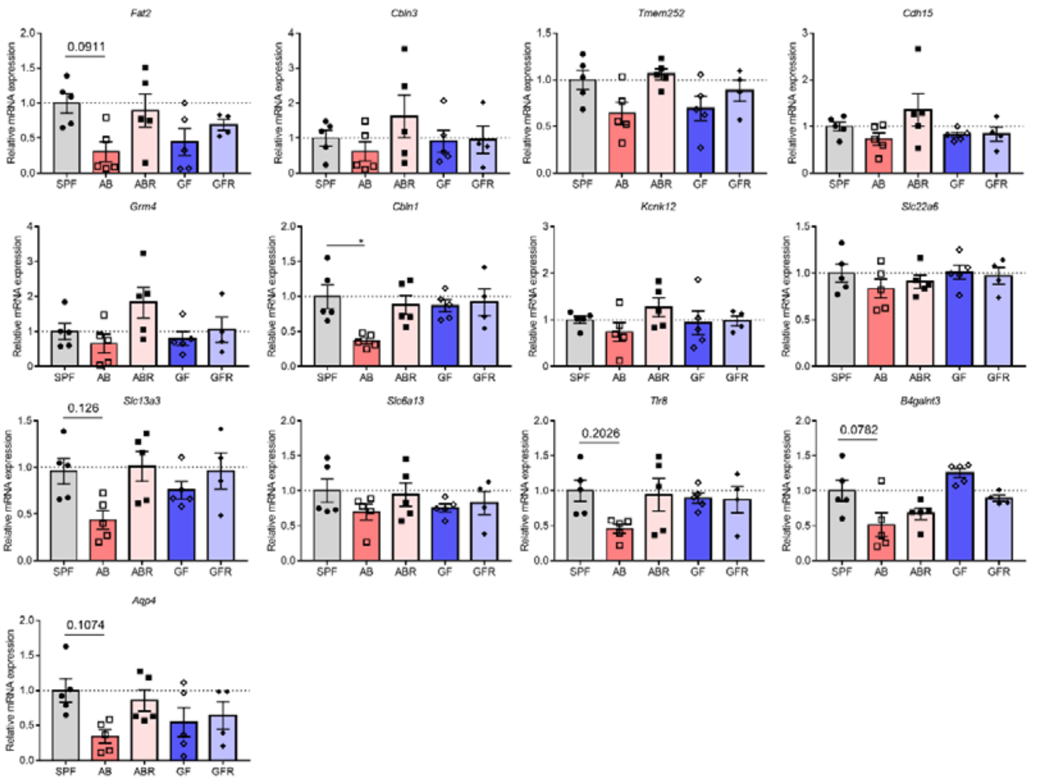

Figure EV2. qPCR analysis for barrier function-associated genes on choroid plexus of SPF, AB, ABR, GF and GFR mice. Bars represent mean ± SEM. Statistics were performed with one-way ANOVA Bonferroni's post hoc test for multiple comparisons. *$p < 0.05$. AB, antibiotics-treated; ABR, recolonized AB; GF, germ-free; GFR, recolonized GF; SPF, specific pathogen-free.

2. The lack of quantification of TJs WB blot data is surprising. The authors must include WB data in all the data sets monitoring TJs is displayed.

While WB data may provide information on changes in total protein level, changes in subcellular localization of the TJs can't be detected via this technology. Importantly, the latter plays the most important role in TJ functionality. In addition, we want to stress that due to its very small size, performing WB on choroid plexus tissue is very challenging and requires often pooling of different mice, while we don't believe these analyses have much added value to the conclusions of our study.

As an alternative and to address the question of the reviewer, we performed quantification of the TJ immunostainings which allowed to quantify both the subcellular location and expression of the TJ proteins. First, we performed Ridge Detection-based analysis using Image J to examine the distribution of continuous and maximal TJ length, which is indicative of the subcellular localization of TJs. In addition, as shown below, we quantified the TJ expression by dividing the area of TJ signal by the area of epithelial nuclei, which reflects the total protein expression level per cell and serves as an alternative for WB. This was applied to all conditions (**Figure 2A-D**, **4A-E**, **5A-D**, **5H-K**, **6B-E**, **EV3A-D** and **Appendix Figure S7A-D** in the revised MS). As an example, you can find **Figure 2B-D** below.

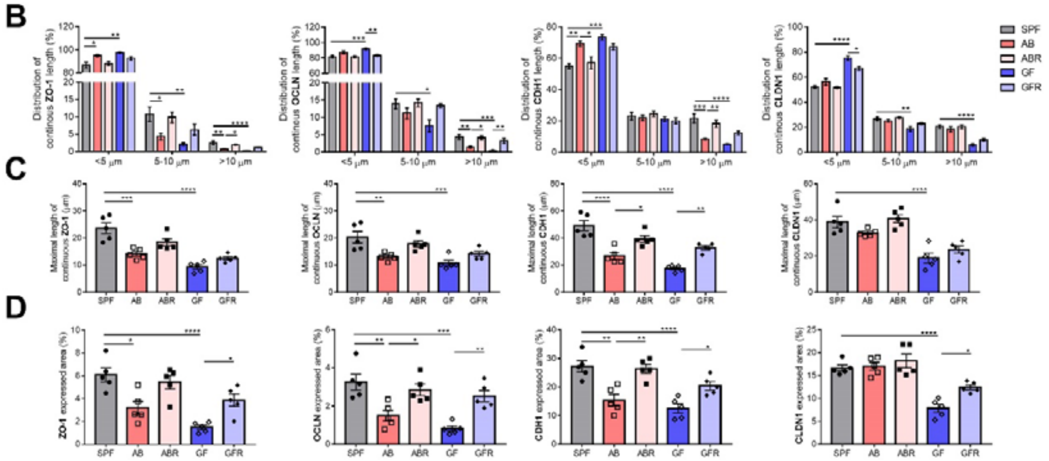

Figure 2. Blood-CSF barrier integrity in mice with different gut microbiota composition. (**B**-**D**) Quantification of continuous length (**B**), maximal length (**C**), and expressed area (**D**) of the TJ immunostainings displayed in (**A**). Bars represent mean ± SEM. Statistics were performed with one-way ANOVA Bonferroni's post hoc test for multiple comparisons. *p < 0.05, **p < 0.01, ***p < 0.001, ****p < 0.0001. AB, antibiotics-treated; ABR, recolonized AB; CSF, cerebrospinal fluid; GF, germ-free; GFR, recolonized GF; SPF, specific pathogen-free; TJ, tight junction.

3. The use of IgG quantification provides information about the degree of leakiness. Please increase the n numbers in Fig 4 and Fig 6d to n=5. N=3 is not sufficient

In the revised manuscript, we increased the n numbers to minimally 5 in **Figure 4L-M** and **6F-G**. The resulting data of the IgG measurements are shown below.

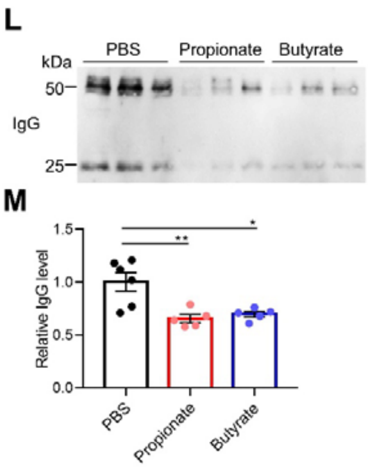

Figure 4. The effect of SCFAs on blood-CSF barrier integrity *in vitro* and *in vivo*. (**L**) Representative western blot showing the IgG levels in CSF of sodium propionate or sodium butyrate-treated mice with disrupted gut microbiota via antibiotics treatment. (**M**) Quantitative analysis of IgG level via western blot in (**L**). Bars represent mean ± SEM. Statistics were performed with one-way ANOVA Bonferroni's post hoc test for multiple comparisons. *p < 0.05, **p < 0.01. CSF, cerebrospinal fluid; SCFAs, short-chain fatty acids.

F

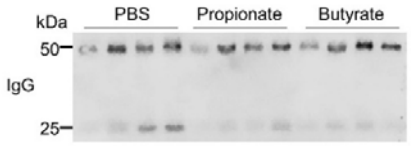

G

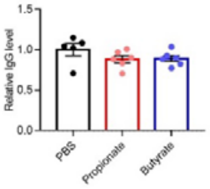

Figure 6. Effects of SCFAs on blood-CSF integrity and Aβ plaques in *App*$^{NL\text{-}G\text{-}F}$ mice. (**F**) Representative western blot showing the IgG levels in CSF of sodium propionate or sodium butyrate-treated mice with disrupted gut microbiota via antibiotics treatment. (**G**) Quantitative analysis of IgG level via western blot in (F). Bars represent mean ± SEM. Statistics were performed with one-way ANOVA Bonferroni's post hoc test for multiple comparisons. CSF, cerebrospinal fluid; SCFAs, short-chain fatty acids.

4. It would also be advisable if the authors could consider including another tracer to further document changes in BCB without SCFA and SPF vs GF and AB treated mice

Based on the suggestion of the reviewer, we tested functional leakage of the blood-CSF barrier and BBB using 4 kDa FITC-dextran tracer in the different settings and displayed in **Figure 2, 4**, **6**, and **EV3** and **Appendix Figure S7** (as shown below).

G

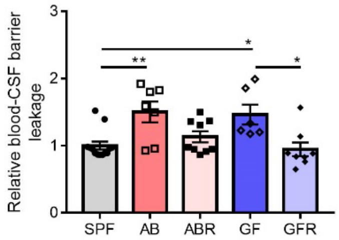

Figure 2. Blood-CSF barrier integrity in mice with different gut microbiota composition. (**G**) Assessment of the blood-CSF barrier permeability to 4 kDa FITC-dextran in SPF, AB, ABR, GF and GFR mice. Bars represent mean ± SEM. Statistics were performed with one-way ANOVA Bonferroni's post hoc test for multiple comparisons. *$p < 0.05$, **$p < 0.01$. AB, antibiotics-treated; ABR, recolonized AB; CSF, cerebrospinal fluid; GF, germ-free; GFR, recolonized GF; SPF, specific pathogen-free.

K

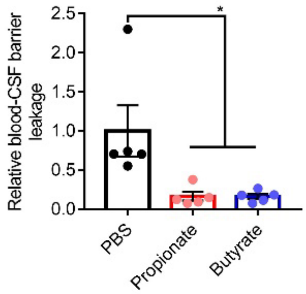

Figure 4. The effect of SCFAs on blood-CSF barrier integrity *in vitro* and *in vivo*. (**K**) Assessment of the blood-CSF barrier permeability to 4 kDa FITC-dextran in sodium propionate or sodium butyrate-treated mice with disrupted gut microbiota via antibiotics treatment. Bars represent mean ± SEM. Statistics were performed with one-way ANOVA Bonferroni's post hoc test for multiple comparisons. *$p < 0.05$. CSF, cerebrospinal fluid; SCFAs, short-chain fatty acids.

H

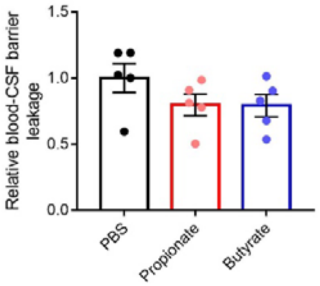

Figure 6. Effects of SCFAs on blood-CSF barrier integrity and Aβ plaques in *App*$^{NL\text{-}G\text{-}F}$ mice. (**H**) Assessment of the blood-CSF barrier permeability to 4 kDa FITC-dextran in sodium propionate or sodium butyrate-treated *App*$^{NL\text{-}G\text{-}F}$ mice. Bars represent mean ± SEM. Statistics were performed with one-way ANOVA Bonferroni's post hoc test for multiple comparisons. CSF, cerebrospinal fluid; SCFAs, short-chain fatty acids. CSF, cerebrospinal fluid; SCFAs, short-chain fatty acids.

E

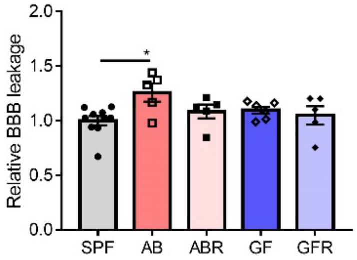

Figure EV3. BBB integrity in mice with different gut microbiota composition. (**E**) Assessment of the BBB permeability to 4 kDa FITC-dextran in SPF, AB, ABR, GF and GFR mice. Bars represent mean ± SEM. Statistics were performed with one-way ANOVA Bonferroni's post hoc test for multiple comparisons. *$p < 0.05$. AB, antibiotics-treated; ABR, recolonized AB; BBB, blood-brain barrier; GF, germ-free; GFR, recolonized GF; SPF, specific pathogen-free.

E

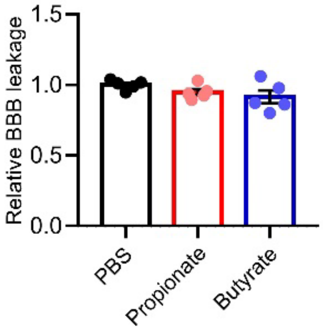

Appendix Figure S7. Effects of SCFAs on BBB integrity in *App*$^{NL\text{-}G\text{-}F}$ mice. (**E**) Assessment of the BBB permeability to 4 kDa FITC-dextran sodium propionate or sodium butyrate-treated *App*$^{NL\text{-}G\text{-}F}$ mice. Bars represent mean ± SEM. Statistics were performed with one-way ANOVA Bonferroni's post hoc test for multiple comparisons. BBB, blood-brain barrier; SCFAs, short-chain fatty acids.

5. The microscopic pictures in Figure 7 are overall of less good quality. Please provide higher resolution photos

We now provided images with higher quality in **Figure 7**. Also, the raw Tif images have been submitted to BioImage Archive with accession number S-BIAD609 (https://www.ebi.ac.uk/biostudies/bioimages/studies/S-BIAD609).

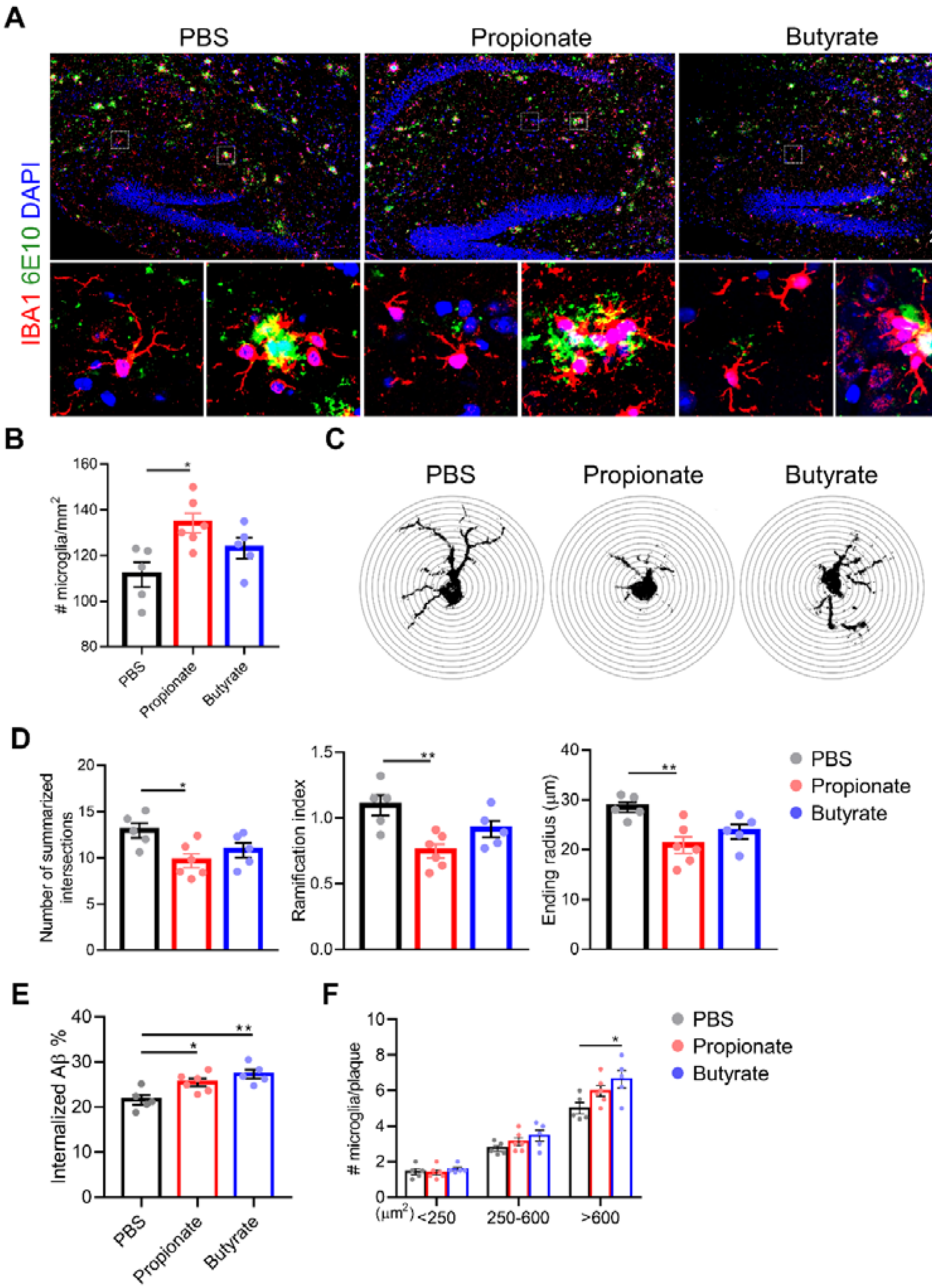

Figure 7. Effects of SCFAs on microglial proliferation and activation in *App*$^{NL-G-F}$ mice. (**A**) Representative images of immunostainings for IBA1 and 6E10 in hippocampus of sodium propionate or sodium butyrate-treated *App*$^{NL-G-F}$ mice. Scale bar: 200 μm. (**B**) The number of IBA1^{+} microglia in hippocampus. (**C**) Representative image for Sholl analysis of non-Aβ associated microglia in the hippocampus from the IBA1-stained image in (**A**). The interval of the concentric circles is 2 μm. (**D**) Quantification of summarized intersects, ramification index and ending radius by Sholl analysis from immunostained IBA1 signals. (**E**) Quantification of the percentage of overlay area of microglia and Aβ plaque. (**F**) Plaques were divided into small (< 250 μm^2), medium (250-600 μm^2), and large (> 600 μm^2), and the number of microglia per plaque was quantified. Bars represent mean ± SEM. Statistics were performed with one-way ANOVA Bonferroni's post hoc test for multiple comparisons. *p < 0.05, **p < 0.01. SCFAs, short-chain fatty acids.

Referee #2

In this study, Xie et al. compared mice with vs. without gut microbiota to demonstrate that removal of gut microbiota negatively impacts the choroid plexus epithelial (CPE) tight junction distribution and barrier functions. They further showed that SCFA produced by gut microbes, specifically propionate and butyrate, can rescue tight junction damage in an in vitro LPS model. This effect appears to be independent of vagus nerve. Finally, they studied a genetic model of Alzheimer's disease (*App*$^{NL\text{-}G\text{-}F}$) and where they suggest that propionate and butyrate treatment alleviated Ab pathology and altered microglia phenotypes.

The very interesting and strongest part of this study is presented in the first half of the paper - demonstration that the blood-CSF barrier protein expression and localization depends on the gut microbiome is striking. 1. However, overall, the study jumps around from topic to topic, without delving sufficiently deep into any single subject/mechanisms involved, 2. and draws strong conclusions based on a limited number of experiments and small sample sizes. 3. The vagus nerve is an appealing topic, but it doesn't add very much to the present study since it is unlikely to be involved. 4. The last figure with the AD data seems preliminary and could be taken out and developed into a separate study. Below are more details about concerns regarding experimental design and data interpretation:

1. Thank you for your positive comment related to the content of our manuscript. Related to the comment on 'jumping around', we especially rewrote the section "**SCFAs reduce loss of blood-CSF barrier integrity and AD pathology in *App*$^{NL\text{-}G\text{-}F}$ mice**" in the revised manuscript to make it more fluid. This part also contains new data based on the other suggestions. The discussion has also been adapted accordingly.

Page 9-11, line 280-348

*"Numerous studies suggest that disruption of brain barriers is likely involved in the pathogenesis of several neurological diseases, including AD (Ueno et al., 2016). In addition, SCFAs, as important mediators of gut-brain interactions, have been implicated in AD development and progression, but it is not well known which SCFAs mechanism(s) are important in AD (Qian et al., 2022). Based on our results that recognize SCFAs as the key mediators of the gut microbiota's effect on blood-CSF barrier integrity, we hypothesized that SCFAs might revert AD pathology by tightening brain barrier. Here, we used App$^{NL\text{-}G\text{-}F}$ mice, an AD mouse model in which we previously observed increased blood-CSF barrier permeability (Xie et al., 2021) and a decreased ratio of SCFA generating bacteria in faeces has been reported (Kaur et al., 2020). In agreement with the latter, we observed lower levels of propionate and butyrate in the faeces of App$^{NL\text{-}G\text{-}F}$ mice compared to their wild type counterparts (**Appendix Figure S6**). Next, we evaluated the changes of BBB and blood-CSF barrier in App$^{NL\text{-}G\text{-}F}$ mice given sodium propionate or sodium butyrate by oral gavage for 3 days (**Fig 6A**). Compared to App$^{NL\text{-}G\text{-}F}$ control mice, propionate and butyrate-treated App$^{NL\text{-}G\text{-}F}$ mice showed significant increased TJ expression and slightly improved TJ*

*subcellular localization, determined for OCLN, CDH1, ZO-1 and CLDN1 via quantification of 'expressed area' and 'distribution of continuous TJ protein length and the maximal length of continuous TJ protein' on immunostainings, respectively (**Fig 6B-E**). In case of BBB, both expression and localization of the TJs were only slightly improved (**Appendix Fig S7A-D**). Functional analysis of the blood-CSF barrier integrity via IgG CSF levels and 4 kDa FITC-dextran leakage showed that both butyrate and propionate treatment caused only a trend towards increased barrier tightening (**Fig 6F-H**). BBB functionality in the $App^{NL\text{-}G\text{-}F}$ mice was barely affected in response to SCFA treatment (**Appendix Fig S7E**).*

*Microglia are the most prominent immune cells of the CNS and they are known to play an important role in the functionality of the BBB and in β-amyloid (Aβ) clearance (Haruwaka et al., 2019, Tejera et al., 2019). First, we investigated whether the barrier-restoring effect of SCFAs could inhibit Aβ aggregation. Strikingly, both propionate and butyrate treatments resulted in a significant decrease of Aβ accumulation in the brain of $App^{NL\text{-}G\text{-}F}$ mice which was reflected both in total Aβ plaque area and the number of Aβ plaques (**Fig 6I, J**). Less tight brain barriers may increase peripheral factors (e.g. cytokines and immune cells) infiltration to the CNS and induce neuroinflammation, thereby affecting microglial functions (Takata, Nakagawa et al., 2021). Next, we evaluated whether the barrier-restoring effect of SCFAs would be beneficial in enhancing the ability of microglia to phagocytose Aβ. A significantly increased number of microglia was observed in the olfactory bulb of propionate and butyrate-treated $App^{NL\text{-}G\text{-}F}$ mice, while only propionate treatment induced a pronounced increase in the number of microglia in the hippocampus and cerebellum. In cortex and striatum, the number of microglia did not show a difference after SCFAs treatment (**Fig 7A, B** and **Fig EV5A, B**). The detailed Sholl analysis of non-Aβ plaque-associated microglia showed that the sum of intersections, ramification index, and ending radius decreased significantly in propionate-treated $App^{NL\text{-}G\text{-}F}$ mice (**Fig 7C, D**), indicating a more activated microglial phenotype. Similarly, the sodium butyrate treatment showed the same trends in microglial proliferation and activation but the changes were not significant (**Fig 7A-D**). Additionally, we also observed a markedly increase in microglial internalized Aβ in both propionate and butyrate-treated $App^{NL\text{-}G\text{-}F}$ mice, but only butyrate treatment showed a significant increase in the number of microglia adjacent to the large plaques (> 600 μm^2) in $App^{NL\text{-}G\text{-}F}$ mice (**Fig 7E, F**). However, when administering the same doses of propionate and butyrate in WT mice, we didn't observe difference in microglial proliferation or activation (**Appendix Fig S8A-D**).*

*Of note, microglial activation may lead to the conversion of resting astrocytes to reactive astrocytes and neuronal dysfunction (Leng & Edison, 2020). First, we examined the effects of propionate and butyrate on proliferation and activation of astrocyte in WT and $App^{NL\text{-}G\text{-}F}$ mice (**Appendix Figure S9A, B** and **Appendix Figure S10A, B**). GFAP immunostaining showed no significant changes in astrocyte morphology nor number in WT and $App^{NL\text{-}G\text{-}F}$ mice after propionate and*

*butyrate treatments (**Appendix Figure S9A, B** and **Appendix Figure S10A, B**). Next, we analyzed the effects of propionate and butyrate on synaptic properties in App$^{NL\text{-}G\text{-}F}$ mice (**Appendix Figure S11A-D**). High-resolution images of co-immunostaining of presynaptic protein synaptophysin (SYP) and postsynaptic density protein 95 (PSD-95) showed similar number of double-positive synaptic puncta in the area of the cortex and hippocampus of SCFA-treated App$^{NL\text{-}G\text{-}F}$ mice compared to App$^{NL\text{-}G\text{-}F}$ controls (**Appendix Figure S11A, B**). Moreover, co-immunostaining of IBA1 and PSD-95 did not show excessive synaptic pruning by microglia and even a downregulation trend observed (**Appendix Figure S11C, D**).”*

<u>Page 14-15, line 449-497</u>

“*This slight improvement of the barrier integrity in the App$^{NL\text{-}G\text{-}F}$ mice upon SCFA treatment was however associated with a significant reduction in Aβ accumulation in the brain, which is in concordance with previous reports showing that administration of sodium butyrate can decrease brain Aβ level and improve cognitive memory performance in a transgenic mouse model of AD (Fernando et al., 2020). Nevertheless, we cannot exclude the possibility that SCFAs may affect microglial phenotype and Aβ pathology through other mechanisms than brain barrier tightening. In this regard, both propionate and butyrate were shown to induce microglial activation resulting in reduced Aβ accumulation by increasing Aβ phagocytosis, clearance, and degradation (Solito & Sastre, 2012). Importantly, we couldn’t show the presence of the administered SCFAs in the brain, which is needed to exert direct effects on the microglia, while we did show that SCFA in vitro strengthen the blood-CSF barrier TJs directly. However, SCFAs may also indirectly affect microglial functions by mediating inflammation, as butyrate is a strong anti-inflammatory mediator, while other SCFAs, such as propionate enhance the inflammatory responses (Huuskonen et al., 2004). The inflammatory effects of SCFAs may further impact microglial activation and then microglial Aβ phagocytosis. In this study, we found that SCFA treatment increased microglial Aβ internalization and the number of microglia adjacent to large Aβ plaques in App$^{NL\text{-}G\text{-}F}$ mice. Notably, propionate induces higher levels of microglial activation than butyrate, and this state of hyperactivation may reduce their Aβ phagocytic capacity, as we demonstrated in our previous study (Xie et al., 2021). In addition to the inflammatory effects, SCFAs can also regulate microglia maturation and function, possibly through upregulation of the ApoE-TREM2 pathway and increasing histone acetylation (Bauer et al., 2022, Colombo et al., 2021, Erny et al., 2021, Erny et al., 2015); thereby affecting the ability of microglia in the internalization and degradation of Aβ (Lee & Landreth, 2010). The differences in propionate and butyrate in affecting these pathways may also contribute to their differential microglial Aβ phagocytosis. In contrast to our results, a recent study reported that treatment with SCFA mixtures (sodium acetate, sodium propionate and sodium butyrate) increased the Aβ plaque load in GF AD mice to levels of normal SPF mice and even further exacerbate Aβ*

burden in AD mice (Colombo et al., 2021). The varied outcomes could be due to the different experimental setups, such as mouse model, age at SCFA treatment, SCFA dosage, and treatment route. For example, excessive propionate intake in healthy humans has been reported to increase the risk for AD (Killingsworth et al., 2020). In our study, we observed that SCFA treatment increased the number of microglia and induced microglial activation in the hippocampus of App$^{NL\text{-}G\text{-}F}$ mice but not WT mice, indicating that under physiological condition, supplementation of SCFAs has no pronounced effect on microglial proliferation and activation; however, under pathological condition, SCFA treatment may promote a reduction of Aβ burden in the brain by increasing the number of microglia and inducing microglial activation. The microglia may benefit from the barrier-restoring effects of SCFAs, which limit the leakage of neuroinflammation-inducing peripheral factors into the brain, thereby preventing microglial hyperactivation and the maintenance of their functionality. Thus, further studies are needed to evaluate the beneficial potential of individual SCFAs on AD and develop the rational for personalized treatment strategies. In addition, previous studies have shown that microglial activation may lead to the conversion of resting astrocytes to reactive astrocytes and neuronal dysfunction such as excessive synaptic pruning (Azevedo et al., 2013, Leng & Edison, 2020, Litvinchuk et al., 2018), but we did not observe changes in astrocyte proliferation and activation, nor synaptic loss in SCFA-treated App$^{NL\text{-}G\text{-}F}$ mice. These results indicate that SCFA-induced increased phagocytic activity and proliferation of the microglia has no effect on astrocyte activation and synaptic pruning."

2. Related to the limited number of experiments and small sample sizes, we included more experiments and analyses to further investigating the effects of the presence of gut microbiota and SCFAs on the blood-CSF barrier and AD pathology. First, we performed three new experiments to examine the dose effect of SCFA on blood-CSF barrier integrity and the effect of SCFA on activation of glial cells in wildtype mice and synaptic pruning in *App*$^{NL\text{-}G\text{-}F}$ mice. Please check the response to the comment 7.2 and 8.3-8.5. Second, we included different quantifications (i.e. continuous length, maximal length and expressed area) of TJ immunostaining in **Fig 2A-D**, **4A-E**, **5A-D**, **5H-K**, **6B-E**, **EV3A-D** and **Appendix Figure S7A-D** in the revised manuscript, enabling visualization of biological replicates of these experiments. In these quantifications, Ridge Detection-based analysis in Image J were used to examine the continuous length and maximal length of TJs, which is indicative of the subcellular localization of TJs. The TJ expression was quantified by dividing the area of TJ signal by the area of epithelial nuclei, which reflects the total protein expression level per cell. The corresponding text in figure, methods, results, and discussion have been altered appropriately. Lastly, we increased the n numbers to minimally 5 in **Fig 4**, **6** and **7**.

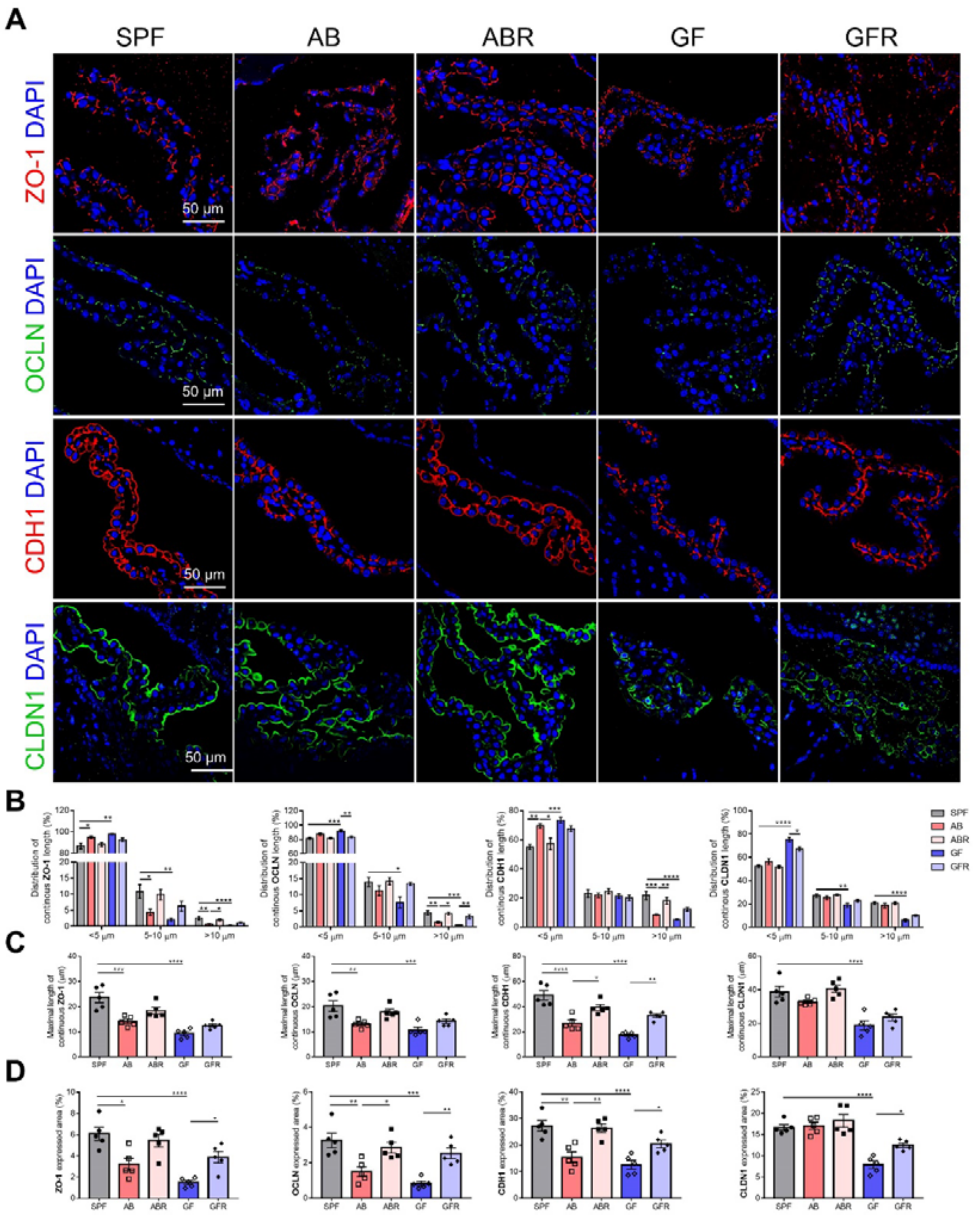

Figure 2. Blood-CSF barrier integrity in mice with different gut microbiota composition. (**A**) Representative images of immunostainings for ZO-1, OCLN, CDH1 and CLDN1 in choroid plexus of SPF, AB, ABR, GF and GFR mice. Scale bar: 50 µm. (**B**-**D**) Quantification of continuous length (**B**), maximal length (**C**), and expressed area (**D**) of the TJ immunostainings displayed in (**A**). Bars represent mean ± SEM. Statistics were performed with one-way ANOVA Bonferroni's post hoc test for multiple comparisons. *p < 0.05, **p < 0.01, ***p < 0.001, ****p < 0.0001. AB, antibiotics-treated; ABR, recolonized AB; CSF, cerebrospinal fluid; GF, germ-free; GFR, recolonized GF; SPF, specific pathogen-free; TJ, tight junction.

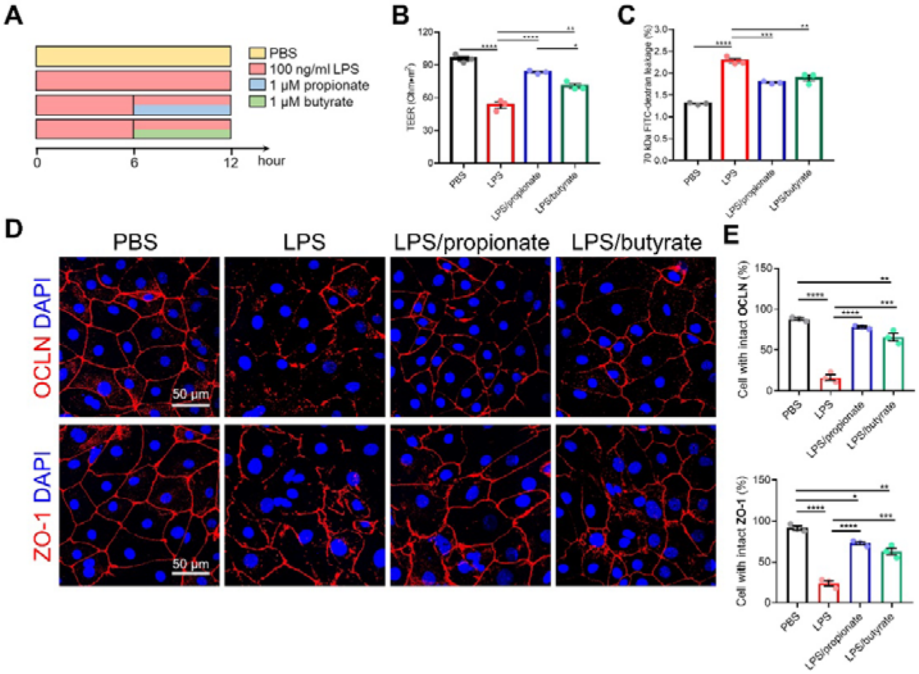

Figure 4. The effect of SCFAs on blood-CSF barrier integrity *in vitro* and *in vivo*. (**A**) Schematic representation of the experimental conditions. (**B**) TEER and (**C**) assessment of the 70 kDa FITC-dextran paracellular permeability of primary choroid plexus epithelial (CPE) cells following treatment for 6 h with 1 μM sodium propionate or 1 μM sodium butyrate, with 100 ng/ml LPS stimulation (n=3 duplicates). (**D**) Representative images of immunostainings for ZO-1 and OCLN in primary CPE cells following treatment for 6 h with 1 μM sodium propionate or 1 μM sodium butyrate, with 100 ng/ml LPS stimulation. Scale bar: 50 μm. (**E**) Quantification of primary CPE cells with intact TJ immunostainings in (**D**). Bars represent mean ± SEM. Statistics were performed with one-way ANOVA Bonferroni's post hoc test for multiple comparisons. *$p < 0.05$, **$p < 0.01$, ***$p < 0.001$, ****$p < 0.0001$. CSF, cerebrospinal fluid; LPS, lipopolysaccharides; SCFAs, short-chain fatty acids; TEER, trans-epithelial electrical resistance; TJ, tight junction.

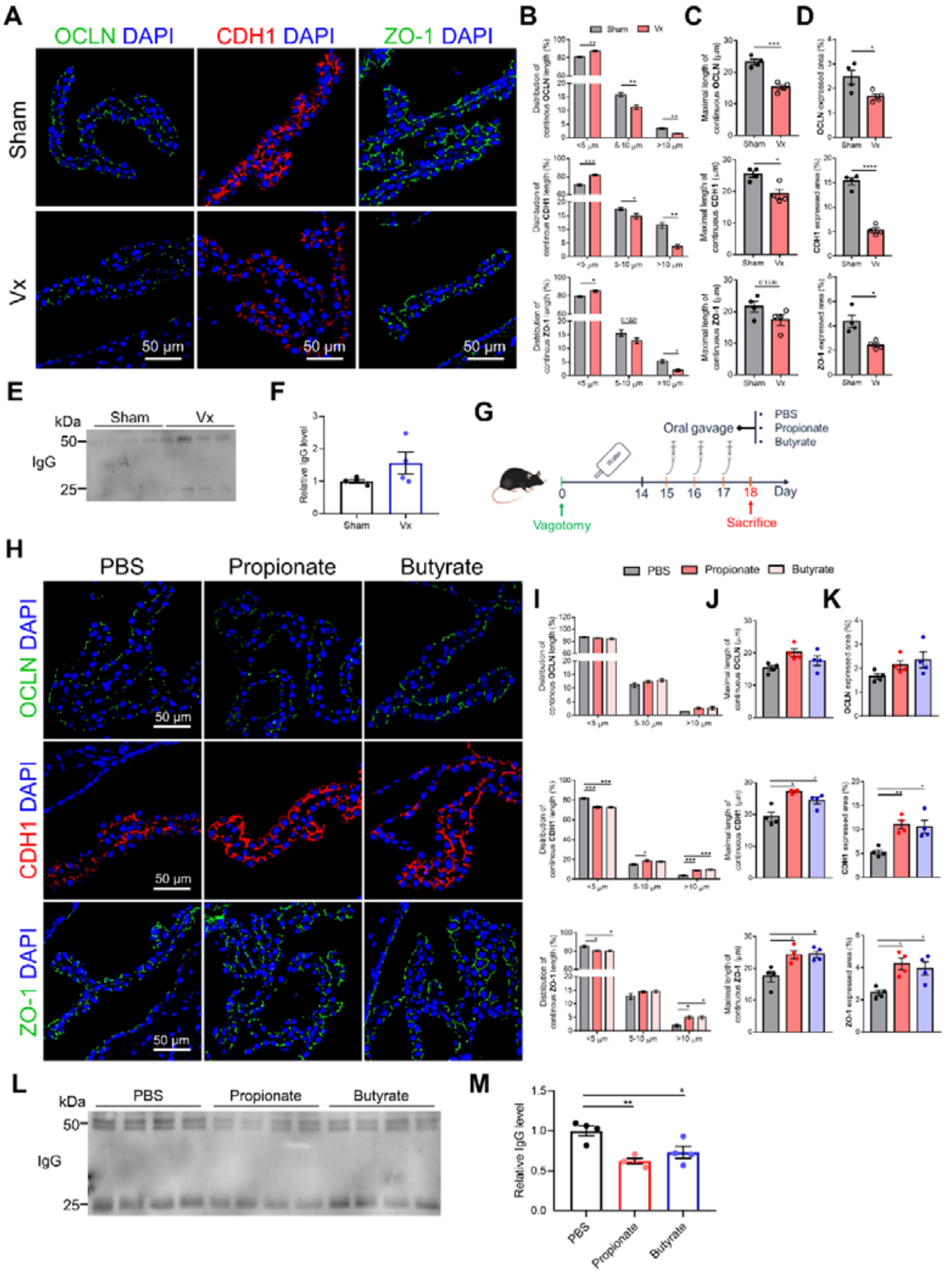

Figure 5. The role of the vagus nerve in regulating the blood-CSF barrier integrity. (**A**) Representative images of immunostainings for OCLN, CDH1 and ZO-1 in choroid plexus of sham and vagotomized mice. Scale bar: 50 μm. (**B**-**D**) Quantification of continuous length (B), maximal length (**C**), and expressed area (**D**) of TJ immunostainings in (**A**). (**E**) Western blot of IgG in CSF. (**F**) Quantitative analysis of IgG level. (**G**) Schematic representation of the experimental groups. (**H**) Representative images of immunostainings for OCLN, CDH1 and ZO-1 in choroid plexus of sodium propionate or sodium butyrate-treated vagotomized mice. Scale bar: 50 μm. (**I**-**K**) Quantification of continuous length (**I**), maximal length (**J**), and expressed area (**K**) of TJ immunostainings in (**H**). (**L**) Representative western blot showing the IgG levels in CSF of sodium propionate or sodium butyrate-treated vagotomized mice. (**M**) Quantitative analysis of IgG level in CSF via western blot in (**L**). Bars represent mean ± SEM.

Statistics were performed with one-way ANOVA Bonferroni's post hoc test for multiple comparisons. *p < 0.05, **p < 0.01. CSF, cerebrospinal fluid; TJ, tight junction.

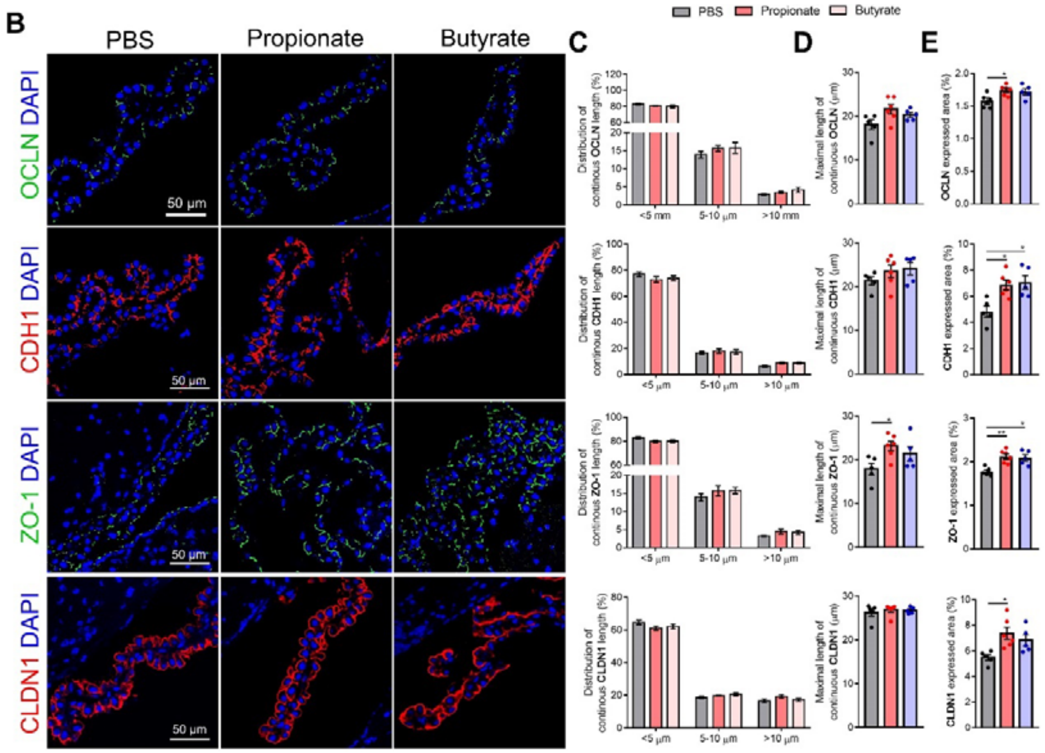

Figure 6. Effects of SCFAs on blood-CSF barrier integrity and Aβ plaques in *App*$^{NL\text{-}G\text{-}F}$ mice. (**B**) Representative images of immunostainings for OCLN, CDH1, ZO-1 and CLDN1 in choroid plexus of sodium propionate or sodium butyrate-treated *App*$^{NL\text{-}G\text{-}F}$ mice. Scale bar: 50 μm. (**C-E**) D Quantification of continuous length (**C**), maximal length (**D**), and expressed area (**E**) of the TJ immunostainings displayed in (**B**). Bars represent mean ± SEM. Statistics were performed with one-way ANOVA Bonferroni's post hoc test for multiple comparisons. *p < 0.05, **p < 0.01. CSF, cerebrospinal fluid; SCFAs, short-chain fatty acids; TJ, tight junction.

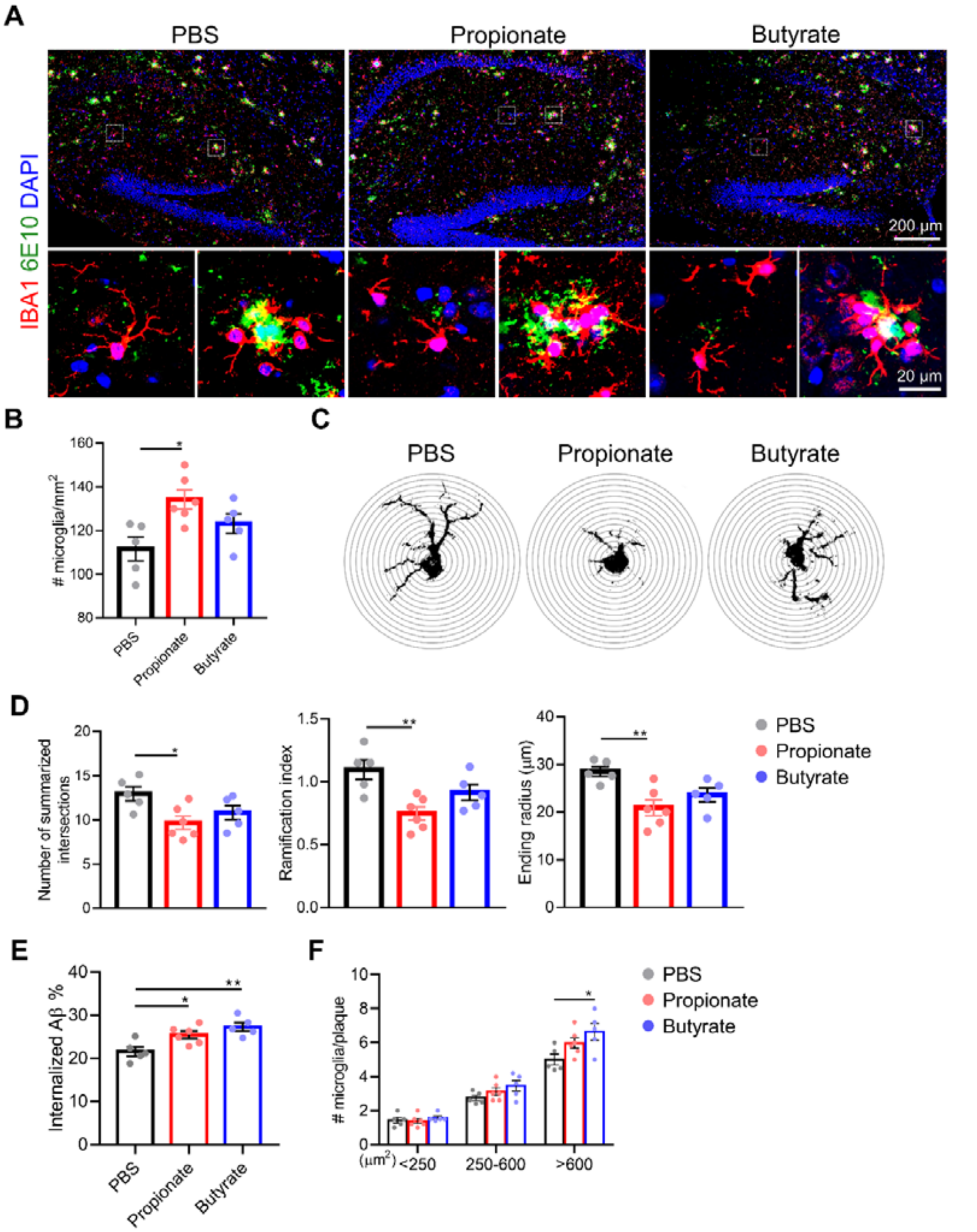

Figure 7. Effects of SCFAs on microglial proliferation and activation in *App*$^{NL\text{-}G\text{-}F}$ mice. (**A**) Representative images of immunostainings for IBA1 and 6E10 in hippocampus of sodium propionate or sodium butyrate-treated *App*$^{NL\text{-}G\text{-}F}$ mice. Scale bar: 200 μm. (**B**) The number of IBA1^{+} microglia in hippocampus. (**C**) Representative image for Sholl analysis of non-Aβ associated microglia in the hippocampus from the IBA1-stained image in (**A**). The interval of the concentric circles is 2 μm. (**D**) Quantification of summarized intersects, ramification index and ending radius by Sholl analysis from immunostained IBA1 signals. (**E**) Quantification of the percentage of overlay area of microglia and Aβ plaque. (**F**) Plaques were divided into small (< 250 μm^2), medium (250-600 μm^2), and large (> 600 μm^2), and the number of microglia per plaque was quantified. Bars represent mean ± SEM. Statistics were

performed with one-way ANOVA Bonferroni's post hoc test for multiple comparisons. *$p < 0.05$, **$p < 0.01$. SCFAs, short-chain fatty acids.

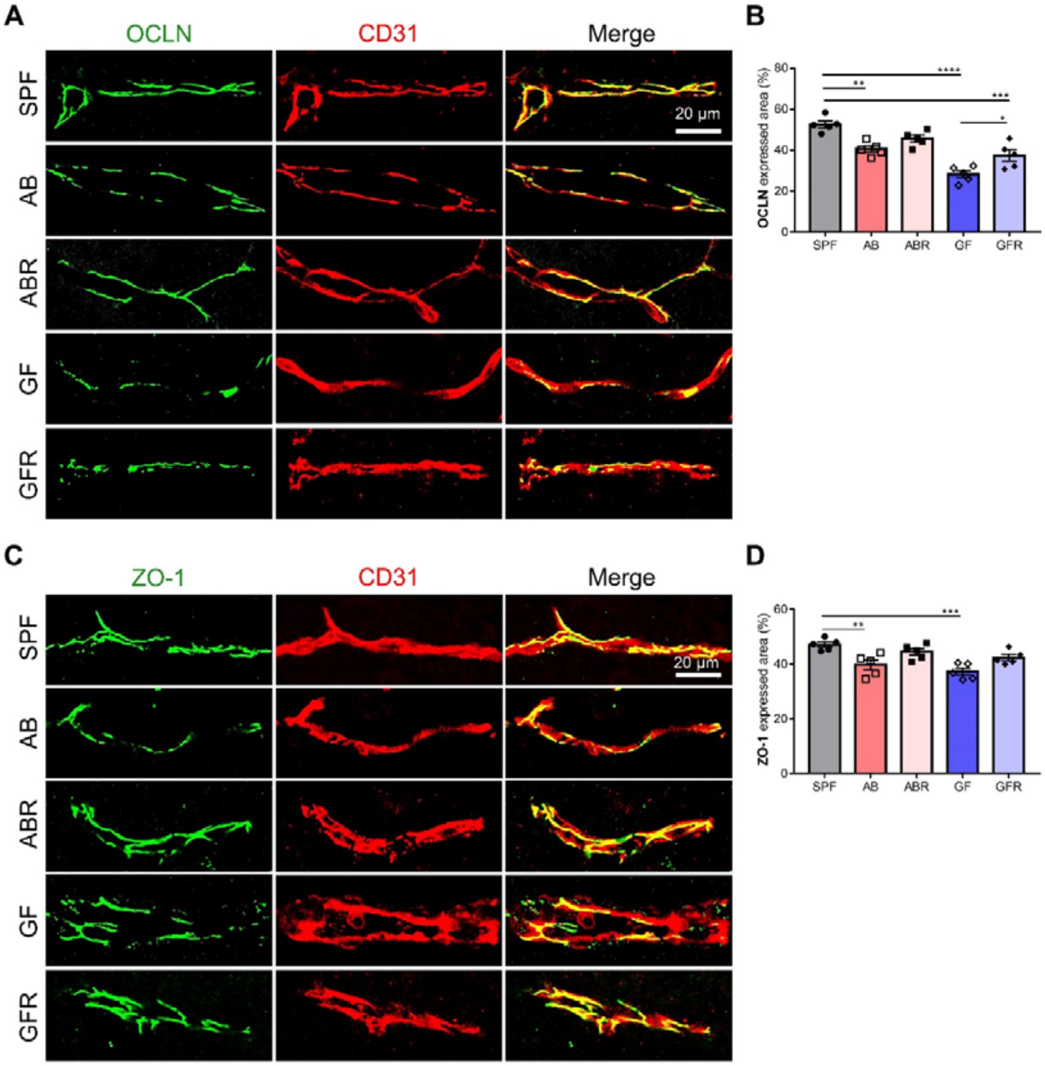

Figure EV3. BBB integrity in mice with different gut microbiota composition. (**A**) Representative images of immunostaining for OCLN and CD31 in cortex. Scale bar: 20 µm. (**B**) The percentage of expressed area of OCLN immunostaining displayed in (**A**). (**C**) Representative images of immunostaining for ZO-1 and CD31 in cortex. Scale bar: 20 µm. (**D**) The percentage of expressed area of ZO-1 immunostaining displayed in (**C**). Bars represent mean ± SEM. Statistics were performed with one-way ANOVA Bonferroni's post hoc test for multiple comparisons. *$p < 0.05$, **$p < 0.01$, ***$p < 0.001$. AB, antibiotics-treated; ABR, recolonized AB; BBB, blood-brain barrier; GF, germ-free; GFR, recolonized GF; SPF, specific pathogen-free.

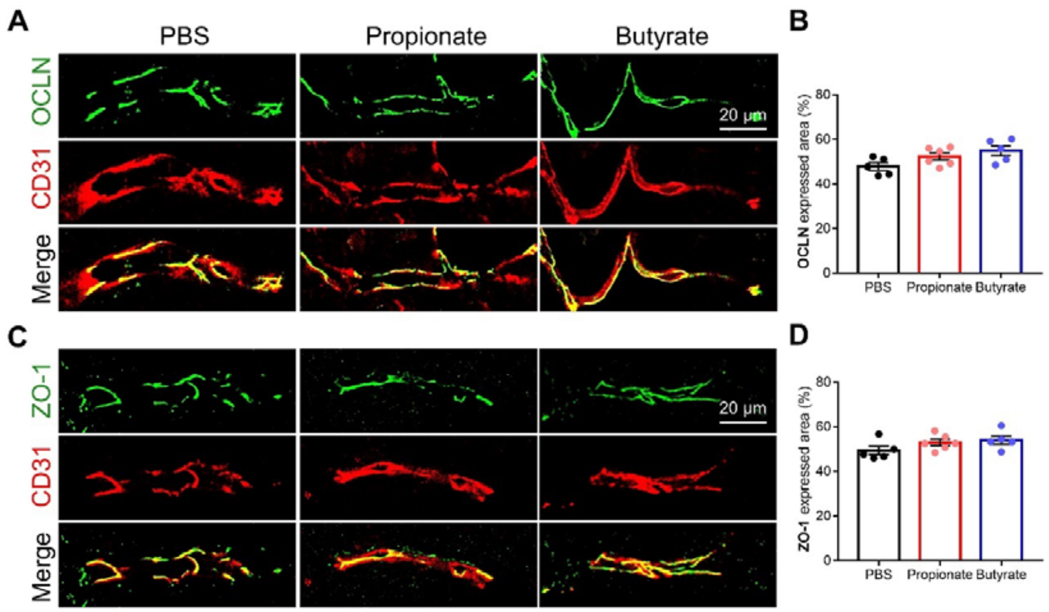

Appendix Figure S7. Effects of SCFAs on BBB integrity in *App*$^{NL\text{-}G\text{-}F}$ mice. (**A**) Representative images of immunostaining for OCLN and CD31 in cortex. Scale bar: 20 µm. (**B**) The percentage of expressed area of OCLN immunostaining displayed in (**A**). (**C**) Representative images of immunostaining for ZO-1 and CD31 in cortex. Scale bar: 20 µm. (**D**) The percentage of expressed area of ZO-1 immunostaining displayed in (**C**). Bars represent mean ± SEM. Statistics were performed with one-way ANOVA Bonferroni's post hoc test for multiple comparisons. BBB, blood-brain barrier; SCFAs, short-chain fatty acids.

3. The vagus nerve is indeed an interesting player in the gut microbiota effects on the brain barriers as e.g. shown for the BBB by Yang et al.. In this study, they showed that non-invasive vagus nerve stimulation reduces BBB disruption in a rat model of ischemic stroke (Yang et al., 2018). In our study, we found that vagotomy results in loss of blood-CSF barrier integrity in wildtype mice as shown in **Figure 5A-F**. However, administration of SCFAs can still improve the blood-CSF barrier integrity in vagotomized mice as shown in **Figure 5H-M**. Additionally, our *in vitro* primary CPE cell culture experiment revealed that SCFAs can directly act on the blood-CSF barrier and improve its barrier tightness as shown in **Figure 4A-E**. Our results shown in **Figure 4** and **5** (showed in comment (3)) suggest that gut microbiota may act on blood-CSF barrier in a vagus nerve-dependent manner, while some SCFAs effects might work independent from the vagus nerve.

4. Related to the comment that the AD data seem to be preliminary, we want to stress that the purpose of including AD data in this study was to use it as an endpoint to highlight the benefits of gut microbiota and/or SCFAs in maintaining blood-CSF barrier integrity and thus affecting AD progression. We don't agree that this should be included in a separate study. However, to address this comment, we included more experiments in the revised manuscript to assess the effect of SCFA on activation of glial cells in wildtype mice and on synaptic pruning in *App*$^{NL\text{-}G\text{-}F}$ mice. Please check

our response to the comments 8.3-8.5 below.

5. The study started with a bulk RNAseq analysis of the choroid plexus from mice with healthy microbiota vs. mice treated with antibiotics. The analysis yielded intriguing results pointing towards reduced expression of barrier related genes. However, none of the results are validated by an alternative approach such as qPCR. Similarly, analysis on SCFA pathways (Fig 3) was based on RNAseq data without any further validation.

We agree that qPCR validation is important for RNA-seq data interpretation. We have now included qPCR results on the top DEGs related to barrier function and propionate metabolism as shown in **Fig EV2** and **Appendix Figure S2**. The text has also been adjusted accordingly in the revised manuscript.

Page 6, line 143-152

> *"Lastly, we investigated the expression of RNA-seq-detected DEGs in choroid plexus of SPF, AB, ABR, germ-free (GF) and recolonized GF (GFR) via qPCR, focusing on the genes associated with barrier function. Consistent with the RNA-seq results, Cbln1 gene expression was significantly downregulated in AB mice compared to SPF mice, while all other tested DEGs showed only a trend towards downregulation (**Fig EV2**). In addition, this downregulation appeared to be suppressed to some extent upon gut microbiota reconstitution (ABR; **Fig EV2**). In GF mice, Fat2, Tmem252, Slc13a3, Slc6a13 and Aqp4 showed trends of downregulation similar to AB mice, but none of these genes showed upregulation upon gut microbiota recolonization (GFR; **Fig EV2**)."*

Page 7, line 201-204

> *"The RNA-seq data were validated using qPCR and focusing on several top DEGs including Acss1, Acss3, Acat1, Acaca and Suclg2. Consistent with the RNA-seq data, these genes showed upregulation in AB mice compared with SPF mice. However, this was only signification in case of Acat1 (**Appendix Figure S2**)."*

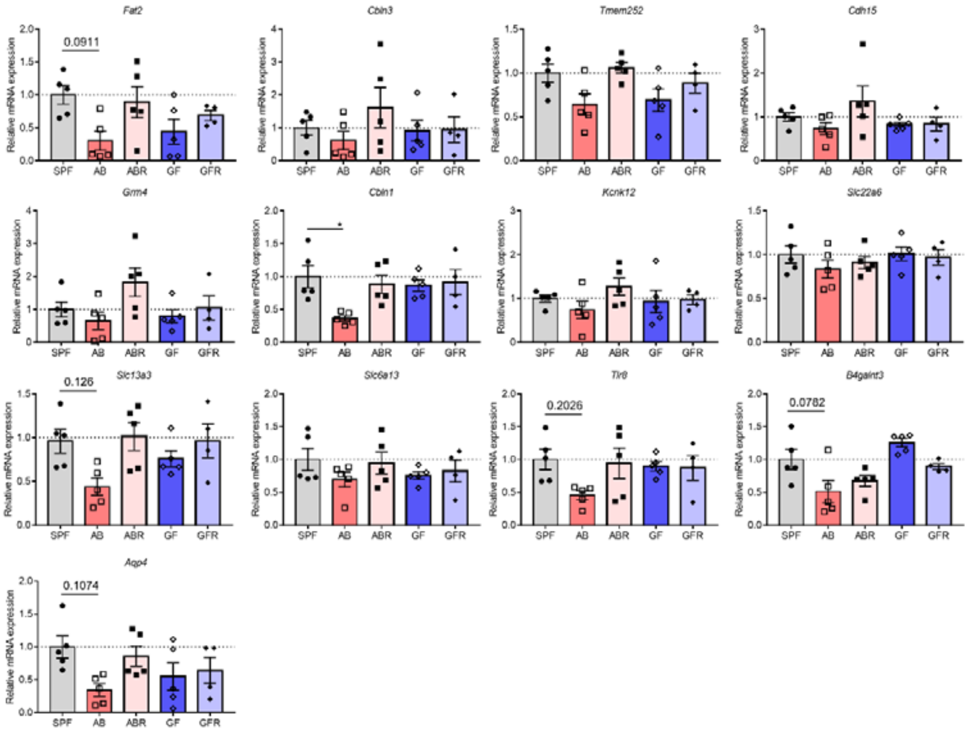

Figure EV2. qPCR analysis for barrier function-associated genes on choroid plexus of SPF, AB, ABR, GF and GFR mice. Bars represent mean ± SEM. Statistics were performed with one-way ANOVA Bonferroni's post hoc test for multiple comparisons. *$p < 0.05$. AB, antibiotics-treated; ABR, recolonized AB; GF, germ-free; GFR, recolonized GF; SPF, specific pathogen-free.

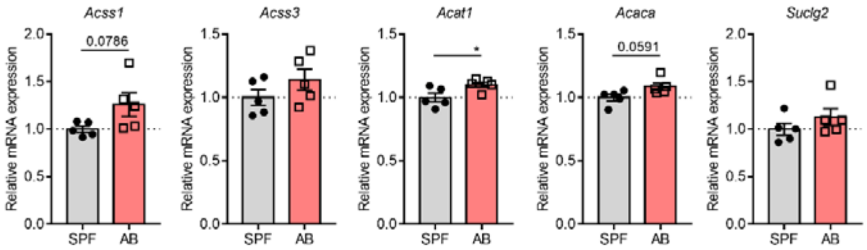

Appendix Figure S2. qPCR analysis for genes linked to the KEGG pathway mmu00640 (Propanoate metabolism) on choroid plexus of SPF and AB mice. Bars represent mean ± SEM. Statistics were performed with two-tailed Student's t-test. *$p < 0.05$. AB, antibiotics-treated; SPF, specific pathogen-free.

6.1 Most conclusions are drawn from imaging data collected from immunohistochemistry, but there are two major concerns: The images could benefit from more quantification. With single panels shown for most experimental conditions in the main figures, it is difficult to reliably evaluate the phenotypes. For example, the minimal and maximal length of continuous ZO-1 or Occludin signal, and the overall distribution of these values, can be used to demonstrate disruption of TJ. The thickness or total area can be other helpful parameters.

It is indeed important to assess the barrier integrity by quantifying different parameters of TJ immunostaining. In our revised manuscript, we have quantified 3 parameters (i.e.

continuous length, maximal length and expressed area) of TJ immunostaining in **Figure 2A-D**, **4A-E**, **5A-D**, **5H-K**, **6B-E**, **EV3A-D** and **Appendix Figure S7A-D** to assess the barrier integrity. Please check our response to comment 2.

6.2 It is not specified in the method section the number of tissue sections analyzed per animal, and their anatomical locations. Maybe adding some data from the supplemental figures into the main figures would also help. Choroid plexus anatomy can also vary between anterior to posterior locations even in the same ventricle. In AD genetic models it is known that Ab deposition and pathology vary drastically even between brain hemispheres. These details are essential to incorporate into evaluating and interpreting the data.
We added more details on the tissue sections and regions of interest in the Methods section of the revised manuscript and/or in a schematic graph (**Appendix Figure S13**), as shown below. In addition, we selected 5 supplementary figures to be included in the article as Expanded View figures (i.e. **Figure EV1-5** in revised manuscript) in order to improve their accessibility, visibility and utility according to the guidelines of *The EMBO Journal*.

Page 20, line 686-689

> "*These brain samples were cut from the sagittal superior sinus towards the border of the cerebral hemisphere. Sections were collected serially for TJ staining when the choroid plexus was initially present and for other staining when the entire hippocampus was initially present (**Appendix Figure S13**). One section per mouse was analyzed.*"

Page 20, line 711-712

> "*For TJ stainings, choroid plexus located in the lateral ventricles was evaluated in all experiments.*"

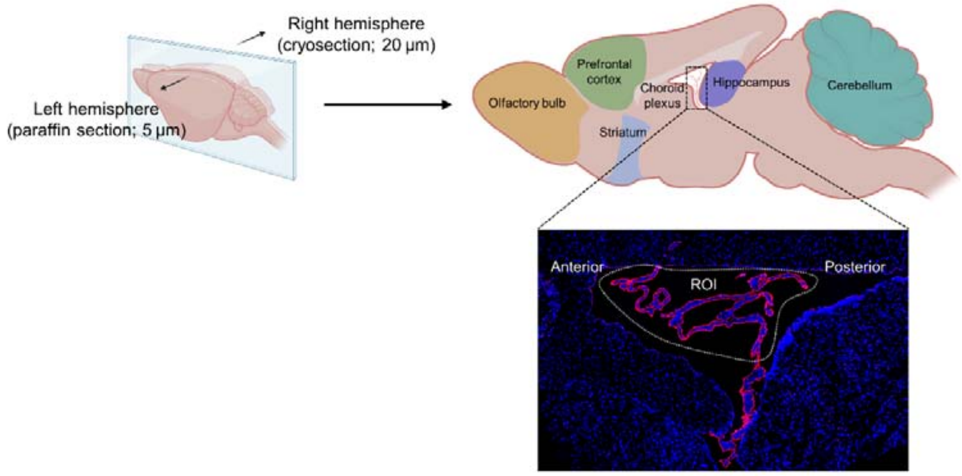

Appendix Figure S13. Sagittal section of adult mice brain. ROI: region of interest.

7.1 It is worrying how the impact of propionate and butyrate can vary from model to model. In this study, it appeared the propionate had stronger rescue effect in the in vitro LPS

model and in vivo microbiota removal model. However, butyrate had stronger impact on Ab pathology while propionate showed stronger induction of microglia phenotype. There is no explanation provided regarding the potential mechanisms.

We agree with the comment of the referee that propionate and butyrate have variable effects on the barrier function and AD pathology (Aβ plaque and microglial activation). Indeed, the propionate shows stronger rescue effects on barrier integrity disruption. This may be due to differences in their stability in the blood, the expression of corresponding receptors on the choroid plexus epithelial (CPE) cells or the uptake efficiency by CPE cells. In addition, the weaker rescue effect of both tested SCFAs on the barrier integrity in the *App*$^{NL\text{-}G\text{-}F}$ compared to WT mice may be due to the presence of AD pathology (e.g. Aβ plaques) as this will be a continuous trigger inducing barrier integrity disruption. While the slight improvement of the barrier integrity in the AD mice may play a role in the reduction of the AD pathology, we cannot exclude the possibility that SCFAs may affect microglial phenotype and Aβ pathology through additional mechanisms. In this regard, both propionate and butyrate were shown to induce microglial activation resulting in reduced Aβ accumulation by increasing Aβ phagocytosis, clearance, and degradation (Solito & Sastre, 2012). Importantly, we couldn't show the presence of the administered SCFAs in the brain, which is needed to exert direct effects on the microglia, while we did show that SCFA *in vitro* strengthen the blood-CSF barrier TJs directly. However, SCFAs may also indirectly affect microglial functions by mediating inflammation, as butyrate is a strong anti-inflammatory mediator, while other SCFAs such as propionate enhance the inflammatory response (Huuskonen et al., 2004). The inflammatory effects of SCFAs may further impact microglial activation and consequently microglial Aβ phagocytosis. In this study, we found that propionate induces higher levels of microglial activation than butyrate, and this state of hyperactivation may reduce their Aβ phagocytic capacity, as we demonstrated in our previous study (Xie et al., 2021). In addition to the inflammatory effects, SCFAs can also regulate microglia maturation and function, possibly through upregulation of the ApoE-TREM2 pathway and increasing histone acetylation (Bauer et al., 2022, Colombo et al., 2021, Erny et al., 2021, Erny et al., 2015); thereby affecting the ability of microglia in the internalization and degradation of Aβ (Lee & Landreth, 2010). The differences in propionate and butyrate in affecting these pathways may also contribute to their differential microglial Aβ phagocytosis. The above interpretations have been incorporated into the revised manuscript.

Page 12-13, line 386-393

> "*In our study, treatment with the SCFAs propionate and butyrate, in vitro and in vivo, prevented both the disorganization of TJs at the blood-CSF barrier and the increase of the barriers permeability induced by an inflammatory trigger. In general, butyrate treatment showed a weaker ability to improve the localization of TJs relative to propionate, which may, amongst others, be due to differences in their stability in the blood, the expression of corresponding receptors on the CPE cells or the uptake efficiency by CPE cells. However, which SCFA receptors are expressed on CPE cells is unclear and requires further investigation.*"

Page 14, line 443-472

"*In our study, we observed that treatment with SCFAs propionate and butyrate, improved the integrity of the BBB and blood-CSF barrier and decreased their permeability in App^{NL-G-F} mice. However, the rescue effect of both tested SCFAs on the barrier integrity is weaker in the App^{NL-G-F} mice compared to WT mice, which may be due to the presence of AD pathology (e.g. Aβ plaques) as this will be a continuous trigger inducing barrier integrity disruption. This slight improvement of the barrier integrity in the App^{NL-G-F} mice upon SCFA treatment was however associated with a significant reduction in Aβ accumulation in the brain, which is in concordance with previous reports showing that administration of sodium butyrate can decrease brain Aβ level and improve cognitive memory performance in a transgenic mouse model of AD (Fernando et al., 2020). Nevertheless, we cannot exclude the possibility that SCFAs may affect microglial phenotype and Aβ pathology through other mechanisms than brain barrier tightening. In this regard, both propionate and butyrate were shown to induce microglial activation resulting in reduced Aβ accumulation by increasing Aβ phagocytosis, clearance, and degradation (Solito & Sastre, 2012). Importantly, we couldn't show the presence of the administered SCFAs in the brain, which is needed to exert direct effects on the microglia, while we did show that SCFA in vitro strengthen the blood-CSF barrier TJs directly. However, SCFAs may also indirectly affect microglial functions by mediating inflammation, as butyrate is a strong anti-inflammatory mediator, while other SCFAs, such as propionate enhance the inflammatory responses (Huuskonen et al., 2004). The inflammatory effects of SCFAs may further impact microglial activation and then microglial Aβ phagocytosis. In this study, we found that SCFA treatment increased microglial Aβ internalization and the number of microglia adjacent to large Aβ plaques App^{NL-G-F} mice. Notably, propionate induces higher levels of microglial activation than butyrate, and this state of hyperactivation may reduce their Aβ phagocytic capacity, as we demonstrated in our previous study (Xie et al., 2021). In addition to the inflammatory effects, SCFAs can also regulate microglia maturation and function, possibly through upregulation of the ApoE-TREM2 pathway and increasing histone acetylation (Bauer et al., 2022, Colombo et al., 2021, Erny et al., 2021, Erny et al., 2015); thereby affecting the ability of microglia in the internalization and degradation of Aβ (Lee & Landreth, 2010).*"

7.2 What are the mechanisms of action of these fatty acids and how are they anticipated to mediate their effects on target tissues? Do they interact with specific receptors on target cells that could be tracked in the present study? Some dose-dependent studies may help shed light on the effects.

Based on literature, SCFAs can act on BBB function through direct interaction with receptors (e.g. free fatty acid receptors (FFARs)) located on target cells and/or indirectly via stimulation of the vagus nerve (Dalile et al., 2019). In our study, both *in vitro* and *in vivo* experiments demonstrate that SCFAs can directly impact the blood-CSF barrier integrity

(**Figure 4** and **5**). However, no expression of *Ffar* gene was detected in our (unpublished) choroid plexus tissue RNA-seq data (in agreement with publicly available data in Genevestigator MM-00583), suggesting that the direct action of SCFAs on the blood-CSF barrier via FFARs may not be the dominant mechanism. In addition, in the revised manuscript, we examined the effects of 0.1, 1 and 10 μM propionate and butyrate on LPS-treated primary CPE cell cultures. Both LPS-induced deficits in TEER and paracellular tracer permeability (**Appendix Figure S3B**) were significantly attenuated by treatment with propionate (all doses tested) and 1 μM butyrate. There were no obvious dose-dependent effects and the 1 μM of the SCFAs appeared to be the most effective. The corresponding text in figure, methods, results, and discussion have been adjusted appropriately.

<u>Page 8, line 221-228</u>

> "*Treatment of primary CPE cells for 6 h with propionate (0.1, 1 and 10 μM) or butyrate (1 μM) significantly attenuated the LPS-induced loss of barrier integrity (**Fig 4B, C** and **Appendix Figure S3B**). A dose-dependent effect was observed in propionate but not butyrate upon the response of CPE cells to LPS stimulation and 1 μM appeared to show the best barrier recovery effect in the case of butyrate (**Appendix Figure S3B**). It has been proposed that the physiological circulating concentrations of propionate and butyrate are approximately 1 μM at rest (Hoyles et al., 2018).*"

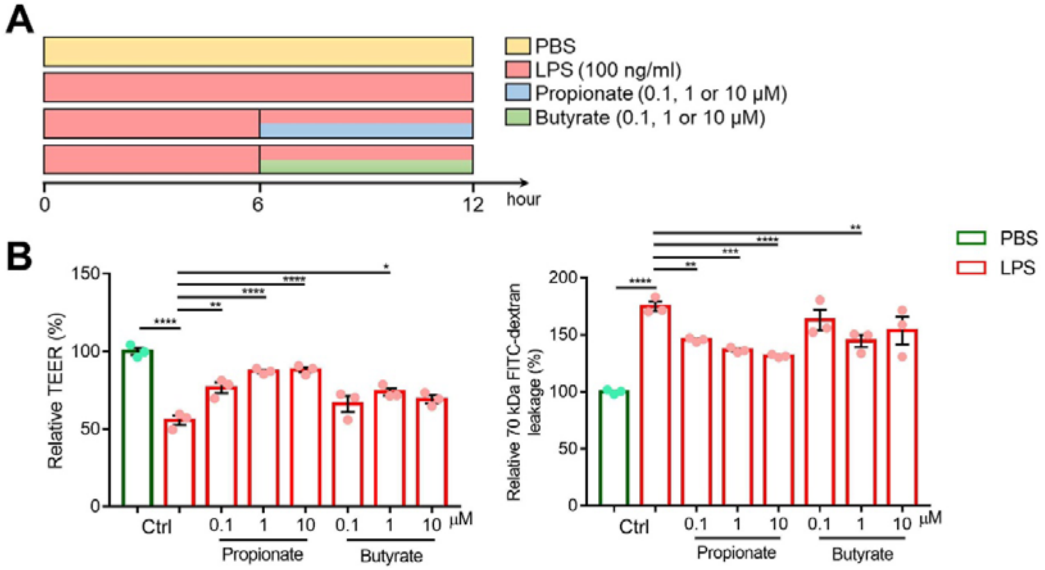

Appendix Figure S3. Restoring effects of SCFAs against LPS-induced blood-CSF barrier disruption in primary CPE cells. (**A**) Schematic representation of the experimental conditions. (**B**) TEER (left) and assessment of the 70 kDa FITC-dextran paracellular permeability (right) of primary CPE cells treated as showed in (**A**). Bars represent mean ± SEM. Statistics were performed with one-way ANOVA Bonferroni's post hoc test for multiple comparisons. *$p < 0.05$, **$p < 0.01$, ***$p < 0.001$, ****$p < 0.0001$. CPE, choroid plexus epithelial; CSF, cerebrospinal fluid; LPS, lipopolysaccharides; SCFAs, short-chain fatty acids; TEER, trans-epithelial electrical resistance.

7.3 In addition, how are they transported to the blood-CSF barrier from gut? Are they

present in the CSF? Are the CPE cells cells exposed to SCFA through their basal or apical surface?

This is a very interesting comment as it is unclear whether SCFAs reach the brain and whether they can cross the brain barriers and subsequently act on both the basal and apical surface of the cells that form the brain barrier. To address this, we first performed *in vitro* primary CPE cell culture experiments in a transwell system, as shown in **Figure EV4A**,**B**. In this experiment, we added 10 µM sodium propionate or sodium butyrate at the basal side of CPE cells and collected the medium from the apical side after 6 hours to determine the brain barrier crossing capacity of the SCFAs. The results revealed that approximately 40% of the sodium butyrate can cross the blood-CSF barrier from basal to apical side (**Figure EV4B**). Unfortunately, using this setup, the sensitivity of the sodium propionate measurements was too low so we have no information on the propionate transport. Our samples were analyzed onto an ion-pairing operating liquid-chromatography mass spectrometer (LC-MS, OrbiTRAP). Based on the analysis of the standard, we noticed that the compound has an intrinsic low ionisation potential. This implies that the ability to detect the compound by the current methodology is less sensitive and requires higher amounts of the propionic acid in the target samples compared to other small polar molecules. As a consequence, the limit of detection (LOD) of propionic acid (determined at 5-7 µM) was too low to measure the levels even in the basal compartment.

Next, we also performed *in vivo* experiments to study whether SCFAs can reach the brain. Thereto, mice were treated with sodium propionate or sodium butyrate, followed by analysis of plasma and CSF. To ensure the SCFAs detected in the plasma and CSF came from the gut lumen, WT mice were orally gavaged with 13C-labeled sodium propionate or sodium butyrate; this labeled SCFAs were selected to be able to discriminate between endogenous and exogenous SCFA. In plasma, we could detect 13C sodium propionate and 13C sodium butyrate after 1 and/or 2 h of SCFAs treatment (**Fig EV4**). In contrast, none of them could be detected in CSF. However, we can't rule out that this is due to limited CSF sample volume (~7 µl per mouse) and detection limit. As explained above, our methodology to detect propionic acid requires higher amounts of the SCFAs in the target samples (plasma and CSF) compared to other small polar molecules. Consequently, we don't rule out the possibility that SCFAs can cross the blood-CSF barrier and act on both basal and apical sides as we did see transport *in vitro*. These results and explanations have been incorporated into the revised manuscript.

<u>Page 8, line 237-240</u>

> "*Additionally, we found that approximately 40% sodium butyrate crossed the in vitro blood-CSF barrier from basal to apical side (**Fig EV4A, B**). Unfortunately, sodium propionate was undetectable, even in the basal compartment, due to the low limit of detection (LOD) that was 5-7 µM.*"

<u>Page 9, line 267-274</u>

> "*We next determined whether the gut-derived SCFAs can be transported to the brain and cross the blood-CSF barrier. Thereto, WT mice were orally gavaged with 13C-labeled sodium propionate or sodium butyrate (**Fig EV4C**). In plasma,*

*we could detect 13C-labeled sodium propionate and sodium butyrate after 1 and/or 2 h of treatment (**Fig EV4D**). In CSF, neither 13C-labeled sodium propionate or sodium butyrate was detectable, which might either be due to absence of transport in vivo or due to limited CSF volume (~7 µl per mouse) and LOD (propionate: 5-7 µM; butyrate: 0.05 µM).”*

<u>Page 13, line 425-431</u>

“*In our study, we found that SCFAs can enter the bloodstream upon oral administration. Although we were unable to determine based on our current methods whether SCFAs could also cross the blood-CSF barrier and enter the CSF, our in vitro model showed that sodium butyrate can cross the tightly connected CPE cells from basal to apical side. Thus, we speculate that gut-derived SCFAs can reach the brain and cross the blood-CSF barrier. However, further validation by more sensitive methods is required to be sure whether or not SCFAs enter the CSF.”*

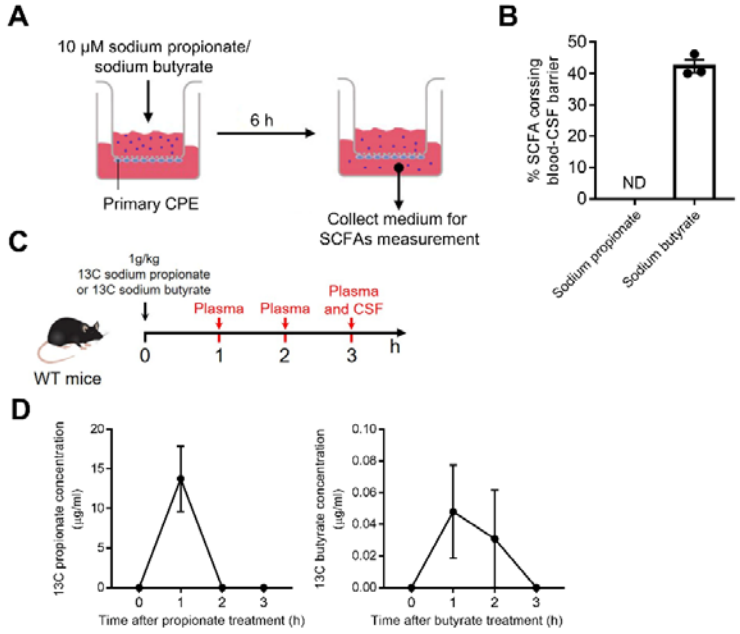

Figure EV4. The distribution of SCFAs in *in vitro* and *in vivo*. (**A**) Schematic representation of the *in vitro* experimental conditions. (**B**) The percentage of SCFAs crossing the blood-CSF barrier *in vitro*. ND, not detected. (**C**) Schematic representation of the *in vivo* experimental conditions. (**D**) The concentrations of 13C sodium propionate (left) and 13C sodium butyrate (right) in plasma. Data information: Bars represent mean ± SEM. SCFAs, short-chain fatty acids.

8.1 The investigation in the AD model seems incomplete. In addition to absence of information on number of sections and anatomical locations as mentioned before, several additional questions emerge: Ab pathology was evaluated across the entire brain but microglial phenotypes were analyzed in the hippocampus only. Is hippocampal pathology particularly decreased compared to the rest of the brain? Is the same microglial phenotype observed in other brain regions?

We only analyzed the microglial phenotype in the hippocampus, as this is the region close to the ventricle and plays a major role in memory. As added in revised manuscript, we also investigated the microglial proliferation in olfactory bulb, cortex, striatum and cerebellum (**Figure EV5**).

Page 10, line 313-318

> *"A significantly increased number of microglia was observed in the olfactory bulb of propionate and butyrate-treated App$^{NL\text{-}G\text{-}F}$ mice, while only propionate treatment induced a pronounced increase in the number of microglia in the hippocampus and cerebellum. In cortex and striatum, the number of microglia did not show a difference after SCFAs treatment (**Fig 7A, B** and **Fig EV5A, B**)."*

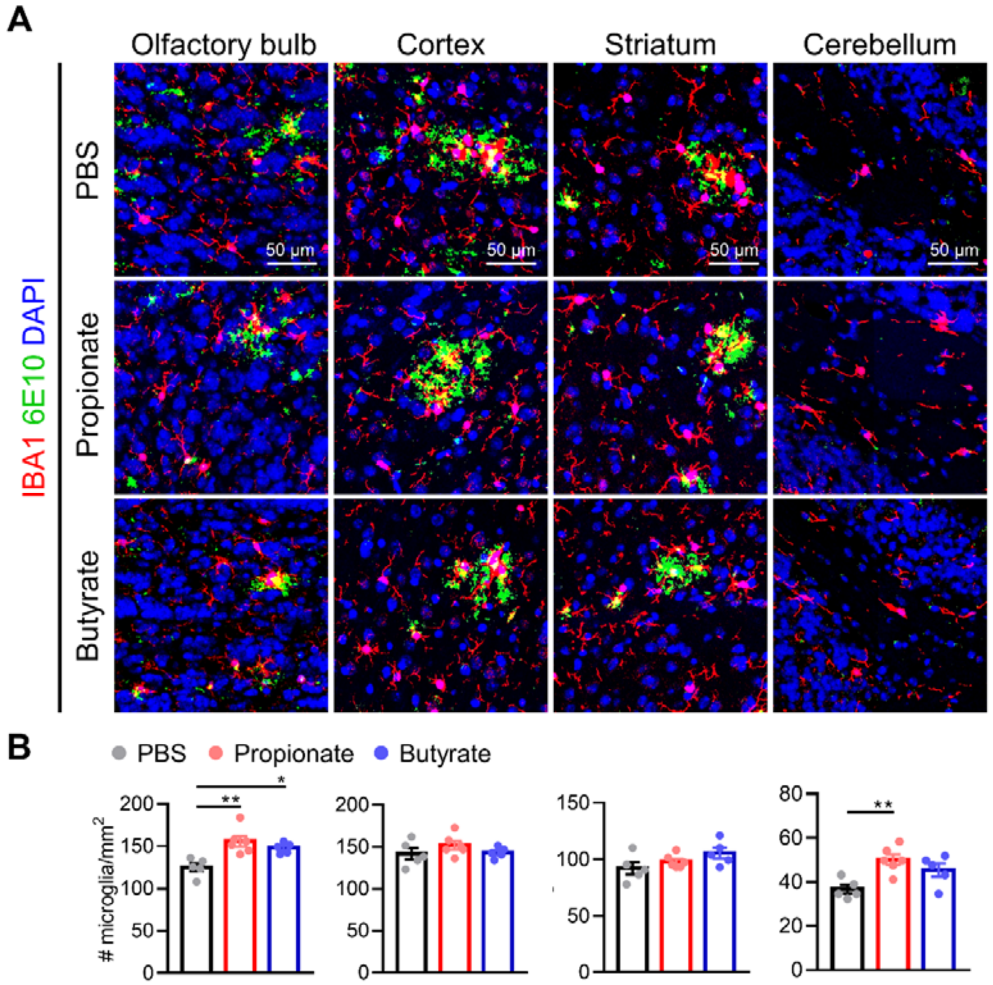

Figure EV5. The effects of SCFAs on microglial proliferation in *App*$^{NL\text{-}G\text{-}F}$ mice. (**A**) Representative images of immunostainings for IBA1 and 6E10 in olfactory bulb, cortex, striatum and cerebellum. Scale bar: 50 μm. (**B**) The number of IBA1^{+} microglia in different regions of the brain. Bars represent mean ± SEM. Statistics were performed with one-way ANOVA Bonferroni's post hoc test for multiple comparisons. **p* < 0.05, ***p* < 0.01. SCFAs, short-chain fatty acids.

8.2 It is surprising to see such a robust reduction of Ab deposit with only 3 days of treatment. What is the hypothesis regarding such fast reduction of plaques? How are the

plaques quantified? What is the area cutoff to define a plaque? Are there more microglia associated with plaques? Are there increased Ab signal within microglia, suggesting increased phagocytosis? What is the level of soluble Ab in CSF and ISF, or overall brain lysate?

The Aβ plaques were quantified by Image J in Analyze Particles with a minimal plaque area of 100 μm^2 cutoff. Indeed, propionate and butyrate treatment increased the area of Aβ internalization by microglia and the number of Aβ-associated microglia. These results suggest that propionate and butyrate may reduce the Aβ through promoting microglial Aβ phagocytosis (**Figure 7E** and **F**). The text has been adapted accordingly in the revised manuscript.

Page 10, line 322-326

> "*Additionally, we also observed a markedly increase in microglial internalized Aβ in both propionate and butyrate-treated App^{NL-G-F} mice, but only butyrate treatment showed a significant increase in the number of microglia adjacent to the large plaques (> 600 μm^2) in App^{NL-G-F} mice (**Fig 7E, F**).*"

Page 14, line 464-468

> *"In this study, we found that SCFA treatment increased microglial Aβ internalization and the number of microglia adjacent to large Aβ plaques in App^{NL-G-F} mice. Notably, propionate induces higher levels of microglial activation than butyrate, and this state of hyperactivation may reduce their Aβ phagocytic capacity, as we demonstrated in our previous study (Xie et al., 2021)."*

We didn't determine the soluble Aβ in CSF, ISF or brain lysate as this would require new samples (CSF amounts only allow very limited analyses) and we don't see much added value of these measurements for the current story.

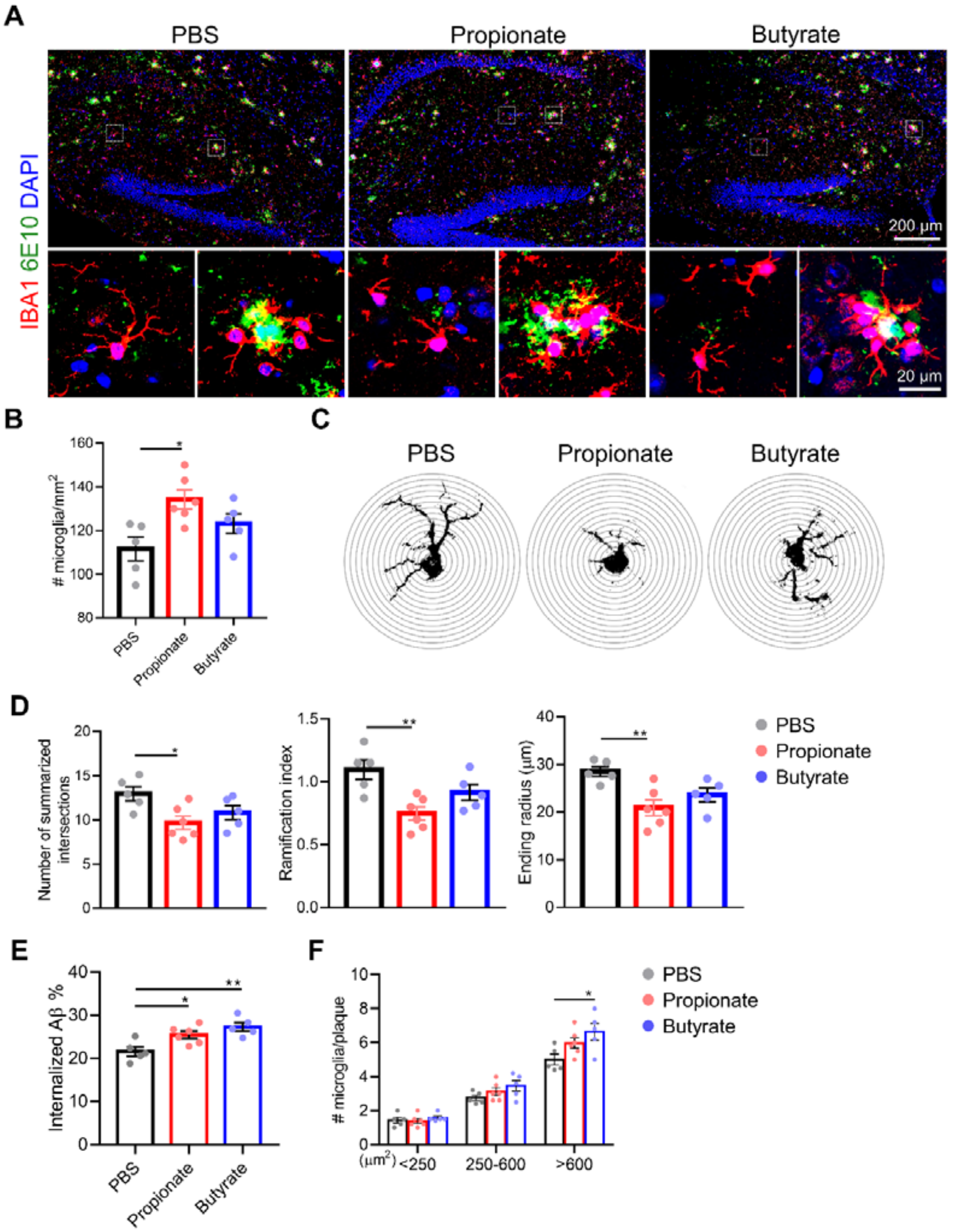

Figure 7. Effects of SCFAs on microglial proliferation and activation in *App*$^{NL\text{-}G\text{-}F}$ mice. (**A**) Representative images of immunostainings for IBA1 and 6E10 in hippocampus of sodium propionate or sodium butyrate-treated *App*$^{NL\text{-}G\text{-}F}$ mice. Scale bar: 200 μm. (**B**) The number of IBA1^{+} microglia in hippocampus. (**C**) Representative image for Sholl analysis of non-Aβ associated microglia in the hippocampus from the IBA1-stained image in (**A**). The interval of the concentric circles is 2 μm. (**D**) Quantification of summarized intersects, ramification index and ending radius by Sholl analysis from immunostained IBA1 signals. (**E**) Quantification of the percentage of overlay area of microglia and Aβ plaque. (**F**) Plaques were divided into small (< 250 μm^2), medium (250-600 μm^2), and large (> 600 μm^2), and the number of microglia per plaque was quantified. Bars represent mean ± SEM. Statistics were

performed with one-way ANOVA Bonferroni's post hoc test for multiple comparisons. *$p < 0.05$, **$p < 0.01$. SCFAs, short-chain fatty acids.

8.3 Reactive microglia may cause additional damage. What is the synapse density, typically measured by synaptophysin-PSD95 co-localization, for example after 3 days? Are astrocytes activated at the same time?

Based on the same concerns as the referee about microglial dysfunction, we have conducted synaptic stainings. No obvious synaptic loss or excessive synaptic pruning by microglia after propionate and butyrate treatment in $App^{NL\text{-}G\text{-}F}$ mice were observed (**Appendix Figure S11**). Also, there were no significant changes in astrocyte proliferation and activation (**Appendix Figure S9** and **S10**). The corresponding text in figure, methods, results, and discussion have been altered appropriately.

Page 11, line 336-338

> "*First, we examined the effects of propionate and butyrate on proliferation and activation of astrocyte in WT and* $App^{NL\text{-}G\text{-}F}$ *mice (**Appendix Figure S9A, B** and **Appendix Figure S10A, B**). GFAP immunostaining showed no significant changes in astrocyte morphology nor number in WT and* $App^{NL\text{-}G\text{-}F}$ *mice after propionate and butyrate treatments (**Appendix Figure S9A, B** and **Appendix Figure S10A, B**).*"

Page 11, line 341-348

> "*Next, we analyzed the effects of propionate and butyrate on synaptic properties in* $App^{NL\text{-}G\text{-}F}$ *mice (**Appendix Figure S11A-D**). High-resolution images of co-immunostaining of presynaptic protein synaptophysin (SYP) and postsynaptic density protein 95 (PSD-95) showed similar number of double-positive synaptic puncta in the area of the cortex and hippocampus of SCFA-treated* $App^{NL\text{-}G\text{-}F}$ *mice compared to* $App^{NL\text{-}G\text{-}F}$ *controls (**Appendix Figure S11A, B**). Moreover, co-immunostaining of IBA1 and PSD-95 did not show excessive synaptic pruning by microglia and even a downregulation trend observed (**Appendix Figure S11C, D**).*"

Page 15, line 491-497

> "*In addition, previous studies have shown that microglial activation may lead to the conversion of resting astrocytes to reactive astrocytes and neuronal dysfunction such as excessive synaptic pruning (Azevedo et al., 2013, Leng & Edison, 2020, Litvinchuk et al., 2018), but we did not observe changes in astrocyte proliferation and activation, nor synaptic loss in SCFA-treated* $App^{NL\text{-}G\text{-}F}$ *mice. These results indicate that SCFA-induced increased phagocytic activity and proliferation of the microglia has no effect on astrocyte activation and synaptic pruning.*"

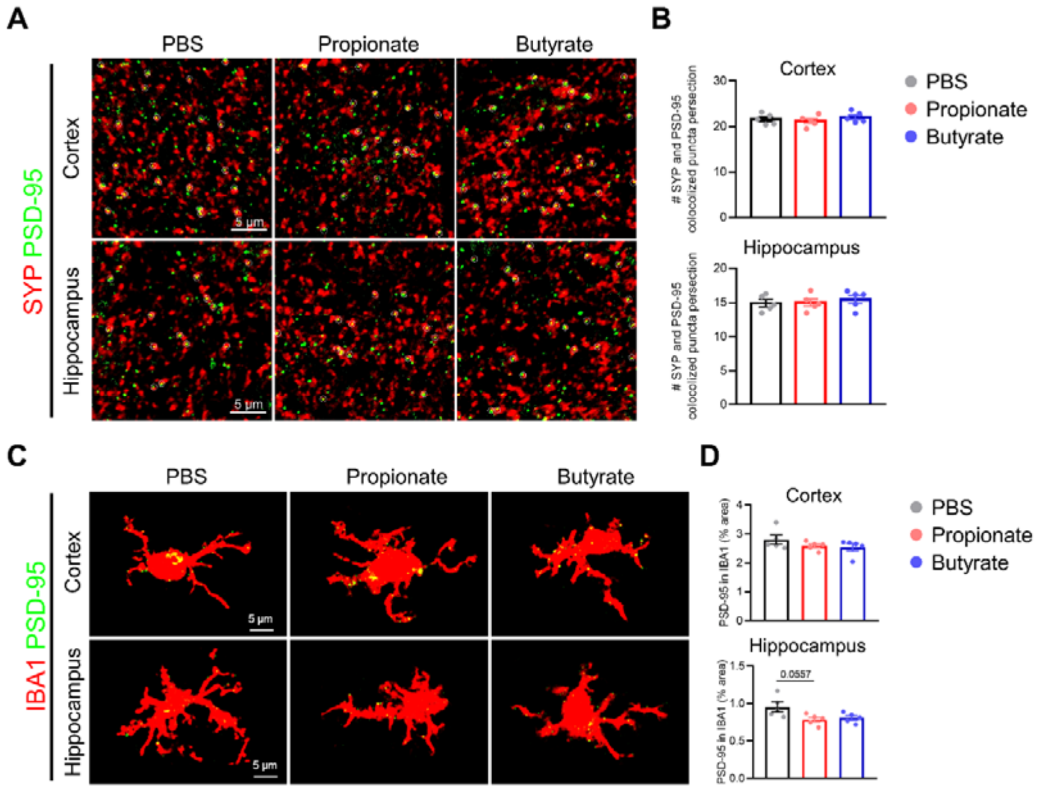

Appendix Figure S11. The effects of SCFAs on synapse elimination by microglia in *App*$^{NL\text{-}G\text{-}F}$ mice. (**A**) Representative high magnification confocal images of SYP and PSD-95 coimmunostaining in cortex and hippocampus of *App*$^{NL\text{-}G\text{-}F}$ mice. Scale bars, 5 μm. (**B**) Quantification of the number of colocalized puncta of SYP and PSD-95. (**C**) Representative images of IBA1 and PSD-95 coimmunostaining in cortex and hippocampus of *App*$^{NL\text{-}G\text{-}F}$ mice. (**D**) Quantification of the internalized PSD-95 in microglia. Bars represent mean ± SEM. Statistics were performed with one-way ANOVA Bonferroni's post hoc test for multiple comparisons. SCFAs, short-chain fatty acids; PSD-95, postsynaptic density protein 95; SYP, synaptophysin.

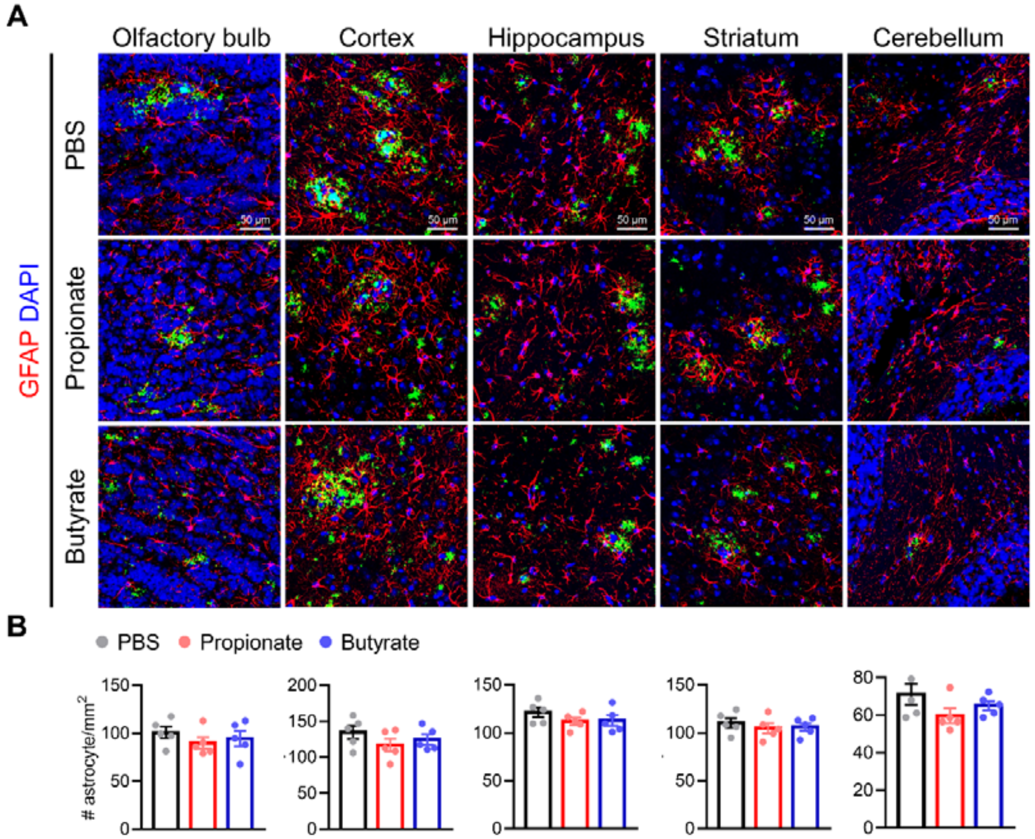

Appendix Figure S9. The effects of SCFAs on astrocyte proliferation and activation in *App*$^{NL\text{-}G\text{-}F}$ mice. (**A**) Representative images of immunostainings for GFAP and 6E10 in hippocampus of sodium propionate or sodium butyrate-treated *App*$^{NL\text{-}G\text{-}F}$ mice. Scale bar: 50 µm. (**B**) The number of GFAP^{+} astrocytes in different regions. Bars represent mean ± SEM. Statistics were performed with one-way ANOVA Bonferroni's post hoc test for multiple comparisons. SCFAs, short-chain fatty acids.

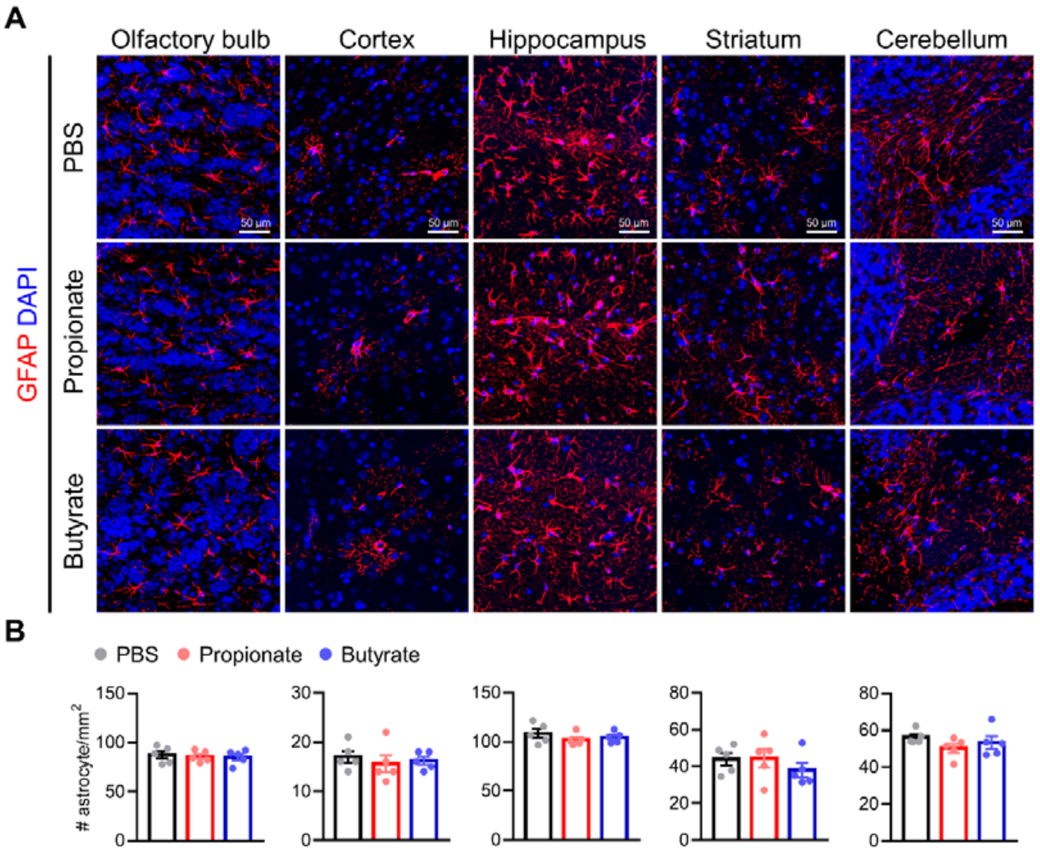

Appendix Figure S10. The effects of SCFAs on astrocyte proliferation and activation in WT mice. (**A**) Representative images of immunostainings for GFAP in different of brain of sodium propionate or sodium butyrate-treated WT mice. Scale bar: 50 μm. (**B**) The number of $GFAP^{+}$ astrocytes in different regions. Bars represent mean ± SEM. Statistics were performed with one-way ANOVA Bonferroni's post hoc test for multiple comparisons. SCFAs, short-chain fatty acids.

8.4 Microglia are already at a very different state in AD mice *vs.* their wild-type controls. Are these microglial changes by SCFA only present in AD mice or also present in wild-type mice treated with these SCFAs? The current analysis is seems too preliminary to yield meaningful understanding of the changes in AD. As presented, it is unfortunately difficult to draw conclusions on propionate and butyrate functions in AD.

This is an interesting remark. To address this, we investigated the impact of SCFAs on microglial proliferation and activation in WT mice. These results and discussions have been included in the revised manuscript.

Page 10, line 326-328

> *"However, when administering the same doses of propionate and butyrate in WT mice, we didn't observe difference in microglial proliferation or activation (**Appendix Figure S8A-D**)."*

Page 15, line 481-489

> *"In our study, we observed that SCFA treatment increased the number of*

microglia and induced microglial activation in the hippocampus of App$^{NL\text{-}G\text{-}F}$ mice but not WT mice, indicating that under physiological condition, supplementation of SCFAs has no pronounced effect on microglial proliferation and activation. In contrast, under pathological condition, SCFA treatment was associated with increasing the number of microglia and inducing microglial activation which on its turn explain the observed reduction of Aβ burden in the brain. The microglia may benefit from the barrier-restoring effects of SCFAs, which limit the leakage of neuroinflammation-inducing peripheral factors into the brain, thereby preventing microglial hyperactivation and the maintenance of their functionality."

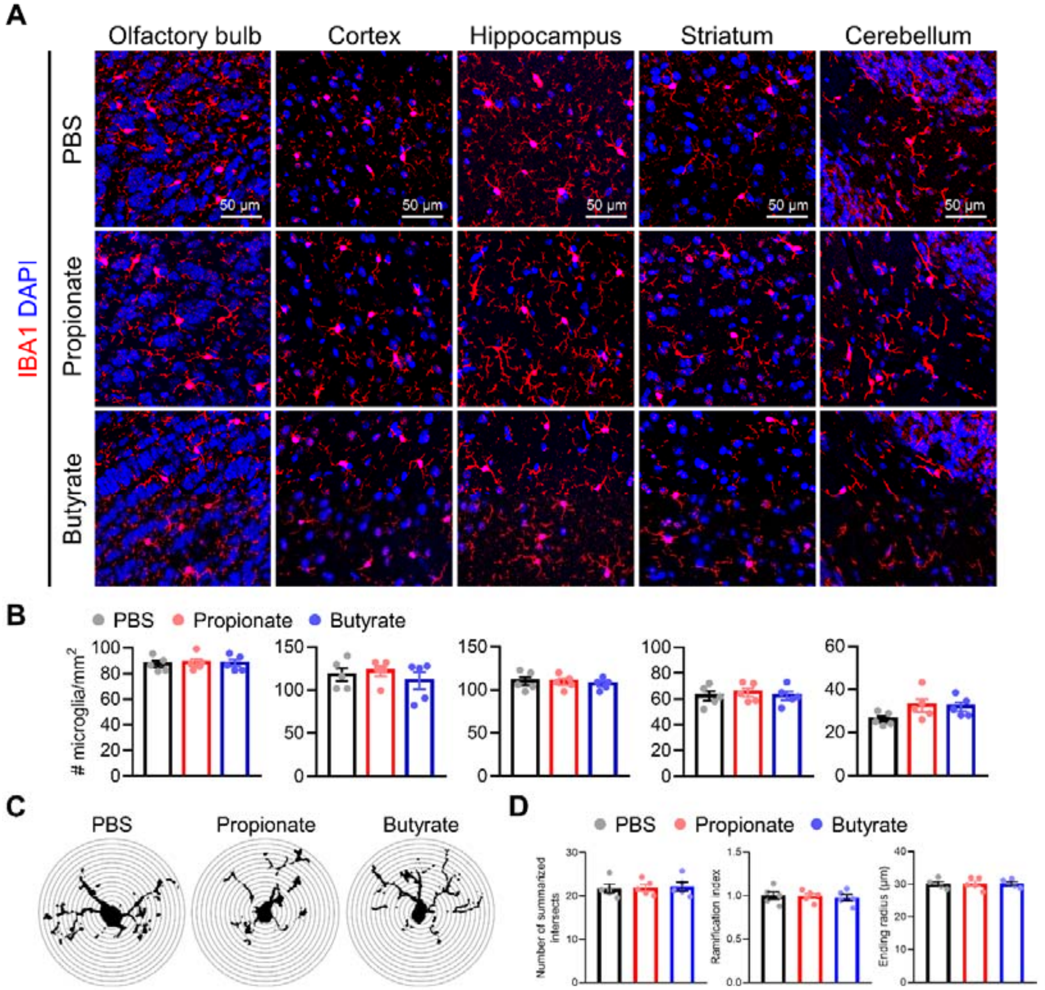

Appendix Figure S8. The effects of SCFAs on microglial proliferation and activation in WT mice. (**A**) Representative images of immunostainings for IBA1 in different regions of brain of sodium propionate or sodium butyrate-treated WT mice. Scale bar: 50 µm. (**B**) The number of IBA1^{+} microglia in different regions of brain. (**C**) Representative image for Sholl analysis of microglia in the hippocampus from the IBA1-stained image in **A**. The interval of the concentric circles is 2 µm. (**D**) Quantification of summarized intersects, ramification index and ending radius by Sholl analysis from immunostained IBA1 signals. Bars represent mean ± SEM. Statistics were performed with one-way ANOVA Bonferroni's post hoc test for multiple comparisons. SCFAs, short-chain fatty acids.

9. Overall, the statistics appear weak with a relatively small replicate numbers. Providing exact P values would also allow for better evaluation.

We have now increased the n numbers to a minimum of 5 in **Figure 4F-M**, **6** and **7** (showed in comment 3) in the revised manuscript. To avoid showing complicated statistics results in the figures, we only indicated the significant statistics results of the most important comparisons such as SPF *vs.* AB, SPF *vs.* GF, AB *vs.* ABR and GF *vs.* GFR in the figures. More details of numerical data and statistics results have been provided in **Source data** files.

Referee #3

Though increased BBB permeability in AD pathology has been well documented, blood-CSF barrier is not well studies. The current study is attempting to dissect the influence of gut microbiota on its barrier function. Here, Jie et al., show that gut microbiota deficiency enhances blood-CSF barrier permeability, associated with disorganised tight junction proteins (TJs). Recolonization with normal gut microbiota or SCFA rescues the defective blood-CSF barrier by improving TJ subcellular distribution. Notably, vagotomy also disrupts blood-CSF barrier which was improved by SCFA. Employing a *App*$^{NL\text{-}G\text{-}F}$ AD mice, an AD mouse model that display increased blood-CSF barrier permeability and decreased SCFA in feces, the authors found that SCFA administration improve TJs localization in the blood-CSF barrier. This treatment leads to Abeta burden reduction, accompanied with activated microglia. Overall, these findings are interesting, and the data fit with the main conclusion. Nevertheless, the observation does not represent a striking conceptual advancement that EMBO J. is seeking for.

Major concerns:

Especially, most of the data basically recapitulated previously published reports, lacking of innovative observation. For instance, SCFAs activate microglia and regulate Abeta clearance, and they mediates BBB or Blood/CSF barriers. These were well known in the literature.

We dispute the concern stating that our study lacks novelty. Prior to submission of our manuscript, there were no data available on the effect of the gut microbiota and SCFAs on blood-CSF barrier integrity, despite increasing evidence that this central nervous system (CNS) barrier has an important and central role in periphery-to-brain communication as it is the main source of CSF production and controlled transport of molecules into the brain. Unfortunately, it remains a barrier that does not receive the attention and focus in the research community that it deserves and urgently needs. An important reason causing the science to lag behind on this topic is the challenging technical aspects that it entails. Being able to investigate the tiny choroid plexus tissue requires very specialized expertise and is very time-consuming. Moreover, the small size of the tissue implicates that the number of analyses per choroid plexus (and thus per mouse) are very limited. As clearly indicated in our revised manuscript, a previous publication in *Sci Transl Med* showed the absence of a properly formed BBB in germ free mice, while this could be restored upon addition of SCFAs or recolonization with SCFA-producing bacteria (Braniste et al., 2014).

Of note, next to revealing the impact of the gut microbiome and SCFAs on blood-CSF barrier integrity and confirming the reported BBB data, an additional novel and striking finding of our study is that the gut microbiome is not only needed to **establish** tight CNS barriers, but it is also essential to **maintain** both CNS barriers.

We of course agree that there are already data available about the impact of the microbiome on Alzheimer's disease using mouse models (e.g. the very recent Science publication highlighted on Alzforum (https://www.alzforum.org/news/research-news/meddling-microbiome-worsens-tauopathy-and-neurodegeneration). However, in our study we used the most recently developed

transgenic mouse model of AD, namely the *App*$^{NL-G-F}$ mouse model, which overcomes intrinsic drawbacks of the most often used APP overexpressing mouse models (Saito et al., 2014). Currently, there are no studies investigating the effects of gut microbiome depletion/absence or SCFAs on AD pathology using the *App*$^{NL-G-F}$ mice, while this is essential to determine which findings might be a consequence of overexpression artifacts. There are currently several reports on the mechanism of action of SCFAs, as e.g. summarized in a recent review (Qian et al., 2022). This review shows why we have to disagree with the reviewer as there are still a lot of unanswered questions related to which SCFA mechanism(s) are important in e.g. AD. Consequently, our newly identified mechanism, namely tightening of the blood-CSF barrier, even in the absence of a functional vagus nerve, further adds to the complexity of the potential therapeutic effects of SCFAs.

Minor concerns:

It is unclear, how vagus nerve resectioned by vagotomy and SCFAs crosstalk in blood/CSF barrier regulation. In the experiment, it remains unclear whether single side or two sides' vagus nerves were resected. If both sides of vagus nerves are transected, the animal still survived? If only on side is resected, how could the authors conclude "SCFA can bypass the vagus nerve and affect Blood-CSF barrier permeability"

We performed a total abdominal vagotomy, so both the left and right vagal trunks were transected. We confirmed the success of vagotomy by anti-ChAT immunostaining on the dorsal motor nucleus of the vagus (DMV). As shown in **Appendix Figure S12**, vagotomy induced a significant reduction in the number of ChAT positive neurons at both sides of DMV when compared to the non-vagotomized (sham) DMV. We did not observe death of mice due to the vagotomy. The text in the revised manuscript has been adjusted accordingly, as shown below.

Page 16, line 524-531

> *"To perform celiac vagotomy, the mice were incised at the central abdomen to expose the front wall of the esophagus and then the left and right vagal trunks were subdiaphragmatically transected. The non-vagotomized (sham) group underwent an incision of the central abdomen without vagotomy. We confirmed the success of vagotomy by anti-ChAT immunostaining on the dorsal motor nucleus of the vagus (DMV). As shown in* ***Appendix Figure S12****, vagotomy induced a significant reduction in the number of ChAT positive neurons at both sides of DMV when compared to the sham DMV."*

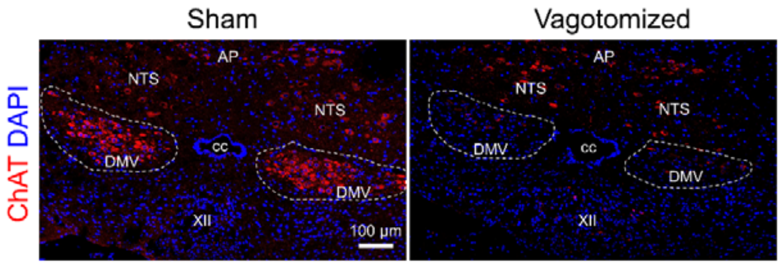

Appendix Figure S12. Validation of vagotomy by ChAT staining on DMV. Representative images of ChAT immunostaining on DMV of sham and vagotomized mice. Scale bar: 100 μm. AP, area postrema; CC, central canal; DMV, dorsal motor nucleus of the vagus; NTS, nucleus of the solitary tract XII, Hypoglossal nucleus.

Reference

Bauer KC, York EM, Cirstea MS, Radisavljevic N, Petersen C, Huus KE, Brown EM, Bozorgmehr T, Berdun R, Bernier LP, Lee AHY, Woodward SE, Krekhno Z, Han J, Hancock REW, Ayala V, MacVicar BA, Finlay BB (2022) Gut microbes shape microglia and cognitive function during malnutrition. *Glia* 70: 820-841

Braniste V, Al-Asmakh M, Kowal C, Anuar F, Abbaspour A, Toth M, Korecka A, Bakocevic N, Ng LG, Kundu P, Gulyas B, Halldin C, Hultenby K, Nilsson H, Hebert H, Volpe BT, Diamond B, Pettersson S (2014) The gut microbiota influences blood-brain barrier permeability in mice. *Sci Transl Med* 6: 263ra158

Colombo AV, Sadler RK, Llovera G, Singh V, Roth S, Heindl S, Sebastian Monasor L, Verhoeven A, Peters F, Parhizkar S, Kamp F, Gomez de Aguero M, MacPherson AJ, Winkler E, Herms J, Benakis C, Dichgans M, Steiner H, Giera M, Haass C et al. (2021) Microbiota-derived short chain fatty acids modulate microglia and promote Abeta plaque deposition. *Elife* 10: e59826

Dalile B, Van Oudenhove L, Vervliet B, Verbeke K (2019) The role of short-chain fatty acids in microbiota-gut-brain communication. *Nat Rev Gastroenterol Hepatol* 16: 461-478

Erny D, Dokalis N, Mezo C, Castoldi A, Mossad O, Staszewski O, Frosch M, Villa M, Fuchs V, Mayer A, Neuber J, Sosat J, Tholen S, Schilling O, Vlachos A, Blank T, Gomez de Aguero M, Macpherson AJ, Pearce EJ, Prinz M (2021) Microbiota-derived acetate enables the metabolic fitness of the brain innate immune system during health and disease. *Cell Metab* 33: 2260-2276 e7

Erny D, Hrabe de Angelis AL, Jaitin D, Wieghofer P, Staszewski O, David E, Keren-Shaul H, Mahlakoiv T, Jakobshagen K, Buch T, Schwierzeck V, Utermohlen O, Chun E, Garrett WS, McCoy KD, Diefenbach A, Staeheli P, Stecher B, Amit I, Prinz M (2015) Host microbiota constantly control maturation and function of microglia in the CNS. *Nat Neurosci* 18: 965-77

Huuskonen J, Suuronen T, Nuutinen T, Kyrylenko S, Salminen A (2004) Regulation of microglial inflammatory response by sodium butyrate and short-chain fatty acids. *Br J Pharmacol* 141: 874-80

Lee CY, Landreth GE (2010) The role of microglia in amyloid clearance from the AD brain. *J Neural Transm (Vienna)* 117: 949-60

Qian XH, Xie RY, Liu XL, Chen SD, Tang HD (2022) Mechanisms of short-chain fatty acids derived from gut microbiota in Alzheimer's disease. *Aging Dis* 13: 1252-1266

Saito T, Matsuba Y, Mihira N, Takano J, Nilsson P, Itohara S, Iwata N, Saido TC (2014) Single

App knock-in mouse models of Alzheimer's disease. *Nat Neurosci* 17: 661-663

Solito E, Sastre M (2012) Microglia function in Alzheimer's disease. *Front Pharmacol* 3: 14

Xie J, Gorlé N, Vandendriessche C, Van Imschoot G, Van Wonterghem E, Van Cauwenberghe C, Parthoens E, Van Hamme E, Lippens S, Van Hoecke L, Vandenbroucke RE (2021) Low-grade peripheral inflammation affects brain pathology in the $App^{NL\text{-}G\text{-}F}$ mouse model of Alzheimer’s disease. *Acta Neuropathol Commun* 9: 163

Yang Y, Yang LY, Orban L, Cuylear D, Thompson J, Simon B, Yang Y (2018) Non-invasive vagus nerve stimulation reduces blood-brain barrier disruption in a rat model of ischemic stroke. *Brain Stimul* 11: 689-698

2nd Revision - Editorial Decision 13th Mar 2023

Dear Roos,

Thank you for submitting your revised manuscript to The EMBO Journal. Your study has now been seen by referees #1 and 2 and their comments are provided below.

As you can see, both referees appreciate that the revised version has been strengthen and are overall supportive of the study. Referee #2 raises some issues with the vagus data and suggests removing this dataset from the manuscript. I am OK with removing the data, but also feel a bit hesitant about removing sound data from the manuscript. Can we discuss this further?

When you submit the revised version will you also take care of the following editorial points:

- The grant information should also be included in the MS file.

- Please double check the reference style => for articles with more than 10 authors the author list should be cut after 10 authors followed by et al.

- The RNAseq data should be deposited in an archive and the accession number should be added to the Data Availability Section.

- Please remove the Authors Contributions from the manuscript. The 'Author Contributions' section is replaced by the CRediT contributor roles taxonomy to specify the contributions of each author in the journal submission system. Please use the free text box in the 'author information' section of the manuscript submisssion system to provide more detailed descriptions (e.g., 'X provided intracellular Ca++ measurements in fig Y')

- COI should be re-labelled as Disclosure and competing interests statement

- Figure callout for 2E is missing

- Appendix Table S1-S5 should not be zipped, but uploaded separately as Datasets, and renamed to Dataset EV1-EV5 with the corresponding callouts, and legends should be uploaded as separate tabs in each Excel file

- Appendix Table S6 should be renamed to Appendix Table S1, both in ToC and the legend

- Source data files need to be reorganized to one file/folder per figure and ZIPed together for each main figure. For EV and/or appendix figures, the source data should be zipped in one file labelled EV/Appendix source data

- The synopsis image should be 550 wide by [200-400]

- Please upload a synopsis text. We need a summary statement plus 3-5 bullet points describing the key findings of the MS.

- Our publisher has also done their pre-publication check on your manuscript. When you log into the manuscript submission system you will see the file "Data Edited Manuscript file". Please look at the word file and the comments regarding the figure legends and respond to the issues.

Best Karin

Karin Dumstrei, PhD
Senior Editor
The EMBO Journal

Instructions for preparing your revised manuscript:

Guide For Authors: https://www.embopress.org/page/journal/14602075/

authorguide

Referee #1:

The authors have overall answered my questions. While not entirely satisfied, the authors have made a good attempt to meet my concerns. I have therefore no further questions.

Referee #2:

The authors have put in a lot of effort to address the prior concerns that were raised. The manuscript is improved. However, it remains disjointed. The most challenging part to fit in may be the vagus data, which distracts from the other parts of the study (would the author consider removing those data in a revision?). As presented, the first part of the study remains the most compelling where the data (including revised data) clearly support the conclusions drawn.

Referee #1

The authors have overall answered my questions. While not entirely satisfied, the authors have made a good attempt to meet my concerns. I have therefore no further questions.
Thank you for your recognition our efforts. Regarding comment 1 in the previous revision, we originally only included RNA-seq analysis of the choroid plexus of specific pathogen-free (SPF) and antibiotics-treated (AB) mice, as shown in Figure 1. In the newest version of our manuscript, we now also included RNA-seq on choroid plexus from Germ-free (GF) and recolonized GF (GFR) mice. Consequently, two additional comparisons were made: GF *vs* SPF and GFR *vs* GF (**Dataset EV1**). The resulting differentially expressed genes (DEGs) from all four comparisons were integrated into one heatmap (**Appendix Figure S2**). In addition, we submitted all our data in the Gene Expression Omnibus (GEO) with accession number GSE228655 (https://www.ncbi.nlm.nih.gov/geo/query/acc.cgi?acc=GSE228655).

Referee #2

The authors have put in a lot of effort to address the prior concerns that were raised. The manuscript is improved. However, it remains disjointed. The most challenging part to fit in may be the vagus data, which distracts from the other parts of the study (would the author consider removing those data in a revision?). As presented, the first part of the study remains the most compelling where the data (including revised data) clearly support the conclusions drawn.
As mentioned in our previous response letter, the vagus nerve is an intriguing contributor of how the gut (microbiome) affects the brain. Our data further add to the complexity of the gut-brain mechanisms related to both short-chain fatty acids (SCFAs) and the vagus nerve. Indeed, we could show that SCFAs have a direct impact on the tightness of the blood-cerebrospinal fluid (CSF) barrier independent of the vagus nerve, while our findings also demonstrate that vagotomy in specific pathogen-free (SPF) mice is associated with a loss of blood-CSF barrier integrity, as shown in **Figure 5A-F**. Therefore, we believe the vagus nerve data is important and we decided to not remove it from the revised manuscript.

3rd Revision - Editorial Decision 2nd Jun 2023

Dear Roos,

Thank you for submitting your manuscript to The EMBO Journal. I have now had a chance to take a careful look at everything and all looks good.

I am therefore very pleased to accept the manuscript for publication here.

Congratulations on a nice study

with best wishes

Karin

Karin Dumstrei, PhD
Senior Editor
The EMBO Journal

Please note that it is EMBO Journal policy for the transcript of the editorial process (containing referee reports and your response letter) to be published as an online supplement to each paper. If you do NOT want this, you will need to inform the Editorial Office via email immediately. More information is available here:
https://www.embopress.org/page/journal/14602075/authorguide#transparentprocess

Your manuscript will be processed for publication in the journal by EMBO Press. Manuscripts in the PDF and electronic editions of The EMBO Journal will be copy edited, and you will be provided with page proofs prior to publication. Please note that supplementary information is not included in the proofs.

You will be contacted by Wiley Author Services to complete licensing and payment information. The required 'Page Charges Authorization Form' is available here: https://www.embopress.org/pb-assets/embo-site/tej_apc.pdf - please download and complete the form and return to embopressproduction@wiley.com

EMBO Press participates in many Publish and Read agreements that allow authors to publish Open Access with reduced/no publication charges. Check your eligibility: https://authorservices.wiley.com/author-resources/Journal-Authors/open-access/affiliation-policies-payments/index.html

Should you be planning a Press Release on your article, please get in contact with embojournal@wiley.com as early as possible, in order to coordinate publication and release dates.

If you have any questions, please do not hesitate to call or email the Editorial Office. Thank you for your contribution to The EMBO Journal.

EMBO Press Author Checklist

Corresponding Author Name: Roosmarijn E. Vandenbroucke	
Journal Submitted to: The EMBO Journal	
Manuscript Number: EMBOJ-2022-111515R-Q	

USEFUL LINKS FOR COMPLETING THIS FORM
The EMBO Journal - Author Guidelines
EMBO Reports - Author Guidelines
Molecular Systems Biology - Author Guidelines
EMBO Molecular Medicine - Author Guidelines

Reporting Checklist for Life Science Articles (updated January
This checklist is adapted from Materials Design Analysis Reporting (MDAR) Checklist for Authors. MDAR establishes a minimum set of requirements in transparent reporting in the life sciences (see Statement of Task: 10.31222/osf.io/9sm4x). Please follow the journal's guidelines in preparing your manuscript.

Please note that a copy of this checklist will be published alongside your article.

Abridged guidelines for figures
1. Data
The data shown in figures should satisfy the following conditions:
- the data were obtained and processed according to the field's best practice and are presented to reflect the results of the experiments in an accurate and unbiased manner.
- ideally, figure panels should include only measurements that are directly comparable to each other and obtained with the same assay.
- plots include clearly labeled error bars for independent experiments and sample sizes. Unless justified, error bars should not be shown for technical
- if n<5, the individual data points from each experiment should be plotted. Any statistical test employed should be justified.
- Source Data should be included to report the data underlying figures according to the guidelines set out in the authorship guidelines on Data

2. Captions
Each figure caption should contain the following information, for each panel where they are relevant:
- a specification of the experimental system investigated (eg cell line, species name).
- the assay(s) and method(s) used to carry out the reported observations and measurements.
- an explicit mention of the biological and chemical entity(ies) that are being measured.
- an explicit mention of the biological and chemical entity(ies) that are altered/varied/perturbed in a controlled manner.
- the exact sample size (n) for each experimental group/condition, given as a number, not a range;
- a description of the sample collection allowing the reader to understand whether the samples represent technical or biological replicates (including how many animals, litters, cultures, etc.).
- a statement of how many times the experiment shown was independently replicated in the laboratory.
- definitions of statistical methods and measures:
  - common tests, such as t-test (please specify whether paired vs. unpaired), simple χ^2 tests, Wilcoxon and Mann-Whitney tests, can be unambiguously identified by name only, but more complex techniques should be described in the methods section;
  - are tests one-sided or two-sided?
  - are there adjustments for multiple comparisons?
  - exact statistical test results, e.g., P values = x but not P values < x;
  - definition of 'center values' as median or average;
  - definition of error bars as s.d. or s.e.m.

Please complete ALL of the questions below.
Select "Not Applicable" only when the requested information is not relevant for your study.

Materials

Newly Created Materials	Information included in the manuscript?	In which section is the information available? (Reagents and Tools Table, Materials and Methods, Figures, Data Availability Section)
New materials and reagents need to be available; do any restrictions apply?	Not Applicable	

Antibodies	Information included in the manuscript?	In which section is the information available? (Reagents and Tools Table, Materials and Methods, Figures, Data Availability Section)
For **antibodies** provide the following information:<br>- Commercial antibodies: RRID (if possible) or supplier name, catalogue number and or/clone number<br>- Non-commercial: RRID or citation	Yes	Material and Methods

DNA and RNA sequences	Information included in the manuscript?	In which section is the information available? (Reagents and Tools Table, Materials and Methods, Figures, Data Availability Section)
**Short novel DNA or RNA including primers, probes**: provide the sequences.	Yes	Appendix table

Cell materials	Information included in the manuscript?	In which section is the information available? (Reagents and Tools Table, Materials and Methods, Figures, Data Availability Section)
**Cell lines:** Provide species information, strain. Provide accession number in repository **OR** supplier name, catalog number, clone number, and/**OR** RRID.	Not Applicable	
**Primary cultures:** Provide species, strain, sex of origin, genetic modification status.	Yes	Material and Methods
Report if the cell lines were recently **authenticated** (e.g., by STR profiling) and tested for mycoplasma contamination.	Not Applicable	

Experimental animals	Information included in the manuscript?	In which section is the information available? (Reagents and Tools Table, Materials and Methods, Figures, Data Availability Section)
**Laboratory animals or Model organisms:** Provide species, strain, sex, age, genetic modification status. Provide accession number in repository **OR** supplier name, catalog number, clone number, **OR** RRID.	Yes	Material and Methods
**Animal observed in or captured from the field:** Provide species, sex, and age where possible.	Not Applicable	
Please detail **housing and husbandry conditions**.	Not Applicable	

Plants and microbes	Information included in the manuscript?	In which section is the information available? (Reagents and Tools Table, Materials and Methods, Figures, Data Availability Section)
**Plants:** provide species and strain, ecotype and cultivar where relevant, unique accession number if available, and source (including location for collected wild specimens).	Not Applicable	
**Microbes:** provide species and strain, unique accession number if available, and source.	Not Applicable	

Human research participants	Information included in the manuscript?	In which section is the information available? (Reagents and Tools Table, Materials and Methods, Figures, Data Availability Section)
If collected and within the bounds of privacy constraints report on age, sex and gender or ethnicity for all study participants.	Not Applicable	

Core facilities	Information included in the manuscript?	In which section is the information available? (Reagents and Tools Table, Materials and Methods, Figures, Data Availability Section)
If your work benefited from core facilities, was their service mentioned in the acknowledgments section?	Yes	Acknowledgements

Design

Study protocol	Information included in the manuscript?	In which section is the information available? (Reagents and Tools Table, Materials and Methods, Figures, Data Availability Section)
If study protocol has been **pre-registered, provide DOI in the manuscript**. For clinical trials, provide the trial registration number **OR** cite DOI.	Not Applicable	
Report the **clinical trial registration number** (at ClinicalTrials.gov or equivalent), where applicable.	Not Applicable	

Laboratory protocol	Information included in the manuscript?	In which section is the information available? (Reagents and Tools Table, Materials and Methods, Figures, Data Availability Section)
Provide DOI OR other citation details if **external detailed step-by-step protocols** are available.	Not Applicable	

Experimental study design and statistics	Information included in the manuscript?	In which section is the information available? (Reagents and Tools Table, Materials and Methods, Figures, Data Availability Section)
Include a statement about **sample size** estimate even if no statistical methods were used.	Yes	Figures and Figure legends
Were any steps taken to minimize the effects of subjective bias when allocating animals/samples to treatment (e.g. **randomization procedure**)? If yes, have they been described?	Yes	Material and Methods
Include a statement about **blinding** even if no blinding was done.	Not Applicable	
Describe **inclusion/exclusion criteria** if samples or animals were excluded from the analysis. Were the criteria pre-established?<br><br>If sample or data points were omitted from analysis, report if this was due to attrition or intentional exclusion and provide justification.	Yes	Material and Methods
For every figure, are **statistical tests** justified as appropriate? Do the data meet the assumptions of the tests (e.g., normal distribution)? Describe any methods used to assess it. Is there an estimate of variation within each group of data? Is the variance similar between the groups that are being statistically compared?	Yes	Figures and Figure legends; Materials and Methods

Sample definition and in-laboratory replication	Information included in the manuscript?	In which section is the information available? (Reagents and Tools Table, Materials and Methods, Figures, Data Availability Section)
In the figure legends: state number of times the experiment was **replicated** in laboratory.	Yes	Figure and Figure legends; Materials and Methods
In the figure legends: define whether data describe **technical or biological replicates**.	Yes	Figure and Figure legends; Materials and Methods

Ethics

Ethics	Information included in the manuscript?	In which section is the information available? (Reagents and Tools Table, Materials and Methods, Figures, Data Availability Section)
Studies involving **human participants**: State details of **authority granting ethics approval** (IRB or equivalent committee(s), provide reference number for approval.	Not Applicable	
Studies involving **human participants**: Include a statement confirming that **informed consent** was obtained from all subjects and that the experiments conformed to the principles set out in the WMA Declaration of Helsinki and the Department of Health and Human Services Belmont Report.	Not Applicable	
Studies involving **human participants:** For publication of **patient photos**, include a statement confirming that consent to publish was obtained.	Not Applicable	
Studies involving experimental **animals**: State details of **authority granting ethics approval** (IRB or equivalent committee(s), provide reference number for approval. Include a statement of compliance with ethical regulations.	Yes	Materials and Methods
Studies involving **specimen and field samples:** State if relevant **permits** obtained, provide details of authority approving study; if none were required, explain why.	Not Applicable	

Dual Use Research of Concern (DURC)	Information included in the manuscript?	In which section is the information available? (Reagents and Tools Table, Materials and Methods, Figures, Data Availability Section)
Could your study fall under dual use research restrictions? Please check biosecurity documents and list of **select agents and toxins** (CDC): https://www.selectagents.gov/sat/list.htm	Not Applicable	
If you used a select agent, is the security level of the lab appropriate and reported in the manuscript?	Not Applicable	
If a study is subject to dual use research of concern regulations, is the name of the **authority granting approval and reference number** for the regulatory approval provided in the manuscript?	Not Applicable	

Reporting

The MDAR framework recommends adoption of discipline-specific guidelines, established and endorsed through community initiatives. Journals have their own policy about requiring specific guidelines and recommendations to complement MDAR.

Adherence to community standards	Information included in the manuscript?	In which section is the information available? (Reagents and Tools Table, Materials and Methods, Figures, Data Availability Section)
State if relevant guidelines or checklists (e.g., **ICMJE, MIBBI, ARRIVE, PRISMA**) have been followed or provided.	Not Applicable	
For **tumor marker prognostic studies,** we recommend that you follow the **REMARK** reporting guidelines (see link list at top right). See author guidelines, under 'Reporting Guidelines'. Please confirm you have followed these guidelines.	Not Applicable	
For **phase II and III randomized controlled trials**, please refer to the **CONSORT** flow diagram (see link list at top right) and submit the CONSORT checklist (see link list at top right) with your submission. See author guidelines, under 'Reporting Guidelines'. Please confirm you have submitted this list.	Not Applicable	

Data Availability

Data availability	Information included in the manuscript?	In which section is the information available? (Reagents and Tools Table, Materials and Methods, Figures, Data Availability Section)
Have **primary datasets** been deposited according to the journal's guidelines (see 'Data Deposition' section) and the respective accession numbers provided in the Data Availability Section?	Yes	Data Availability
Were **human clinical and genomic datasets** deposited in a public access-controlled repository in accordance to ethical obligations to the patients and to the applicable consent agreement?	Not Applicable	
Are **computational models** that are central and integral to a study available without restrictions in a machine-readable form? Were the relevant accession numbers or links provided?	Not Applicable	
If publicly available data were reused, provide the respective **data citations in the reference list**.	Not Applicable	
